# A palladium-catalyzed C–H functionalization route to ketones *via* the oxidative coupling of arenes with carbon monoxide†

**DOI:** 10.1039/d0sc00085j

**Published:** 2020-02-20

**Authors:** Taleah M. Levesque, R. Garrison Kinney, Bruce A. Arndtsen

**Affiliations:** Department of Chemistry, McGill University 801 Sherbrooke Street West Montreal QC H3A 0B8 Canada bruce.arndtsen@mcgill.ca

## Abstract

We describe the development of a new palladium-catalyzed method to generate ketones *via* the oxidative coupling of two arenes and CO. This transformation is catalyzed by simple palladium salts, and is postulated to proceed *via* the conversion of arenes into high energy aroyl triflate electrophiles. Exploiting the latter can also allow the synthesis of unsymmetrical ketones from two different arenes.

## Abstract 2

A palladium catalyzed route to prepare aryl ketones from their two fundamental building blocks, two arenes and carbon monoxide, is described.

## Graphical abstract

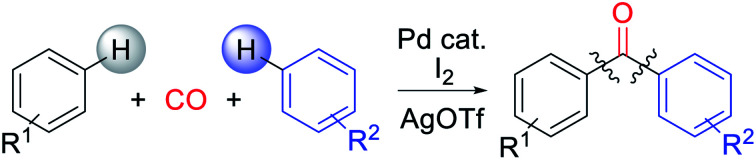

## Introduction

**Fig. 1:**
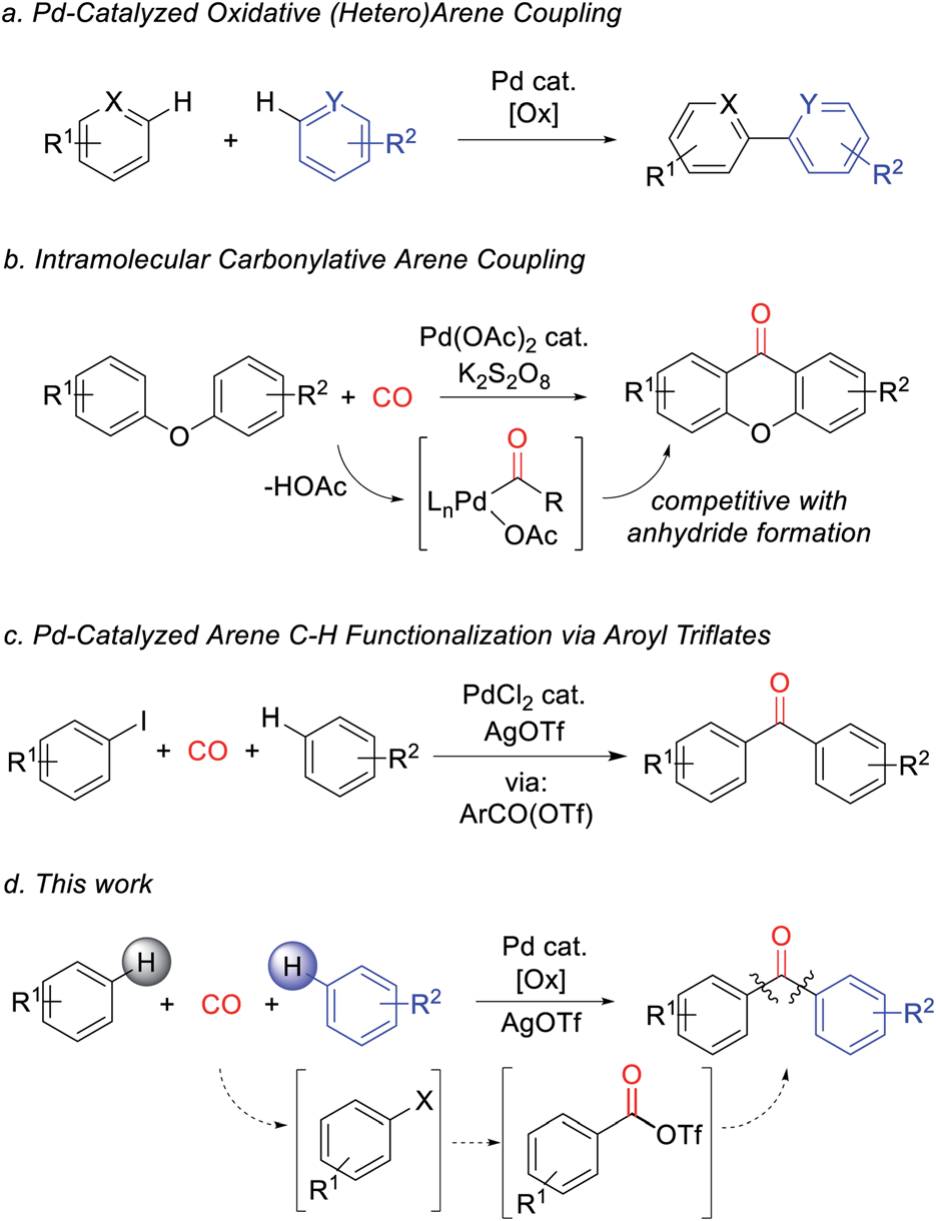
Approaches to intra- and intermolecular oxidative C–H functionalization and application to ketone synthesis. (a) Oxidative arene coupling. (b) Intramolecular carbonylative arene coupling. (c) Pd-catalyzed arene functionalization with aroyl triflates. (d) This work.

Metal-catalyzed arene C–H bond functionalization has emerged as a powerful technique for the efficient and atom-economical build-up of organic products from feedstock chemicals.^1^ One important class of these reactions is to form carbon–carbon bonds from the oxidative coupling of two separate C–H bond containing substrates.^2^ In contrast to classical synthetic methods employing pre-functionalized substrates (*e.g.* cross-coupling reactions), this strategy offers a platform to assemble biaryls and related structures directly from arenes with high step efficiency and minimal waste (Fig. 1a).^3^ A potentially useful variant would be to perform these in concert with reactive units such as carbon monoxide. Carbon monoxide is a broadly available C1 building block, and its coupling with organic fragments leads to the formation of valuable carbonyl-containing products.^4^ Moreover, unlike inert aryl–aryl bonds, carbonyl derivatives such as ketones number among the most synthetically versatile functionalities in organic chemistry. The palladium-catalyzed carbonylative functionalization of aryl or even alkyl C–H bonds is well established, and dates back to early work by Fujiwara and others.^5^ However, these reactions commonly lead to carboxylic acids or their derivatives (*e.g.* esters, amides), and are rarely applicable to the assembly of ketones.^6^ This limitation has been attributed in part to the favored reaction of Pd-acyl intermediates with the carboxylate ligands that are often required for the C–H palladation step, therefore leading instead to the formation of anhydrides.^7^ As shown by Lei, this can be avoided by performing oxidative C–H functionalization in an intramolecular fashion (Fig. 1b).^8^ Nevertheless, the ability to assemble aryl ketones *via* the simple coupling of its two most fundamental building blocks, arenes and carbon monoxide, has to date presented an unmet challenge.^9,10^

We have recently described an alternative approach to carbonylative C–H functionalization, wherein palladium catalysts can mediate the *in situ* formation of reactive aroyl electrophiles such as acyl iodides and even acyl triflates.^11^ The latter have the ability to react with unactivated arenes and allowed the development of a carbonylative method to functionalize arenes to form ketones (Fig. 1c). In considering this chemistry, we questioned if this approach could be taken even further and offer a pathway to perform carbonylative arene/arene coupling with carbon monoxide. In principle, this might be accomplished by devising a method to replace aryl iodides with an oxidative carbonylative platform to convert arenes themselves to high energy acyl triflate electrophiles (Fig. 1d). We describe herein our studies towards such a system. This has led to the discovery of the first catalytic route to assemble ketones directly from two arenes and carbon monoxide. Mechanistic studies suggest this reaction proceeds *via* a series of steps that ultimately convert arenes into high energy aroyl triflate electrophiles. Exploitation of the latter has opened a novel route to generate heterocoupled ketones from two separate arenes.

## Results and discussion

**Scheme 1:**
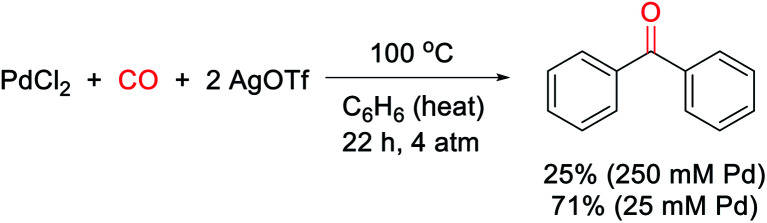
Generation of benzophenone *via* stoichiometric, PdCl_2_-mediated arene/CO coupling.

Our research on this topic grew out of a study on the mechanism of the Pd(0)-catalyzed carbonylative formation of aroyl triflate electrophiles from aryl iodides.^11*d*^ This reaction can be performed using PdCl_2_ and related Pd(ii) catalyst precursors (Fig. 1c), but it was unclear how these Pd(ii) sources are reduced to Pd(0) to mediate the reaction. Carbon monoxide has been established to reduce Pd(ii) to Pd(0).^12,13^ However, this often occurs in the presence of nucleophiles (*e.g.* water) that can trap the oxidized carbon monoxide fragment, and the reaction conditions did not include an obvious nucleophile, or even a dative ligand (*e.g.* phosphines) that might be oxidized by Pd(ii). To probe this, the stoichiometric reaction of PdCl_2_ with carbon monoxide and AgOTf was examined in benzene. To our surprise, this leads to the formation of an aromatic product, benzophenone, in up to 71% yield (Scheme 1).

The ability of metal salts such as PdCl_2_ to mediate the oxidative carbonylation of arenes to ketones is to our knowledge unknown.^13^ While there are several pathways by which this stoichiometric reaction may occur (*vide infra*), the formal by-product of this reaction is Pd(0), suggesting that a suitable oxidant may transform this stoichiometric chemistry into a catalytic route to form aryl ketones. As shown in Table 1, the addition of various metal salt oxidants that are commonly employed in related oxidative coupling chemistry to the reaction of benzene and CO with 10 mol% PdCl_2_ catalyst leads to minimal reaction (entries 1–3), and also inhibits the stoichiometric formation of benzophenone. However, we were pleased to find that simple iodine is an effective oxidant and allows the catalytic formation of benzophenone in good yield (79%, entry 4). Further experimentation showed that the reaction does not require the use of arene as solvent and proceeds in high yield with a near stoichiometric amount of arene in 1,2-dichloroethane (92%, entry 5) and at temperatures as low as 60 °C with 1 atm CO (entries 6–10). The reaction here was performed with *t*-butylbenzene to aid *in situ* characterization, but is equally efficient with benzene (entry 11). Simple [Pd(allyl)Cl]_2_ is the most effective catalytic precursor under these conditions,^14^ and the reaction can be performed with as little as 0.1 mol% [Pd(allyl)Cl]_2_ at elevated temperatures (entry 12).

**Table 1:** Development of conditions for carbonylative homocoupling of arenes^a^

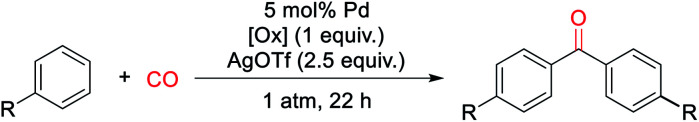						
Entry	Pd cat.	Ligand	Temp.	Solvent	[Ox]	Yield (%)
1^b^	PdCl_2_	—	100 °C	C_6_H_6_	Ce(OTf)_4_	0
2^b^	PdCl_2_	—	100 °C	C_6_H_6_	Na_2_S_2_O_8_	2
3^b^	PdCl_2_	—	100 °C	C_6_H_6_	CuCl_2_	4
4^b^	PdCl_2_	—	100 °C	C_6_H_6_	I_2_	79
5^b^	PdCl_2_	—	100 °C	1,2-DCE	I_2_	92
6	PdCl_2_	—	60 °C	1,2-DCE	I_2_	23
7	Pd(OAc)_2_	—	60 °C	1,2-DCE	I_2_	60
8	[Pd(allyl)Cl]_2_	PPh_3_^c^	60 °C	1,2-DCE	I_2_	78
9	[Pd(allyl)Cl]_2_	P^*t*^Bu_3_^c^	60 °C	1,2-DCE	I_2_	57
10	[Pd(allyl)Cl]_2_	—	60 °C	1,2-DCE	I_2_	87
11	[Pd(allyl)Cl]_2_	—	60 °C	1,2-DCE	I_2_	93 (94)^d^
12	[Pd(allyl)Cl]_2_^e^	—	100 °C	1,2-DCE	I_2_	72

a Conditions: arene (0.53 mmol), silver triflate (176 mg, 0.68 mmol), Ox (0.25 mmol), 5 mol% Pd, 1 atm CO, 22 h, R = ^*t*^Bu.

b 10 mol% Pd, 4 atm CO, R = H (entries 1–4), ^*t*^Bu (entry 5).

c 10 mol%.

d 0.75 mmol arene, R = ^*t*^Bu (R = H in brackets).

e 0.1 mol% [Pd(allyl)Cl]_2_.

With a palladium-catalyzed method to assemble ketones from arenes and carbon monoxide in hand, we next turned to probing its generality. As shown in Table 2, various electron neutral and electron rich arenes can be employed in this reaction and form ketones in high yields. These include alkyl-substituted arenes (**1b**, **1c**, **1f**) as well as more electron rich anisole derivatives (**1i**, **1j**). In each of the mono-substituted arenes, we observed 4,4′-substituted ketones as the major isolated product, consistent with an electrophilic mechanism for functionalization. Electron deficient substrates, such as mono- and di-halogenated arenes, react under more pressing conditions to form ketones in neat arene (**1d**, **1e**, **1k**). More sterically hindered 1,3- and 1,4-di-substituted arenes can also be converted to ketones (**1f–h**, **1k**, **1l**). In these cases, functionalization proceeds again at the most electron rich site on the arene. This oxidative carbonylation can also be applied to the synthesis of ketones from heteroarenes, with examples including thiophenes (**1n**, **1o**), benzothiophene (**1m**), and *N*-tosyl pyrrole (**1p**). The addition of 2,6-^*t*^butylpyridine base is required for these latter examples to inhibit substrate decomposition. Overall, this provides what is to our knowledge the first general catalytic method to assemble aryl ketones in one step from their two most fundamental building blocks, arenes and carbon monoxide, and without the required use of the synthetic, expensive, and/or unstable (hetero)aryl iodides (and organometallic reagents) commonly employed in carbonylative ketone synthesis.

**Table 2:** Palladium-catalyzed oxidative arene/CO/arene coupling to form symmetrical ketones^a^

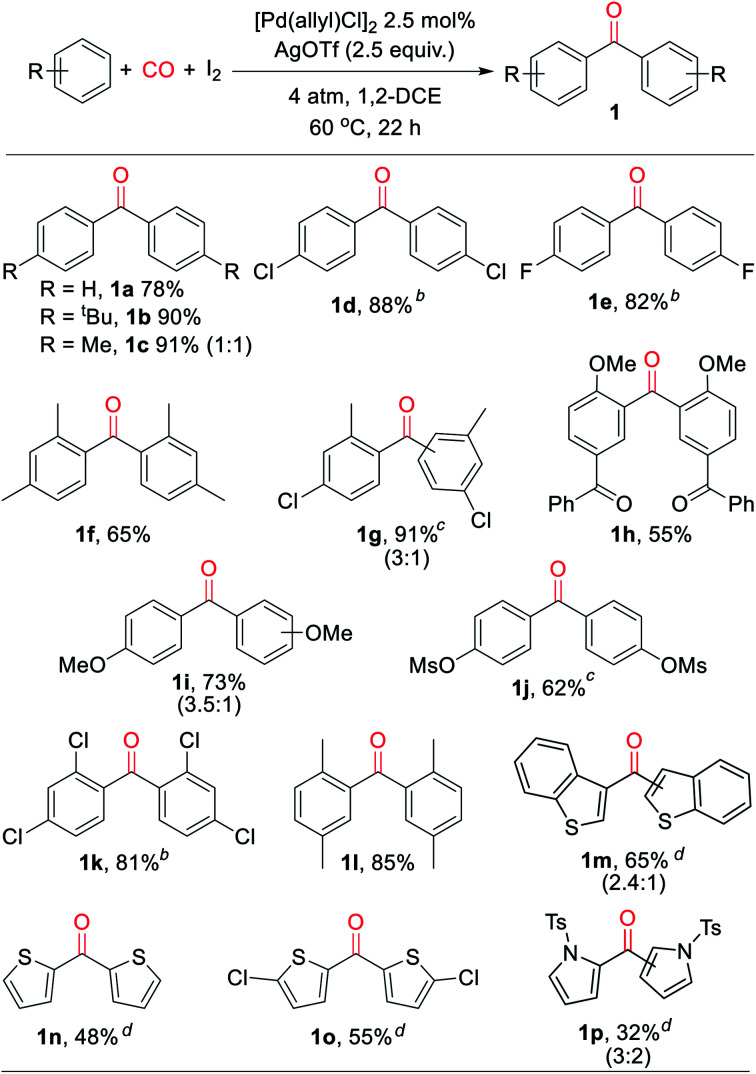

a Conditions: arene (1.50 mmol), AgOTf (350 mg, 1.36 mmol), I_2_ (126 mg, 0.50 mmol), [Pd(allyl)Cl]_2_ (5 mg, 0.013 mmol), 1,2-dichloroethane (2 mL), 4 atm CO, 22 h, 60 °C (see ESI for details). Isolated yield (ratio of 4′ : 2′ isomers).

b At 150 °C in arene solvent.

c At 100 °C.

d With 2,6-di-*tert*-butylpyridine (258 mL, 1.15 mmol) (ratio of 3′ : 2′ isomers).

**Fig. 2:**
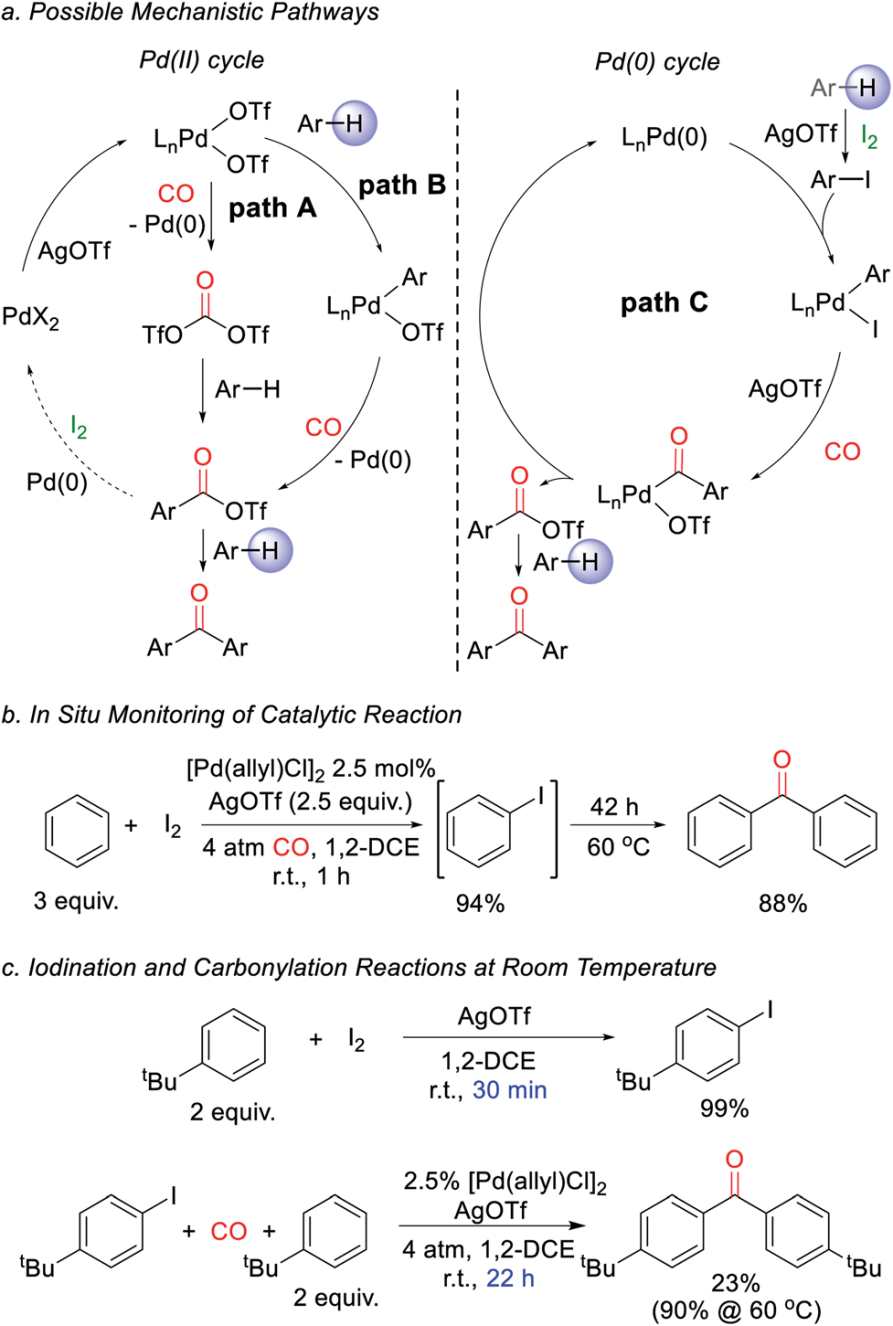
Proposed catalytic pathway for ketone formation. (a) Potential mechanisms. (b) *In situ* monitoring of reaction. (c) Relative rates of iodination and carbonylation.

We next turned to examine how this transformation proceeds. As mechanistic possibilities, the stoichiometric reaction of PdCl_2_ (Scheme 1) suggests the potential *in situ* formation of phosgene equivalents upon reduction of Pd(ii) by CO (Fig. 2a, path A),^13^ or the build-up of electrophilic Pd(ii) triflate intermediates that may directly palladate arenes (path B).^15^ These could each lead to aroyl triflate intermediates for subsequent Friedel–Crafts acylations,^11*d*^ and are consistent with the regioselectivity in the ketone products formed. The re-oxidation of Pd(0) by I_2_ would then make these viable cycles for catalysis. However, iodine is also established to react with arenes in the presence of silver triflate to form aryl iodides.^16^ It is therefore possible that the catalytic arene carbonylation occurs *via* an alternative Pd(0) cycle to form aroyl triflate products (path C). Monitoring the reaction by *in situ*^1^H and ^13^C NMR analysis show that the latter mechanism is likely the case during catalysis, and we observe the rapid, high yield build-up of aryl iodide within 1 h under the catalytic conditions (Fig. 2b, 94% yield). Continued heating converts this aryl iodide to ketone **1a**. Similarly, control experiments show that the AgOTf mediated iodination of the arene is rapid (30 min at r.t.), relative to catalytic ketone formation (23% after 22 h at r.t.; Fig. 2c). Thus, in the presence of I_2_ as oxidant, these data suggest that the direct iodination of the arene is the most rapid pathway (path C). Nevertheless, the ability of Pd(ii) salts themselves to mediate the direct carbonylative C–H functionalization of arenes also appears to be relevant to catalysis, as it allows the activation of the Pd(ii) pre-catalyst (*e.g.*Table 1). The compatibility between Pd(0) catalysis and I_2_ is unusual, since Pd(0) can also undergo rapid oxidation. This presumably reflects the rapid rate of arene iodination relative to the slower carbonylation and build-up of Pd(0), as well as the ability of any *in situ* generated Pd(ii) to re-enter the cycle (*e.g. via* paths A or B). The lack of potentially oxidizable donor ligands in the reaction further enhances this compatibility. Together, this suggests a new avenue to use Pd(0) catalysis in oxidative coupling chemistry with I_2_.

From a synthetic perspective, the generation of halogenated arene intermediates can have useful implications. Since these intermediates are formed more rapidly than carbonylation, the rate difference can be exploited to perform cross arene/arene coupling to build up more structurally diverse ketones. The latter can present a challenge with many oxidative arene/arene coupling reactions, and typically require significant differences in the electronic, steric, or coordinating ability of the two substrates.^2^ After examination of various reaction conditions, we were pleased to find that the ambient temperature reaction of arene, [Pd(allyl)Cl]_2_ catalyst, I_2_, and AgOTf, followed by the subsequent addition of a second, more electron rich arene and carbon monoxide leads to the clean overall heterocoupling of arenes to ketones (Table 3). Both electron neutral and electron rich arenes can be easily cross-coupled to form unsymmetrical ketones in high yield, including sterically encumbered tetra-substituted arenes and an activated benzophenone (**2f**, **2h**, **2l**). Employing more pressing conditions allows for the use of deactivated arenes such as bromo- or dichlorobenzene (**2e**, **2g**), and does so without secondary activation of these carbon–halogen bonds. Thiophene, N-substituted pyrrole, and benzothiophene can also be incorporated into this reaction to create mixed (hetero)aryl ketones (**2k**, **2m**, **2n**).

**Table 3:** Palladium-catalyzed oxidative arene/CO/arene coupling to form unsymmetrical ketones^a^

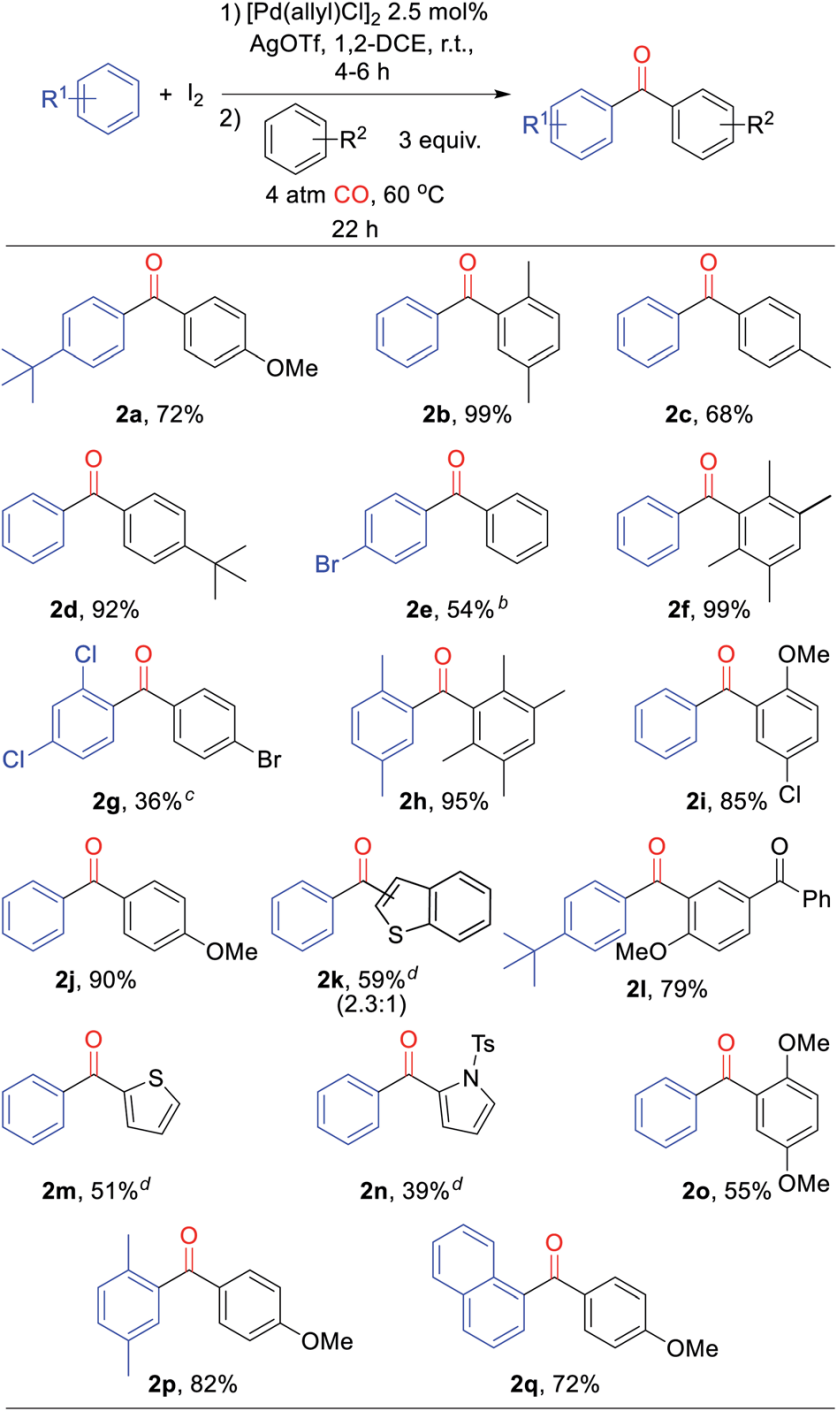

a Conditions: arene (1.00 mmol), AgOTf (339 mg, 1.32 mmol), I_2_ (126 mg, 0.49 mmol), [Pd(allyl)Cl]_2_ (5 mg, 0.013 mmol), 1,2-dichloroethane (2 mL), 4–6 h, r.t., then second arene (1.50 mmol), 4 atm CO, 22 h, 60 °C (see ESI for details). Isolated yields (3′ : 2′ ratio).

b Both steps at 100 °C.

c Step 1 at 100 °C, 22 h; step 2 at 150 °C w/10 eq. bromobenzene.

d With 2,6-di-*tert*-butylpyridine (1.15 mmol).

**Scheme 2:**
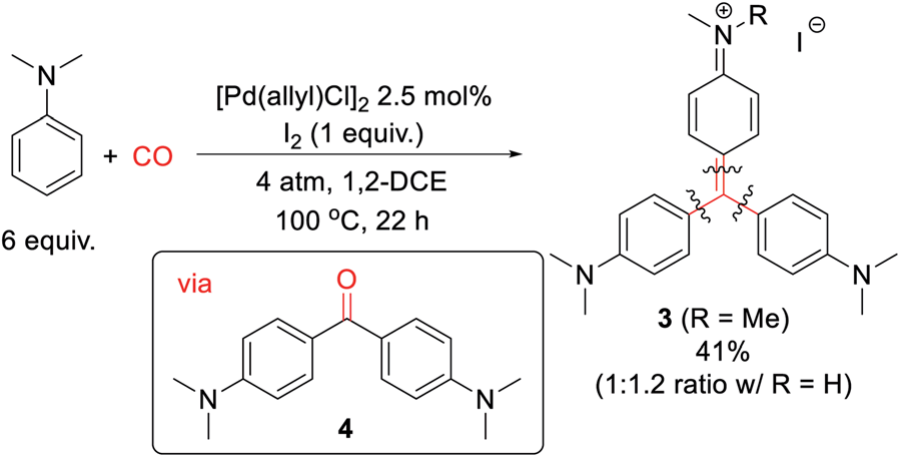
Synthesis of crystal violet from dimethylaniline and carbon monoxide.

Finally, we have examined the use of this system to assemble more structurally elaborate products. As an example, crystal violet (**3**, Scheme 2) is a common dye exploited in textiles. Crystal violet is typically generated starting from reactive, synthetic acylating agents (*e.g.* phosgene), in a sequence that proceeds through the initial build-up of ketone **4**.^17^ Alternatively, the palladium-catalyzed oxidative carbonylation of *N*,*N*-dimethylaniline can offer a method to generate **3** from the parent arene and carbon monoxide (Scheme 2). While **3** is generated in moderate yield (41% together with its de-methylated isomer), it represents the product of three concurrent arene C–H bond functionalization steps. This reaction presumably proceeds *via* the initial generation of **4** that undergoes a spontaneous Friedel–Crafts reaction to liberate H_2_O, which can also be trapped by the *in situ* generated aroyl electrophiles. As far as we are aware, this is the first synthesis of such an advanced structure directly from arenes and CO.

## Conclusions

In conclusion, we have described herein the first catalytic route to prepare ketones from arenes and CO. The reaction generates either symmetrical or unsymmetrical aryl ketones in high yields does so from combinations of stable, commercially available, and easily modulated reagents, and without the need for synthetic and often expensive aryl iodide reagents. Mechanistic analysis suggests this transformation proceeds *via* the palladium-catalyzed, *in situ* generation of potent acylating electrophiles (acyl triflates) from arenes. Considering the broad utility of acylating electrophiles, we anticipate that the ability to access these intermediates from arenes could prove relevant to the design of various new approaches to carbonylative C–H bond functionalization chemistry.

## Conflicts of interest

There are no conflicts to declare.

## Supplementary Material

SC-011-D0SC00085J-s001
