# Supplementary material for: A palladium-catalyzed C–H functionalization route to ketones via the oxidative coupling of arenes with carbon monoxide
Source: Chem Sci. 2020 Feb 20;11(11):3104–9. doi: 10.1039/d0sc00085j (PMC8157493; doi:10.1039/d0sc00085j)

Electronic Supplementary Material (ESI) for Chemical Science.
This journal is © The Royal Society of Chemistry 2020

A Palladium-Catalyzed C-H Functionalization Route to Ketones via the Oxidative Coupling of Arenes with Carbon Monoxide

Taleah M. Levesque, R. Garrison Kinney, and Bruce A. Arndtsen

Department of Chemistry, McGill University, 801 Sherbrooke Street West,

Montreal, QC, Canada, H3A 0B8

Supporting Information

I. General Considerations.......... 1

II. General Synthetic Procedures 2

III. Mechanistic Studies 7

IV. Spectroscopic Data for Compounds **1** 11

V. Spectroscopic Data for Compounds **2**.......... 16

VI. Spectroscopic Data for Compounds **3** 21

VII. References 22

VIII. NMR Spectra.......... 23

I. General Considerations

All manipulations were carried out in an inert atmosphere glovebox or using standard Schlenk techniques unless stated otherwise. Research grade carbon monoxide (99.5%) was used as received. Solvents were collected under nitrogen from a Solvent Purification System and stored over activated 4 Å molecular sieves. Deuterated solvents were dried over calcium hydride, vacuum transferred, and stored over activated 4 Å molecular sieves. Silver triflate was dried by heating to 100 °C under vacuum for 24 h, and then stored in the glovebox. Iodine was dried by

grinding with calcium oxide for 10 min before allowing to sit overnight; then sublimed and immediately stored under nitrogen at -36 °C. All other reagents were purchased from commercial suppliers and used as received after thoroughly drying to remove all traces of water. This was typically done by either dissolving solids and storing over 4 Å molecular sieves overnight before filtration and removal of solvent to yield solid that is dried under vacuum overnight, or by removing oxygen from liquids via freeze-pump-thaw techniques and subsequent storage over 4 Å molecular sieves. ^{1}H nuclear magnetic resonance (NMR) characterization was performed on 400 and 500 MHz spectrometers (101 and 126 MHz for ^{13}C NMR). High-resolution mass spectra were obtained using a quadrupole-time of flight and an orbitrap detector by direct infusion in positive ESI mode or by atmospheric pressure chemical ionization.

II. General Synthetic Procedures

Stoichiometric Reaction of $PdCl_2$ with Carbon Monoxide in Benzene (Scheme 1):

In a glove box, silver triflate (184 mg, 0.72 mmol) was added to a 50 mL thick-walled, glass reaction vessel sealable with a Teflon stopcock and equipped with a stir bar. $PdCl_2$ (44 mg, 0.25 mmol) was dissolved in 10 mL benzene and added to the reaction vessel. The vessel was sealed, removed from the glove box, evacuated and backfilled three times with carbon monoxide, and finally pressurized with 4 atm carbon monoxide. The contents of the reaction vessel were stirred and heated at 100 °C for 22 h, after which the carbon monoxide was released in a fume hood. Hexamethylbenzene (5 mg, 0.033 mmol) was then added to the reaction vessel as a standard, the mixture was filtered through a pad of silica to remove any solids, and the reaction vessel was thoroughly rinsed with $CHCl_3$ followed by ethyl acetate. The solvents were removed *in vacuo* to yield ketone **1a** in 71% yield as determined by ^{1}H NMR spectral analysis relative to the external standard.

*In situ Benzophenone **1a**:* ^{1}H NMR (400 MHz, $CDCl_3$): δ 7.79 (d, J = 7.0 Hz, 2H), 7.58 (t, J = 7.4 Hz, 1H), 7.47 (t, J = 7.6 Hz, 2H). ^{13}C NMR (101 MHz, $CDCl_3$): δ 197.0, 137.7, 132.6, 130.2, 128.4. NMR data corresponds with the previously reported compound.[1]

Crude Mixture ^{1}H and ^{13}C NMR Spectra:

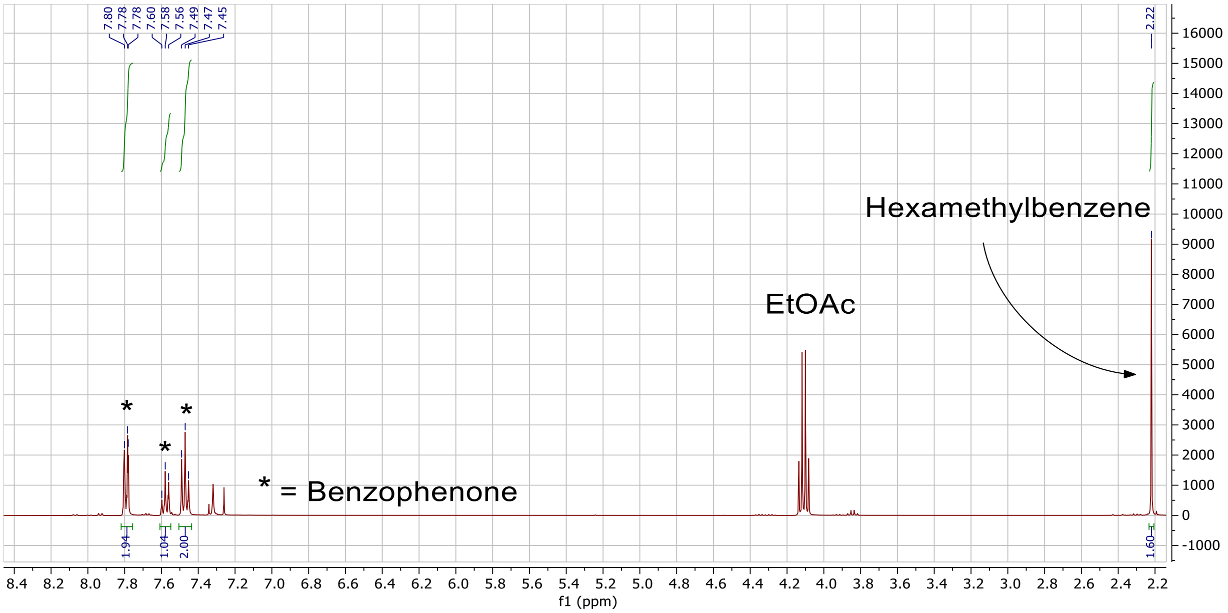

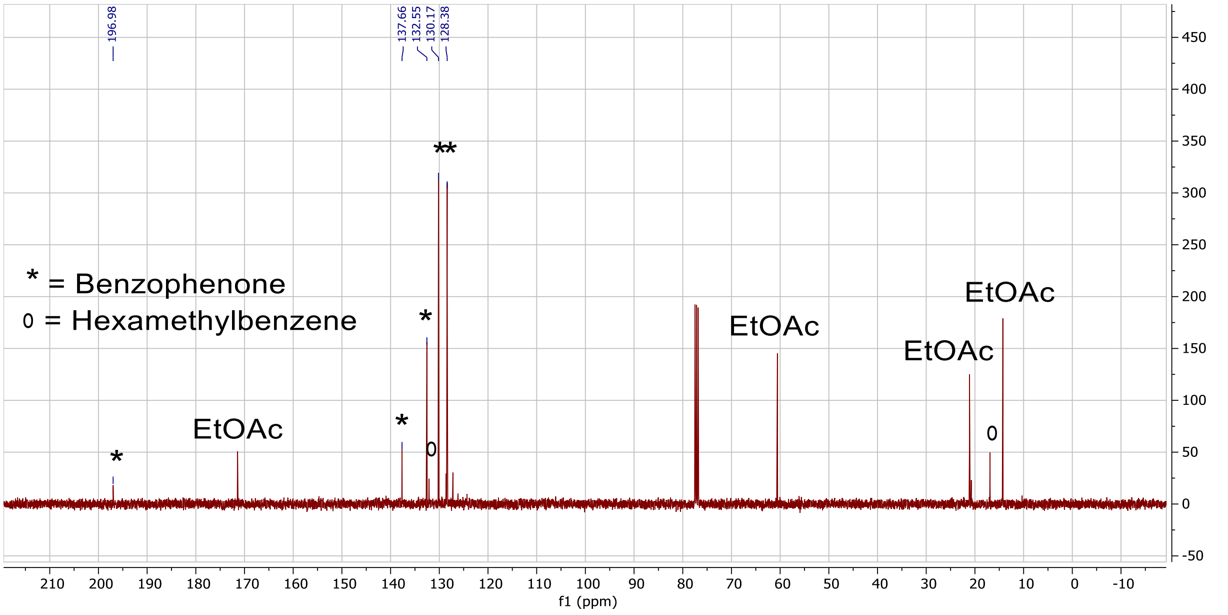

Typical Procedure for Catalyst Development in Neat Benzene (Table 1, Entries 1-4):

In a glove box, silver triflate (198 mg, 0.77 mmol) and iodine (63 mg, 0.25 mmol) were dry transferred into a 50 mL thick-walled, glass reaction vessel sealable with a Teflon stopcock and equipped with a stir bar. $PdCl_2$ (4 mg, 0.025 mmol) and the residual iodine left after dry transferring were dissolved in 1 mL of benzene and added to the reaction vessel. The vessel was

sealed, removed from the glove box, evacuated and backfilled three times with carbon monoxide, and finally pressurized with 4 atm carbon monoxide. The contents of the reaction vessel were stirred and heated at 100 °C for 22 h, after which the carbon monoxide was released in a fume hood. Hexamethylbenzene (6 mg, 0.036 mmol) was then added to the reaction vessel as a standard, the mixture was filtered through a pad of silica to remove any solids, and the reaction vessel was thoroughly rinsed with $CHCl_3$ followed by ethyl acetate. The solvents were removed *in vacuo* to yield ketone **1a** in 79% yield as determined by ^{1}H NMR spectral analysis relative to the external standard.

Typical Procedure for Catalyst Development in 1,2-Dichloroethane (Table 1, Entries 5-12):

In a glove box, silver triflate (182 mg, 0.71 mmol) and iodine (63 mg, 0.25 mmol) were dry transferred into a 50 mL thick-walled, glass reaction vessel sealable with a Teflon stopcock and equipped with a stir bar. $[Pd(allyl)Cl]_2$ (2 mg, 0.0066 mmol) and the residual iodine left after dry transferring was dissolved in 1 mL of 1,2-dichloroethane and added to the reaction vessel followed by *tert*-butylbenzene (81 µL, 0.52 mmol). The vessel was sealed, removed from the glove box, evacuated and backfilled three times with carbon monoxide, and finally pressurized with 1 atm carbon monoxide. The contents of the reaction vessel were stirred and heated at 60 °C for 22 h, after which the carbon monoxide was released in a fume hood. Hexamethylbenzene (8 mg, 0.046 mmol) was then added to the reaction vessel as a standard, the mixture was filtered through a pad of silica to remove any solids, and the reaction vessel was thoroughly rinsed with $CHCl_3$ followed by ethyl acetate. The solvents were removed *in vacuo* to yield ketone **1b** in 87% yield as determined by ^{1}H NMR spectral analysis ($CDCl_3$) relative to the external standard.

Procedure for the Synthesis of Ketones in Table 2:

All compounds in Table 2 were prepared according to the procedure detailed below. See the tabulated NMR data for any adjustments to the procedure employed or reaction temperature. For compounds **1m**, **1n**, **1o**, and **1p**, 2,6-di-*tert*-butylpyridine (258 µL, 1.15 mmol) was also added to quench *in situ* generated triflic acid and prevent side reactions.

Representative Procedure: In a glove box, silver triflate (350 mg, 1.36 mmol) and iodine (126 mg, 0.50 mmol) were dry transferred into a 50 mL thick-walled glass reaction vessel sealable with a Teflon stopcock and equipped with a magnetic stir bar. $[Pd(allyl)Cl]_2$ (5 mg, 0.013 mmol) and the residual iodine left after dry transferring were dissolved in 2 mL of 1,2-dichloroethane and added to the reaction vessel. Benzene was added to the reaction vessel via micropipette (134 µL, 1.50 mmol). The vessel was sealed, removed from the glove box, evacuated and backfilled three times with carbon monoxide, and finally pressurized with 4 atm carbon monoxide. The reaction mixture was stirred and heated at 60 °C for 22 h, after which the carbon monoxide was released in a fume hood. The reaction mixture was filtered through a pad of silica, and the reaction vessel was thoroughly rinsed with $CHCl_3$ followed by ethyl acetate. The solvents were removed *in vacuo* and the product was purified via column chromatography (silica gel, gradient hexane / ethyl acetate 0% to 20%) affording pure benzophenone **1a** as a white solid in 78% yield (70 mg, 0.38 mmol).

Procedures for the Synthesis of Ketones in Table 3:

All compounds in Table 3 were prepared according to the procedure detailed below. See the tabulated NMR data for any adjustments to the procedure employed or reaction temperature. For compounds **2k**, **2m**, and **2n**, 2,6-di-*tert*-butylpyridine (258 µL, 1.15 mmol) was also added together with the heterocycle to quench *in situ* generated triflic acid and prevent side reactions.

Representative Procedure: In a glove box, silver triflate (339 mg, 1.32 mmol) and iodine (126 mg, 0.49 mmol) were dry transferred into a 50 mL thick-walled glass reaction vessel sealable with a Teflon stopcock and equipped with a magnetic stir bar. $[Pd(allyl)Cl]_2$ (5 mg, 0.013 mmol) and the residual iodine left after dry transferring were dissolved in 2 mL of 1,2-dichloroethane and added to the reaction vessel. Benzene was added to the reaction vessel via micropipette (89 µL, 1.00 mmol). The vessel was sealed and the reaction mixture was allowed to stir at room temperature for 4.5 h in the glove box, upon which the vessel was opened and *tert*-butylbenzene was added to the reaction mixture via micropipette (232 µL, 1.50 mmol). The vessel was sealed, removed from the glove box, evacuated and backfilled three times with carbon monoxide, and finally pressurized with 4 atm carbon monoxide. The reaction mixture was stirred and heated at 60 °C for 22 h, upon which the pressure was released in a fume hood. The reaction mixture was

filtered through a pad of silica and the reaction vessel was thoroughly rinsed with $CHCl_3$ followed by ethyl acetate. The solvents were removed *in vacuo* and the product was purified via column chromatography (silica gel, gradient hexane / ethyl acetate 0% to 20%) affording pure ketone **2d** as a white solid in 92% yield (109 mg, 0.46 mmol).

Procedure for the Synthesis of Crystal Violet 3 (Scheme 2):

In a glove box, iodine (61 mg, 0.24 mmol) was dry transferred into a 50 mL thick-walled glass reaction vessel sealable with a Teflon stopcock and equipped with a magnetic stir bar. $[Pd(allyl)Cl]_2$ (2 mg, 0.0066 mmol) and the residual iodine left after dry transferring were dissolved in 1 mL of 1,2-dichloroethane and added to the reaction vessel. *N,N*-dimethylaniline was added to the reaction vessel via micropipette (193 µL, 1.52 mmol). The vessel was sealed, removed from the glove box, evacuated and backfilled three times with carbon monoxide, and finally pressurized with 4 atm carbon monoxide. The reaction mixture was stirred and heated at 100 °C for 22 h, after which the pressure was released in a fume hood. The reaction mixture was filtered with added 300 mL acetonitrile to remove solids. 1,3,5-trimethoxybenzene (13 mg, 0.079 mmol) was added to the reaction solution as an NMR standard, followed by 0.2 mL triethylamine to quench acid generated during the reaction. A 1 mL aliquot of the reaction mixture was taken from the solution and ^{1}H NMR analysis revealed the formation of **3** together with its demethylated isomer in 41% yield (ratio of 1:1.2 of R = Me : H).

For isolation of **3**, the procedure above was repeated on a larger scale [iodine (126 mg, 0.50 mmol), $[Pd(allyl)Cl]_2$ (5 mg, 0.012 mmol), and *N,N*-dimethylaniline (387 µL, 3.05 mmol) in 2 mL 1,2-dichloroethane]. Upon release of CO at the end of the reaction, the reaction mixture was filtered through a pad of basic alumina and the reaction vessel was thoroughly rinsed with acetonitrile. The solvents were removed *in vacuo* and the product was purified via column chromatography (basic alumina, gradient $CHCl_3$ / MeCN 0% to 30%). The products elute close together and care must be taken to very slowly increase the solvent gradient (some overlap in products is unavoidable) to afford pure Crystal Violet **3** (R = Me) as a green solid in 21% yield (52 mg, 0.10 mmol) and pure Methyl Violet 6B **3’** (R = H) as a green solid in 7% yield (16 mg, 0.034 mmol). Both of these products are sparingly soluble in many solvents (most notably prior

to removal of the acid), and require polar solvents such as acetonitrile or methanol for quantitative dissolution.

III. Mechanistic Studies

Monitoring Catalysis with ^{1}H NMR in Figure 2b:

In a glove box, silver triflate (34 mg, 0.13 mmol) and iodine (12 mg, 0.048 mmol) were dry transferred into a J. Young NMR tube. $[Pd(allyl)Cl]_2$ (1 mg, 0.0014 mmol), cyclooctane standard (1 mg, 0.012 mmol), and the residual iodine left after dry transferring were dissolved in 600 µL of DCE and added to the reaction vessel. To this mixture, benzene (13 µL, 0.15 mmol) was added. The J. Young NMR tube was sealed with a Teflon stopcock and removed from the glove box where it was then connected to a Schlenk line via a glass adaptor and placed in liquid nitrogen. The headspace of the NMR tube was evacuated and 5 atm of CO was condensed into the NMR tube. After two hours at room temperature a ^{1}H NMR experiment was taken showing 94% yield of iodobenzene. The J. Young NMR tube was subsequently heated at 60 °C for 42 h and ^{1}H and ^{13}C NMR analysis revealed the formation of benzophenone **1a** in 88% yield.

In situ iodobenzene: ^{1}H NMR (400 MHz, 1,2-dichloroethane): δ 7.70 (d, *J* = 7.4 Hz, 2H), 7.40 (t, obscured by benzene peak, 1H), 7.14 (t, *J* = 7.8 Hz, 2H). ^{13}C NMR (101 MHz, 1,2-dichloroethane): δ 136.7, 130.0, 127.4, 94.0.

Pure iodobenzene: ^{1}H NMR (400 MHz, 1,2-dichloroethane): δ 7.70 (d, *J* = 7.1 Hz, 2H), 7.33 (t, *J* = 7.5 Hz, 1H), 7.10 (t, *J* = 7.8 Hz, 2H). ^{13}C NMR (101 MHz, 1,2-dichloroethane): δ 136.9, 129.8, 127.0, 93.7.

In situ iodobenzene ^{1}H and ^{13}C NMR spectra:

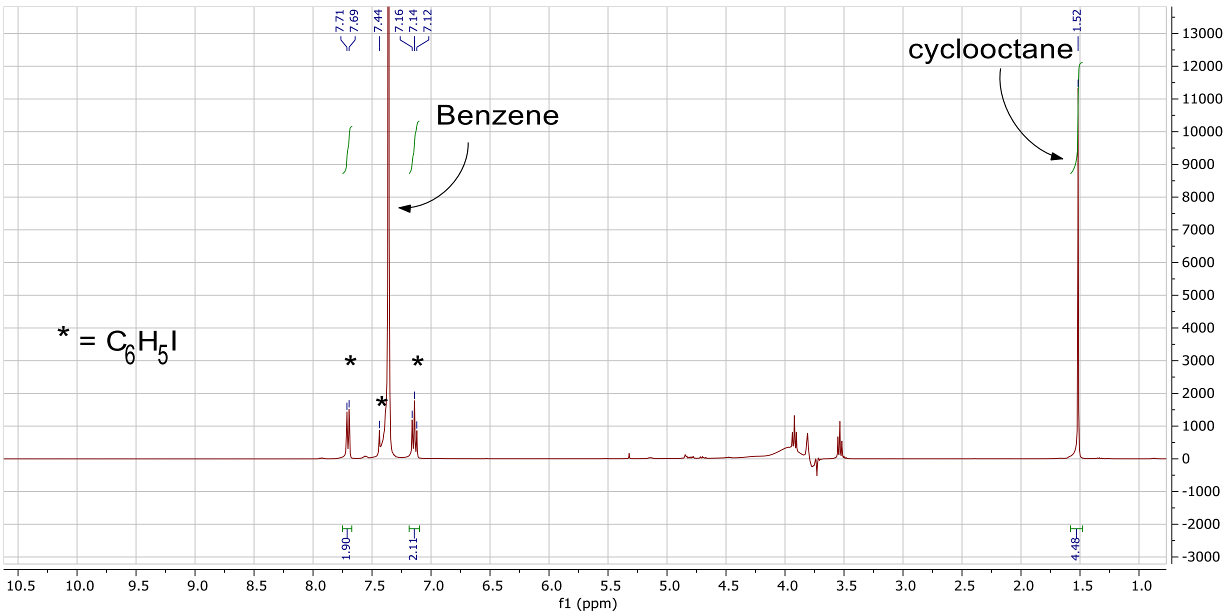

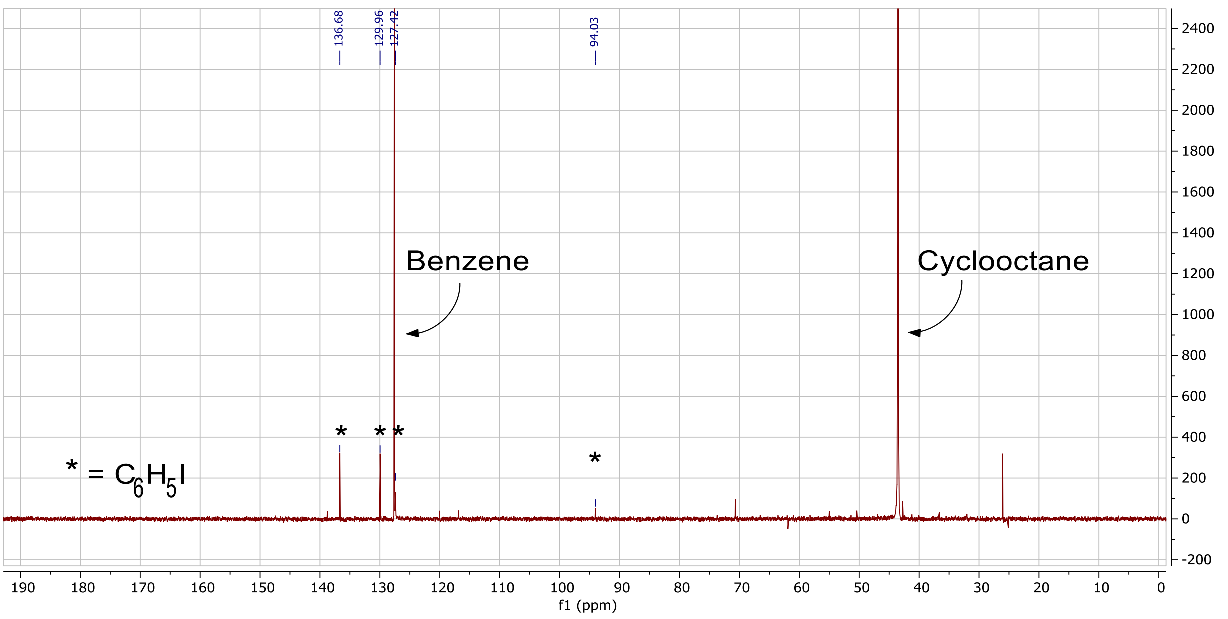

In situ Benzophenone ^{1}H and ^{13}C NMR spectra:

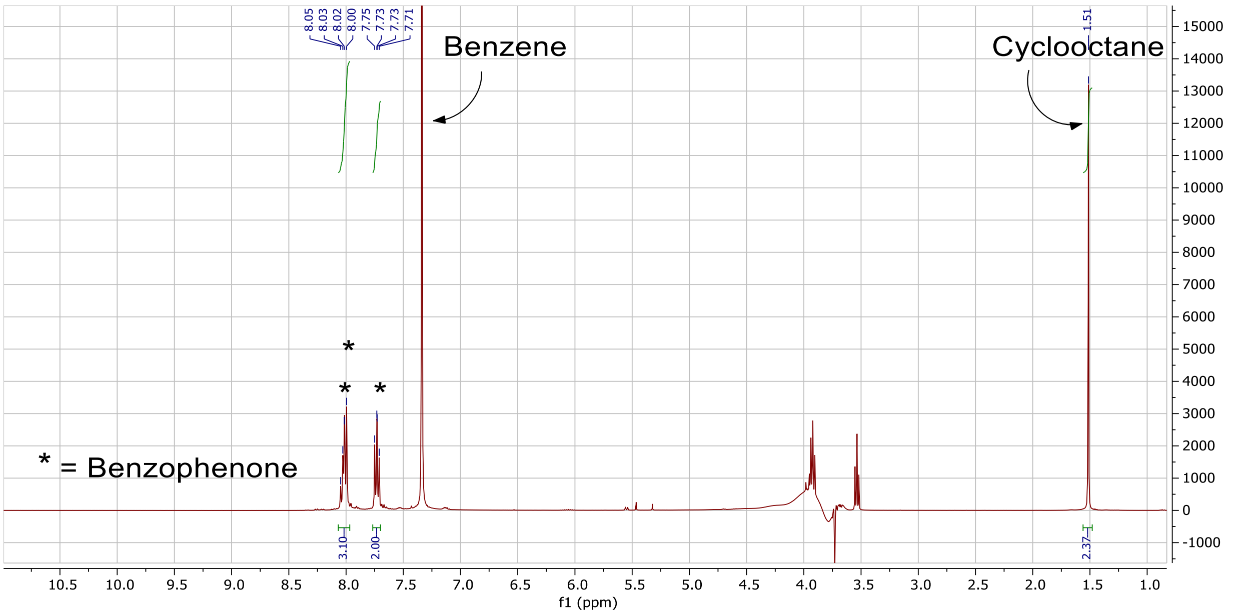

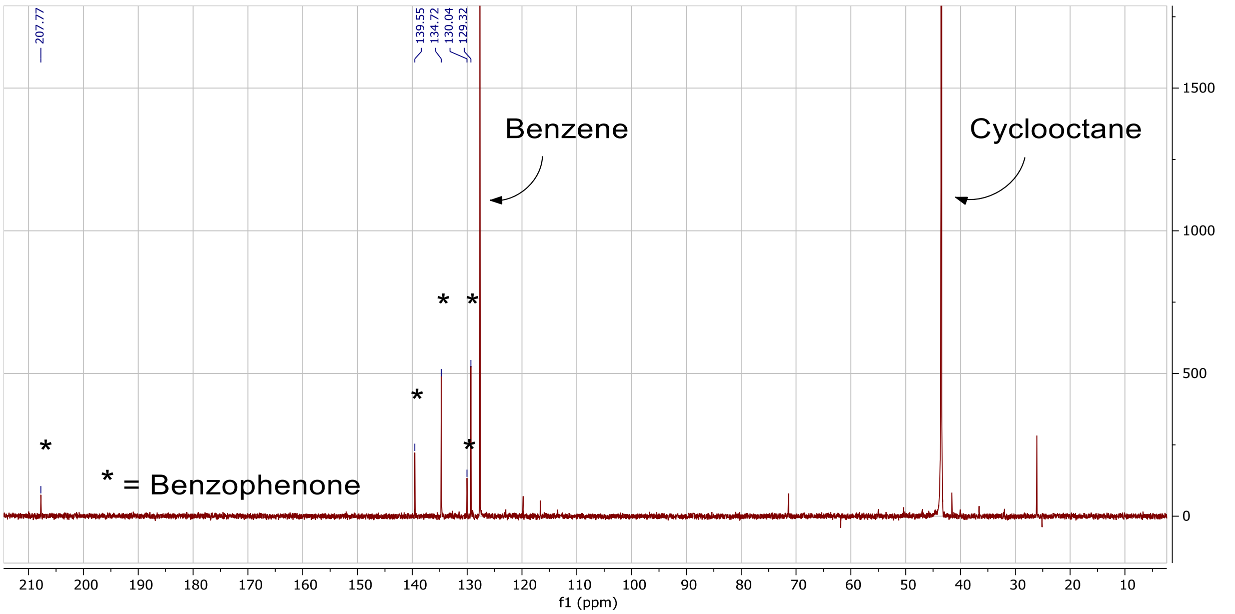

Reaction of *tert*-Butylbenzene, AgOTf, and I_2 (Figure 2c):

In a glove box, iodine (63 mg, 0.25 mmol) and cyclooctane standard (6 mg, 0.057 mmol) were added to a vial containing silver triflate (114 mg, 0.44 mmol) and a stir bar using 1 mL 1,2-dichloroethane. To this mixture, *tert*-butylbenzene (81 µL, 0.52 mmol) was added. The reaction mixture was stirred for 30 min at room temperature, after which the stirring was halted, and the

precipitate allowed to settle. 1 ml of this mixture was transferred to an NMR tube, and ^{1}H NMR analysis shows the formation of 4-*tert*-butyliodobenzene in >99% yield.

In situ 4-tert-butyliodobenzene: ^{1}H NMR (400 MHz, 1,2-dichloroethane): δ 7.59 (d, *J* = 8.6 Hz, 2H), 7.16 (d, *J* = 8.7 Hz, 2H), 1.26 (s, 9H).

Pure 4-tert-butyliodobenzene: ^{1}H NMR (400 MHz, 1,2-dichloroethane): 7.59 (d, *J* = 8.6 Hz, 2H), 7.13 (d, *J* = 8.6 Hz, 2H), 1.26 (s, 9H).

<u>*In Situ* 4-iodo-*tert*-butylbenzene ^{1}H NMR Spectrum:</u>

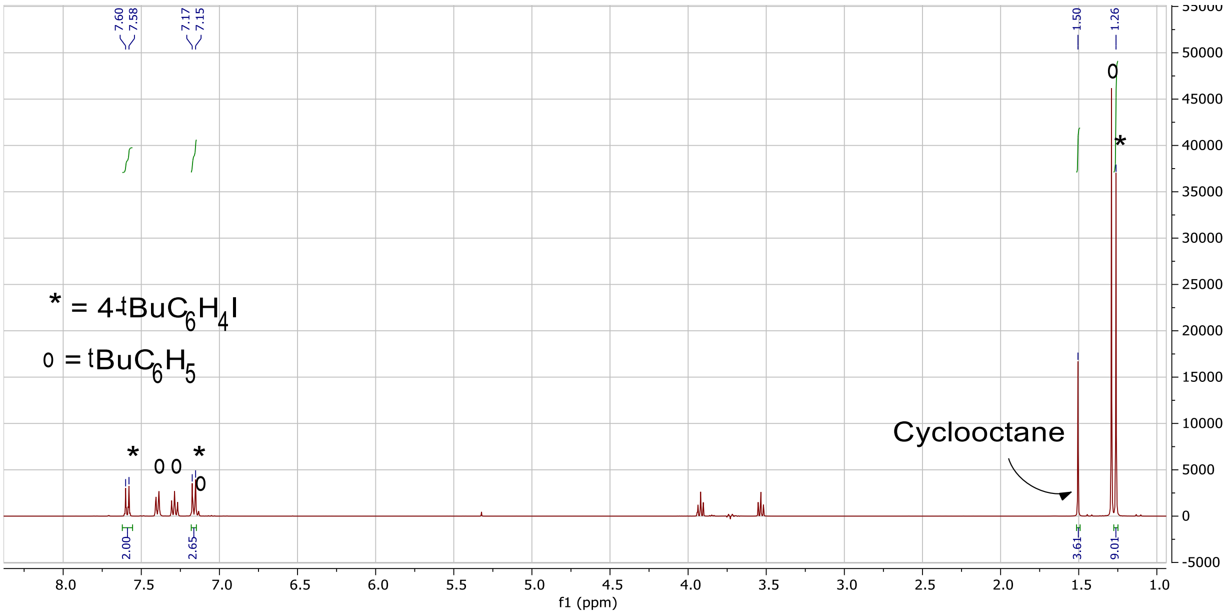

<u>Pd-Catalyzed Carbonylation of 4-Iodo-*tert*-butylbenzene and *tert*-Butylbenzene at Room Temperature (Figure 2c):</u>

In a glove box, silver triflate (118 mg, 0.46 mmol) was dry transferred into a 50 mL thick-walled glass reaction vessel sealable with a Teflon stopcock and equipped with a magnetic stir bar. $[Pd(allyl)Cl]_2$ (3 mg, 0.0071 mmol) and 4-*tert*-butyliodobenzene (65 mg, 0.25 mmol) were dissolved in 1 mL of 1,2-dichloroethane and added to the reaction vessel. *tert*-butylbenzene was added to the reaction vessel via micropipette (77 µL, 0.50 mmol). The vessel was sealed, removed from the glove box, evacuated and backfilled three times with carbon monoxide, and finally pressurized with 4 atm carbon monoxide. The reaction mixture was stirred at room temperature for 22 h and afterward the CO was removed on a Schlenk line before the vessel was

brought into the glove box. Cyclooctane standard (7 mg, 0.061 mmol) was then added to the reaction vessel. A 1 mL sample of the mixture was added to a J. Young NMR tube and ^{1}H NMR analysis revealed the formation of ketone **1b** in 23% yield.

Carbonylation of 4-Iodo-*tert*-butylbenzene at Room Temperature ^{1}H NMR Spectrum:

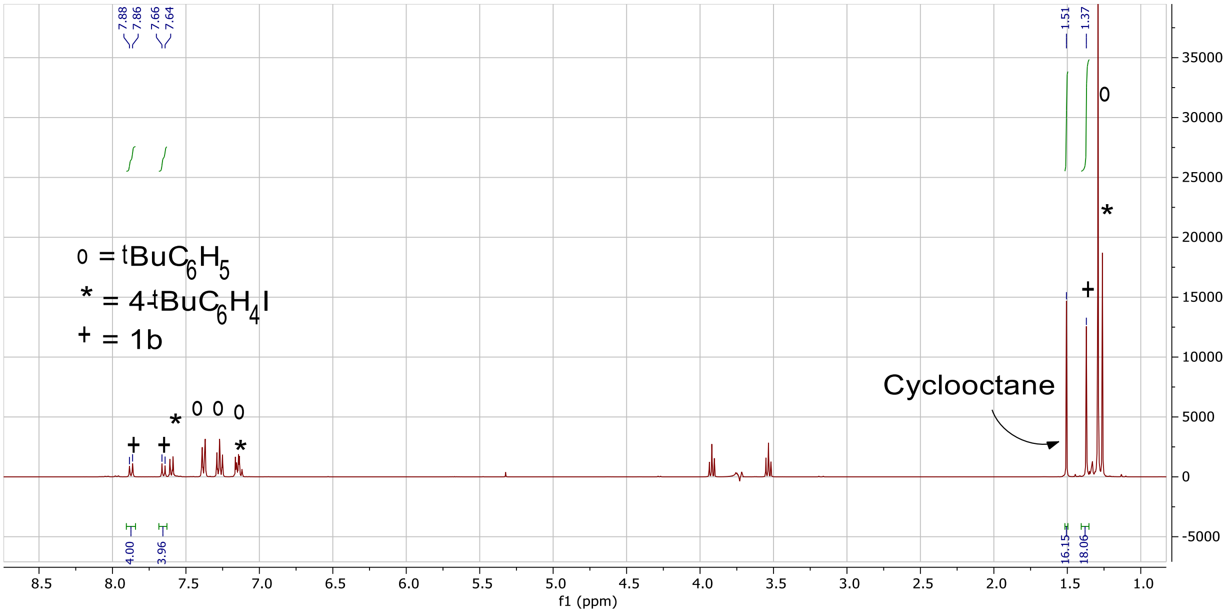

IV. Spectroscopic Data for Compounds 1

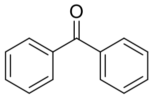

Benzophenone 1a.[1] Prepared according to the general procedure. White solid, 78% yield (70 mg, 0.38 mmol). ^{1}H NMR (500 MHz, $CDCl_3$): δ 7.81 (d, *J* = 7.7 Hz, 4H), 7.58 (t, *J* = 7.4 Hz, 2H), 7.48 (t, *J* = 7.6 Hz, 4H). ^{13}C NMR (126 MHz, $CDCl_3$): δ 196.8, 137.7, 132.5, 130.1, 128.4. NMR data corresponds with the previously reported compound.[1]

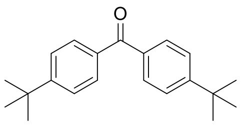

Bis(4-(*tert*-butyl)phenyl)methanone 1b.[1] Prepared according to the general procedure using *tert*-butylbenzene (232 µL, 1.50 mmol). White solid, 90% yield (132 mg, 0.45 mmol). ^{1}H NMR (500 MHz, $CDCl_3$): δ 7.79 (d, *J* = 8.5 Hz, 4H), 7.50 (d, *J* = 8.5 Hz, 4H), 1.38 (s, 18H). ^{13}C NMR (126 MHz, $CDCl_3$): δ 196.2, 155.9, 135.2, 130.1, 125.2, 35.1, 31.2. NMR data corresponds with the previously reported compound.[1]

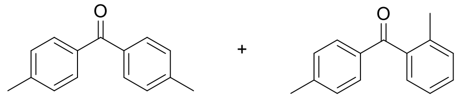

1.1 1

Di-*p*-tolylmethanone 1c and *o*-toylyl(*p*-tolyl)methanone 1c'.[1,2] Prepared according to the general procedure using toluene (160 µL, 1.50 mmol). White solid, 91% isolated total yield of both isomers (1.1:1 ratio) (94 mg, 0.45 mmol). ^{1}H NMR (500 MHz, $CDCl_3$): δ 7.71 (m, 6H, major and minor isomer), 7.37 (td, *J* = 8.1, 1.7 Hz, 1H, minor isomer), 7.32-7.22 (m, 9H, major and minor isomer), 2.42 (s, 6H, major isomer), 2.41 (s, 3H, minor isomer), 2.32 (s, 3H, minor isomer). ^{13}C NMR (126 MHz, $CDCl_3$): δ 198.3, 196.2, 144.1, 142.9, 139.0, 136.5, 135.2, 135.2, 130.9, 130.3, 130.2, 130.0, 129.2, 128.9, 128.3, 125.2, 21.7, 21.6, 19.9. NMR data corresponds with the previously reported compounds.[1,2]

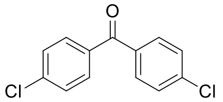

Bis(4-chlorophenyl)methanone 1d.[1] Prepared according to the general procedure using chlorobenzene (2 mL, 19.6 mmol) and 150 ^{o}C. White, crystalline solid, 88% yield (110 mg, 0.44 mmol). ^{1}H NMR (500 MHz, $CDCl_3$): δ 7.72 (d, *J* = 8.6 Hz, 4H), 7.46 (d, *J* = 8.6 Hz, 4H). ^{13}C NMR (126 MHz, $CDCl_3$): δ 194.3, 139.2, 135.6, 131.4, 128.9. HRMS: Calculated for $C_{13}H_8Cl_2ONa$ ($M+Na^+$): 272.9844, found: 272.9844. NMR data corresponds with the previously reported compound.[1]

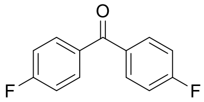

Bis(4-fluorophenyl)methanone 1e.[3] Prepared according to the general procedure using fluorobenzene as solvent (2 mL, 21.3 mmol) and at 150 °C. Yellow solid, 82% yield (90 mg, 0.41 mmol). ^{1}H NMR (500 MHz, $CDCl_3$): δ 7.81 (dd, $^{3}J_{HH}$ = 8.8 Hz, $^{4}J_{HF}$ = 5.4 Hz, 4H), 7.16 (dd, $^{3}J_{HH}$ = 8.6 Hz, $^{3}J_{HF}$ = 8.6 Hz, 4H). ^{13}C NMR (126 MHz, $CDCl_3$): δ 193.9, 165.5 (d, *J* = 254.2 Hz), 133.8 (d, *J* = 3.1 Hz), 132.6 (d, *J* = 9.1 Hz), 115.7 (d, *J* = 21.8 Hz). NMR data corresponds with the previously reported compound.[3]

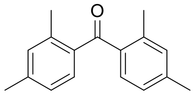

Bis(2,4-dimethylphenyl)methanone 1f.[3] Prepared according to the general procedure using 1,3-dimethylbenzene (184 µL, 1.50 mmol). Yellow oil, 65% yield (77 mg, 0.32 mmol). ^{1}H NMR (500 MHz, $CDCl_3$): δ 7.21 (d, *J* = 7.8 Hz, 2H), 7.09 (s, 2H), 6.99 (d, *J* = 7.7 Hz, 2H), 2.42 (s, 3H), 2.36 (s, 3H). ^{13}C NMR (126 MHz, $CDCl_3$): δ 200.5, 141.4,

138.3, 136.6, 132.3, 130.7, 126.0, 21.5, 20.7. NMR data corresponds with the previously reported compound.[3]

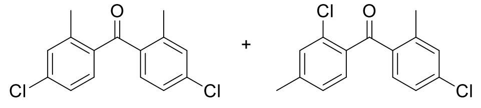

3 1

Bis(2-chloro-4-methylphenyl)methanone 1g. Prepared according to the general procedure using 1-chloro-3-methylbenzene (177 µL, 1.50 mmol) and at 100 °C. Colourless oil, 91% isolated total yield of both isomers (3:1 ratio) (127 mg, 0.45 mmol). Isomers were separated for characterization purposes using a preparation scale silica TLC plate with 3% ethyl acetate / hexanes. ^{1}H NMR (500 MHz, $CDCl_3$): δ 7.29 (br s, 2H), 7.24-7.17 (m, 4H), 2.41 (s, 6H). ^{13}C NMR (126 MHz, $CDCl_3$): δ 198.5, 140.5, 137.4, 137.0, 131.7, 131.7, 125.9, 20.7. HRMS: Calculated for $C_{15}H_{12}OCl_2Na$ (M+Na$^+$): 301.0157, found: 301.0153. **(4-chloro-2-methylphenyl)(2-chloro-4-methylphenyl)methanone 1g'.** ^{1}H NMR (500 MHz, $CDCl_3$): δ 7.35-7.31 (m, 1H), 7.29 (br s, 1H), 7.27-7.23 (m, 2H), 7.18-7.13 (m, 2H), 2.51 (s, 3H), 2.40 (s, 3H). ^{13}C NMR (126 MHz, $CDCl_3$): δ 196.5, 143.0, 141.3, 137.8, 136.3, 136.1, 132.4, 132.1, 131.8, 131.1, 130.4, 127.7, 125.8, 21.4, 21.0. HRMS: Calculated for $C_{15}H_{12}OCl_2Na$ (M+Na$^+$): 301.0157, found: 301.0148.

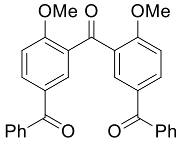

(Carbonylbis(4-methoxy-3,1-phenylene))bis(phenylmethanone) 1h. Prepared according to the general procedure using (4-methoxyphenyl)(phenyl)methanone (320 mg, 1.51 mmol). Yellow solid, 55% yield (122 mg, 0.27 mmol). ^{1}H NMR (500 MHz, $CDCl_3$): δ 8.04 (s, 2H), 7.98 (d, *J* = 8.3, 2H), 7.74 (d, *J* = 7.3 Hz, 4H), 7.54 (t, *J* = 7.1 Hz, 2H), 7.44 (t, *J* = 7.3 Hz, 4H), 6.99 (d, *J* = 8.6 Hz, 2H), 3.73 (s, 6H). ^{13}C NMR (126 MHz, $CDCl_3$): δ 194.9, 193.1, 161.6, 137.7, 135.4, 133.0, 132.3, 129.9, 129.7, 129.3, 128.4, 111.0, 56.0. HRMS: Calculated for $C_{29}H_{22}O_5Na$ (M+Na$^+$): 473.1359, found: 473.1362.

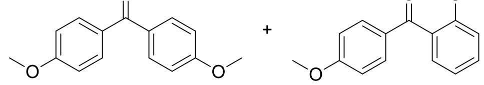

3.5 1

Bis(4-methoxyphenyl)methanone 1i and (2-methoxyphenyl)(4-methoxyphenyl)methanone 1i'.[1,4] Prepared according to the general procedure using anisole (163 µL, 1.50 mmol). White solid, 73% isolated total yield of both isomers (3.5:1 ratio) (88 mg,

0.36 mmol). ^{1}H NMR (500 MHz, $CDCl_3$): δ 7.78 (d, *J* = 8.7 Hz, 6H, both isomers), 7.43 (m, 1H, minor isomer), 7.31 (dd, *J* = 7.5, 1.8 Hz, 1H, minor isomer), 7.02 (t, *J* = 7.5 Hz, 1H, minor isomer), 6.95 (d, *J* = 8.8 Hz, 5H, both isomers), 6.90 (d, *J* = 8.8 Hz, 2H, minor isomer), 3.87 (s, 6H, major isomer), 3.85 (s, 3H, minor isomer), 3.73 (s, 3H, minor isomer). ^{13}C NMR (126 MHz, $CDCl_3$): δ 195.2, 194.5, 163.6, 162.9, 157.1, 132.4, 132.3, 131.5, 130.8, 130.8, 129.4, 129.3, 120.5, 113.6, 113.6, 111.5, 55.7, 55.6. NMR data corresponds with the previously reported compound.[1,4]

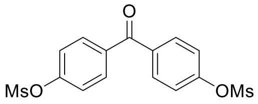

Carbonylbis(4,1-phenylene)dimethanesulfonate 1j. Prepared according to the general procedure using phenyl methanesulfonate (262 mg, 1.52 mmol) and at 100 °C. White solid, 62% yield (115 mg, 0.31 mmol). ^{1}H NMR (500 MHz, $CDCl_3$): δ 7.84 (d, *J* = 8.5 Hz, 4H), 7.40 (d, *J* = 8.5 Hz, 4H), 3.21 (s, 6H). ^{13}C NMR (126 MHz, $CDCl_3$): δ 193.6, 152.2, 135.9, 132.0, 122.1, 38.0. HRMS: Calculated for $C_{15}H_{15}O_7S_2$ (M+H$^+$): 371.02537, found: 371.02643.

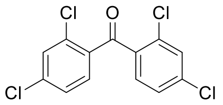

Bis(2,4-dichlorophenyl)methanone 1k. Prepared according to the general procedure using 1,3-dichlorobenzene as solvent (2 mL, 17.5 mmol) and 150 °C. Orange solid, 81% yield (127 mg, 0.40 mmol). ^{1}H NMR (500 MHz, $CDCl_3$): δ 7.47 (d, *J* = 8.3, 2H), 7.43 (d, *J* = 1.9 Hz, 2H), 7.33 (dd, *J* = 8.3, 1.9, 2H). ^{13}C NMR (126 MHz, $CDCl_3$): δ 192.3, 138.6, 136.2, 133.7, 131.9, 130.7, 127.6. HRMS: Calculated for $C_{13}H_6Cl_4ONa$ (M+Na$^+$): 340.9065, found: 340.9055.

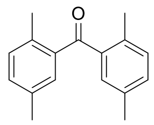

Bis(2,5-dimethylphenyl)methanone 1l.[1] Prepared according to the general procedure using 1,4-dimethylbenzene (185 µL, 1.50 mmol). White solid, 85% yield (100 mg, 0.42 mmol). ^{1}H NMR (500 MHz, $CDCl_3$): δ 7.20 (d, *J* = 7.8 Hz, 2H), 7.17 (d, *J* = 7.8 Hz, 2H), 7.14 (s, 2H), 2.40 (s, 6H), 2.31 (s, 6H). ^{13}C NMR (126 MHz, $CDCl_3$): δ 201.2, 139.1, 135.0, 135.0, 131.8, 131.4, 130.6, 20.9, 20.2. NMR data corresponds with the previously reported compound.[1]

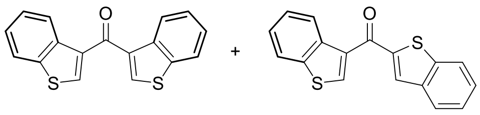

2.4 1

Bis(benzo[*b*]thiophen-3-yl)methanone 1m and benzo[*b*]thiophen-2-yl(benzo[*b*]thiophen-3-yl)methanone 1m'.[5,6] Prepared according to the general procedure using benzo[*b*]thiophene (222 mg, 1.65 mmol). Orange solid, 65% isolated total yield of both isomers (2.4:1 ratio) (96 mg, 0.32 mmol). ^{1}H NMR (500 MHz, $CDCl_3$): δ 8.60 (d, *J* = 8.1 Hz, 2H major isomer), 8.52 (d, *J* = 8.1 Hz, 2H minor isomer), 8.25 (s, 1H minor isomer), 8.05 (s, 2H major isomer), 7.95 (s, 1H minor isomer), 7.94-7.85 (m, 2H for the major isomer, 3H for the minor isomer), 7.57-7.29 (m, 4H for both isomers). ^{13}C NMR (126 MHz, $CDCl_3$): δ 184.9, 183.3, 144.2, 142.5, 140.2, 140.0, 139.0, 137.3, 137.2, 136.9, 136.4, 134.6, 130.9, 127.4, 126.1, 125.8, 125.7, 125.7, 125.2, 125.0, 124.9, 122.9, 122.5, 122.4. NMR data corresponds with the previously reported compounds.[5,6]

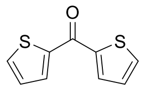

Di(thiophen-2-yl)methanone 1n.[7] Prepared according to the general procedure using thiophene (120 µL, 150 mmol). Yellow oil, 48% yield (46 mg, 0.24 mmol). ^{1}H NMR (500 MHz, $CDCl_3$): δ 7.89 (dd, *J* = 3.8, 1.2 Hz, 2H), 7.69 (dd, *J* = 5.0, 1.2 Hz, 2H), 7.18 (dd, *J* = 5.0, 3.8 Hz, 2H). ^{13}C NMR (126 MHz, $CDCl_3$): δ 178.9, 143.0, 133.6, 133.3, 128.1. HRMS: Calculated for $C_9H_6OS_2Na$ ($M+Na^+$): 216.9752, found: 216.9749. NMR data corresponds with the previously reported compound.[7]

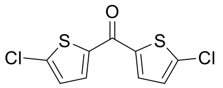

Bis(5-chlorothiophen-2-yl)methanone 1o. Prepared according to the general procedure using 2-chlorothiophene (138 µL, 1.50 mmol).Yellow solid, 55% yield (72 mg, 0.27 mmol). ^{1}H NMR (400 MHz, $CDCl_3$): δ 7.64 (d, *J* = 4.1 Hz, 2H), 7.01 (d, *J* = 4.1 Hz, 2H). ^{13}C NMR (101 MHz, $CDCl_3$): δ 176.5, 140.7, 139.7, 132.6, 127.6. HRMS: Calculated for $C_9H_4OS_2Cl_2Na$ ($M+Na^+$): 284.8973, found: 284.8964.

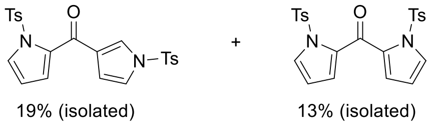

19% (isolated) 13% (isolated)

(1-tosyl-1*H*-pyrrol-2-yl)(1-tosyl-1*H*-pyrrol-3-yl)methanone 1p. Prepared according to the general procedure using 1-tosyl-1*H*-pyrrole (334 mg, 1.51 mmol). Orange oil, 19% yield (44 mg, 0.095 mmol). ^{1}H NMR (500 MHz, $CDCl_3$): δ 7.96 (d, *J* = 8.5 Hz,

2H), 7.77 (d, *J* = 8.4 Hz, 2H), 7.71 (dd, *J* = 3.2, 1.7 Hz, 1H), 7.65 (t, *J* = 1.9 Hz, 1H), 7.36-7.29 (m, 4H), 7.14 (dd, *J* = 3.3, 2.2 Hz, 1H), 6.85 (dd, *J* = 3.7, 1.7 Hz, 1H), 6.70 (dd, *J* = 3.3, 1.6 Hz, 1H), 6.33 (t, *J* = 3.4 Hz, 1H), 2.41 (s, 6H). ^{13}C NMR (126 MHz, $CDCl_3$): δ 176.1, 146.2, 145.1, 136.4, 135.0, 133.3, 130.5, 129.6, 129.3, 128.4, 128.4, 127.4, 125.8, 123.6, 121.5, 113.6, 110.8, 21.8. HRMS: Calculated for $C_{23}H_{21}O_5N_2S_2$ (M+H$^+$): 469.08864, found: 469.09030. **Bis(1-tosyl-1*H*-pyrrol-2-yl)methanone 1p'.** Orange solid, 13% yield (30 mg, 0.065 mmol). ^{1}H NMR (500 MHz, $CDCl_3$): δ 7.84 (d, *J* = 8.4 Hz, 4H), 7.69 (dd, *J* = 3.2, 1.7 Hz, 2H), 7.34 (d, *J* = 8.2 Hz, 4H), 6.80 (dd, *J* = 3.7, 1.8 Hz, 2H), 6.29 (t, *J* = 3.4 Hz, 2H), 2.47 (s, 6H). ^{13}C NMR (126 MHz, $CDCl_3$): δ 172.2, 145.0, 136.2, 133.3, 129.4, 129.2, 128.9, 124.6, 110.7, 21.9. HRMS: Calculated for $C_{23}H_{21}O_5N_2S_2$ (M+H$^+$): 469.08864, found: 469.09031.

V. Spectroscopic Data for Compounds 2

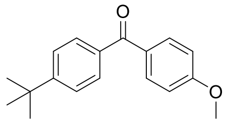

(4-(*tert*-butyl)phenyl)(4-methoxyphenyl)methanone 2a.[8] Prepared according to the general procedure and allowing *tert*-butylbenzene (155 µL, 1.00 mmol) to iodinate for 4.5 h before addition of anisole (163 µL, 1.50 mmol). White solid, 72% yield (95 mg, 0.36 mmol). ^{1}H NMR (500 MHz, $CDCl_3$): δ 7.83 (d, *J* = 8.9 Hz, 2H), 7.72 (d, *J* = 8.5 Hz, 2H), 7.48 (d, *J* = 8.5 Hz, 2H), 6.95 (d, *J* = 8.9 Hz, 2H), 3.86 (s, 3H), 1.36 (s, 9H). ^{13}C NMR (126 MHz, $CDCl_3$): δ 195.3, 163.1, 155.6, 135.5, 132.5, 130.5, 129.8, 125.2, 113.5, 55.5, 35.1, 31.2. NMR data corresponds with the previously reported compound.[8]

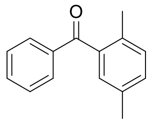

(2,5-dimethylphenyl)(phenyl)methanone 2b.[9] Prepared according to the general procedure and allowing benzene (89 µL, 1.00 mmol) to iodinate for 4 h before addition of 1,4-dimethylbenzene (185 µL, 1.50 mmol). Orange oil, 99% yield (104 mg, 0.50 mmol). ^{1}H NMR (500 MHz, $CDCl_3$): δ 7.82 (d, *J* = 7.1 Hz, 2H), 7.58 (t, *J* = 7.4 Hz, 1H), 7.46 (t, *J* = 7.7 Hz, 2H), 7.23-7.15 (m, 2H), 7.13 (s, 1H), 2.34 (s, 3H), 2.28 (s, 3H). ^{13}C NMR (126 MHz, $CDCl_3$): δ 198.9, 138.7, 137.9, 134.8, 133.5, 133.1, 131.0, 130.9, 130.1, 129.0, 128.5, 20.9, 19.5. NMR data corresponds with the previously reported compound.[9]

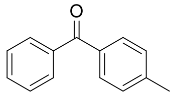

Phenyl(*p*-tolyl)methanone 2c.[9] Prepared according to the general procedure and allowing benzene (89 µL, 1.00 mmol) to iodinate for 4 h before addition of toluene (160 µL, 1.50 mmol). Pale-yellow oil, 68% yield (66 mg, 0.33 mmol). ^{1}H NMR (500 MHz, $CDCl_3$): δ 7.79 (d, *J* = 7.0 Hz, 2H), 7.73 (d, *J* = 8.1 Hz, 2H), 7.57 (t, *J* = 7.4 Hz, 1H), 7.47 (t, *J* = 7.7 Hz, 2H), 7.28 (d, *J* = 7.9 Hz, 2H), 2.44 (s, 3H). ^{13}C NMR (126 MHz, $CDCl_3$): δ 196.5, 143.3, 138.0, 134.9, 132.2, 130.4, 130.0, 129.0, 128.3, 21.7. NMR data corresponds with the previously reported compound.[9]

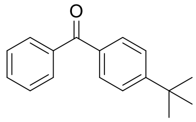

(4-(*tert*-butyl)phenyl)(phenyl)methanone 2d.[9] Prepared according to the general procedure. Yellow oil, 92% yield (109 mg, 0.46 mmol). ^{1}H NMR (500 MHz, $CDCl_3$): δ 7.81 (d, *J* = 7.1 Hz, 2H), 7.78 (d, *J* = 8.4 Hz, 2H), 7.57 (t, *J* = 7.4 Hz, 1H), 7.50 (d, *J* = 8.5 Hz, 2H), 7.47 (t, *J* = 7.7 Hz, 2H), 1.37 (s, 9H). ^{13}C NMR (126 MHz, $CDCl_3$): δ 196.4, 156.2, 138.0, 134.9, 132.2, 130.2, 130.0, 128.2, 125.3, 35.1, 31.2. NMR data corresponds with the previously reported compound.[9]

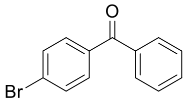

(4-bromophenyl)(phenyl)methanone 2e.[10] Prepared according to the general procedure and allowing bromobenzene (105 µL, 1.00 mmol) to iodinate at 100 °C for 6 h before addition of benzene (134 µL, 1.50 mmol). The carbonylation step also proceeded at 100 °C. Off-white solid, 54% yield (70 mg, 0.27 mmol). ^{1}H NMR (500 MHz, $CDCl_3$): δ 7.77 (d, *J* = 7.5 Hz, 2H), 7.73-7.56 (m, 5H), 7.48 (t, *J* = 7.6 Hz, 2H). ^{13}C NMR (126 MHz, $CDCl_3$): δ 195.7, 137.2, 136.4, 132.8, 131.7, 131.6, 130.0, 128.5, 127.6. NMR data corresponds with the previously reported compound.[10]

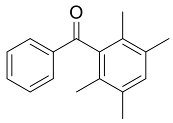

Phenyl(2,3,5,6-tetramethylphenyl)methanone 2f.[11] Prepared according to the general procedure and allowing benzene (89 µL, 1.00 mmol) to iodinate for 5.5 h before addition of 1,2,4,5-tetramethylbenzene (222 mg, 1.66). White Solid, 99% yield (118 mg, 0.50 mmol). ^{1}H NMR (500 MHz, $CDCl_3$): δ 7.84 (d, *J* = 7.5 Hz, 2H), 7.58 (t, *J* = 7.3 Hz, 1H), 7.45 (t, *J* = 7.7 Hz, 2H), 7.05 (s, 1H), 2.25 (s, 6H), 2.99 (s, 6H). ^{13}C NMR (126

MHz, $CDCl_3$): δ 201.7, 140.0, 137.5, 134.3, 133.6, 131.9, 129.8, 129.6, 128.9, 19.6, 16.4. NMR data corresponds with the previously reported compound.[11]

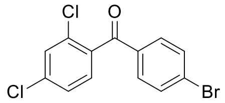

(4-bromophenyl)(2,4-dichlorophenyl)methanone 2g. Prepared according to the general procedure and allowing 1,3-dichlorobenzene (114 µL, 1.00 mmol) to iodinate at 100 °C for 22 h before addition of bromobenzene (526 µL, 5.00 mmol). The carbonylation step proceeded at 150 °C. Brown oil, 36% yield (60 mg, 0.18 mmol). ^{1}H NMR (500 MHz, $CDCl_3$): δ 7.67-7.60 (m, 4H), 7.49 (d, J = 1.8 Hz, 1H), 7.38 (dd, J = 8.2, 1.8 Hz, 1H), 7.33 (d, J = 8.2 Hz, 1H). ^{13}C NMR (126 MHz, $CDCl_3$): δ 193.4, 137.1, 136.5, 135.2, 132.5, 132.2, 131.5, 130.3, 130.2, 129.5, 127.4. HRMS: Calculated for $C_{13}H_7OBrCl_2Na$ ($M+Na^+$): 350.8950, found: 350.8943.

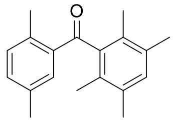

(2,5-dimethylphenyl)(2,3,5,6-tetramethylphenyl)methanone 2h. Prepared according to the general procedure and allowing 1,4-dimethylbenzene (123 µL, 1.00 mmol) to iodinate for 4 h before addition of 1,2,4,5-tetramethylbenzene (235 mg, 1.75 mmol). Off-white solid, 95% yield (126 mg, 0.47 mmol). ^{1}H NMR (500 MHz, $CDCl_3$): δ 7.25-7.11 (m, 3H), 7.02 (s, 1H), 2.68 (s, 3H), 2.25 (s, 9H), 2.00 (s, 6H). ^{13}C NMR (126 MHz, $CDCl_3$): δ 203.8, 141.9, 137.2, 136.7, 135.5, 134.2, 133.2, 132.8, 132.3, 131.7, 129.7, 21.8, 20.9, 19.6, 16.2. HRMS: Calculated for $C_{19}H_{22}ONa$ ($M+Na^+$): 289.1563, found: 289.1564.

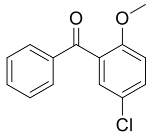

(5-chloro-2-methoxyphenyl)(phenyl)methanone 2i. Prepared according to the general procedure and allowing benzene (89 µL, 1.00 mmol) to iodinate for 5 h before addition of 4-chloroanisole (184 µL, 1.50 mmol). White solid, 85% yield (104 mg, 0.42 mmol). ^{1}H NMR (500 MHz, $CDCl_3$): δ 7.80 (d, J = 7.1 Hz, 2H), 7.57 (t, J = 7.4 Hz, 1H), 7.44 (t, J = 7.9 Hz, 2H), 7.41 (dd, J = 8.7, 2.7 Hz, 1H), 7.32 (d, J = 2.6 Hz, 1H), 6.93 (d, J = 8.9 Hz, 1H), 3.70 (s, 3H). ^{13}C NMR (126 MHz, $CDCl_3$): δ 194.9, 156.0, 137.3, 133.4, 131.5, 130.3, 129.9, 129.2, 128.5, 125.8, 113.0, 56.1. HRMS: Calculated for $C_{14}H_{11}O_2ClNa$ ($M+Na^+$): 269.0340, found: 269.0339.

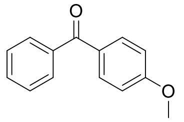

(4-methoxyphenyl)(phenyl)methanone 2j. Prepared according to the general procedure and allowing benzene (89 µL, 1.00 mmol) to iodinate for 4.5 h before addition of anisole (163 µL, 1.50 mmol). Pale-yellow oil, 90% yield (95 mg, 0.45 mmol). ^{1}H NMR (500 MHz, $CDCl_3$): δ 7.82 (d, *J* = 8.7 Hz, 2H), 7.74 (d, *J* = 7.3 Hz, 2H), 7.54 (t, *J* = 7.3 Hz, 1H), 7.45 (t, *J* = 7.5 Hz, 2H), 6.94 (d, *J* = 8.7 Hz, 2H), 3.85 (s, 3H). ^{13}C NMR (126 MHz, $CDCl_3$): δ 195.5, 163.2, 138.3, 132.6, 131.9, 130.1, 129.7, 128.2, 113.6, 55.5. NMR data corresponds with the previously reported compound.[9]

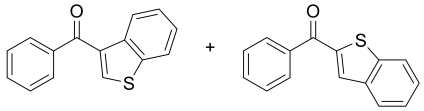

2.3 1

Benzo[*b*]thiophen-3-yl(phenyl)methanone 2k and Benzo[*b*]thiophen-2-yl(phenyl)methanone 2k'.[12] Prepared according to the general procedure and allowing benzene (89 µL, 1.00 mmol) to iodinate for 5.5 h before addition of benzo[*b*]thiophene (206 mg, 1.54 mmol). Orange oil, 59% isolated total yield of both isomers (2.3:1 ratio) (71 mg, 0.30 mmol). ^{1}H NMR (500 MHz, $CDCl_3$): δ 8.60 (d, *J* = 8.2 Hz, 1H, major isomer), 7.99 (s, 1H, major isomer), 7.95-7.81 (m, 5H for minor isomer, 3H for major isomer), 7.64-7.57 (m, 1H for both isomers), 7.57-7.38 (m, 4H for both isomers). ^{13}C NMR (126 MHz, $CDCl_3$): δ 190.9, 189.7, 143.2, 142.7, 140.1, 139.3, 139.1, 138.4, 137.9, 137.5, 134.8, 132.6, 132.4, 132.3, 129.6, 129.3, 128.6, 128.5, 127.5, 126.1, 125.7, 125.6, 125.2, 125.1, 123.0, 122.4. NMR data corresponds with the previously reported compound.[12]

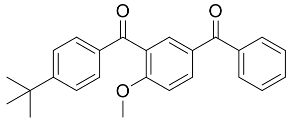

(5-benzoyl-2-methoxyphenyl)(4-(*tert*-butyl)phenyl)methanone 2l. Prepared according to the general procedure and allowing *tert*-butylbenzene (155 µL, 1.00 mmol) to iodinate for 4.5 h before addition of 4-methoxybenzophenone (322 mg, 1.52 mmol). Orange oil, 79% yield (147 mg, 0.39 mmol). ^{1}H NMR (500 MHz, $CDCl_3$): δ 8.00 (dd, *J* = 8.7, 2.3 Hz, 1H), 7.82 (d, *J* = 2.3 Hz, 1H), 7.9-7.73 (m, 4H), 7.55 (t, *J* = 7.4 Hz, 1H), 7.49-7.41 (m, 4H), 7.09 (d, *J* = 8.7 Hz, 1H), 3.84 (s, 3H), 1.33 (s, 9H). ^{13}C NMR (126 MHz, $CDCl_3$): δ 195.0, 194.9, 160.6, 157.3, 137.7, 134.5, 134.2, 132.2, 131.6, 130.0, 129.9, 129.8, 129.0, 128.4, 125.5, 111.0, 56.0, 35.2, 31.1. HRMS: Calculated for $C_{25}H_{25}O_3$ $(M+H^+)$: 373.17982, found: 373.18112.

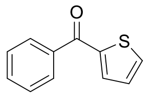

Phenyl(thiophen-2-yl)methanone 2m.[13] Prepared according to the general procedure and allowing benzene (89 µL, 1.00 mmol) to iodinate for 4.5 h before addition of thiophene (120 µL, 1.50 mmol). Yellow oil, 51% yield (47 mg, 0.25 mmol). ^{1}H NMR (500 MHz, $CDCl_3$): δ 7.87 (d, *J* = 7.1 Hz, 2H), 7.72 (dd, *J* = 5.0, 1.2 Hz, 1H), 7.65 (dd, *J* = 3.8, 1.2 Hz, 1H), 7.59 (t, *J* = 7.4 Hz, 1H), 7.50 (t, *J* = 7.6 Hz, 2H), 7.16 (dd, *J* = 4.8, 3.9 Hz, 1H). ^{13}C NMR (126 MHz, $CDCl_3$): δ 188.3, 143.8, 138.3, 135.0, 134.3, 132.4, 129.3, 128.5, 128.1. NMR data corresponds with the previously reported compound.[13]

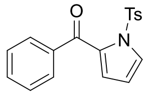

Phenyl(1-tosyl-1H-pyrrol-2-yl)methanone 2n. Prepared according to the general procedure using 3 mL 1,2-dichloroethane and allowing benzene (89 µL, 1.00 mmol) to iodinate for 5 h before addition of 1-tosyl-1H-pyrrole (332 mg, 1.50 mmol). Yellow solid, 39% yield (64 mg, 0.20 mmol). ^{1}H NMR (500 MHz, $CDCl_3$): δ 8.02 (d, *J* = 8.4 Hz, 2H), 7.80 (d, *J* = 6.9 Hz, 2H), 7.78 (dd, *J* = 3.2, 1.7 Hz, 1H), 7.55 (t, *J* = 7.4 Hz, 1H), 7.43 (t, *J* = 7.7 Hz, 2H), 7.36 (d, *J* = 8.1 Hz, 2H), 6.71 (dd, *J* = 3.7 ,1.7 Hz, 1H), 6.34 (t, *J* = 3.4 Hz, 1H), 2.43 (s, 3H). ^{13}C NMR (126 MHz, $CDCl_3$): δ 184.6, 145.1, 138.0, 136.3, 133.1, 132.8, 129.8, 129.6, 128.5, 128.3, 125.3, 110.7, 21.8. HRMS: Calculated for $C_{18}H_{15}NO_3SNa$ ($M+Na^+$): 348.0665, found: 348.0671.

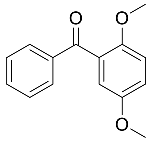

(2,5-dimethoxyphenyl)(phenyl)methanone 2o.[9] Prepared according to the general procedure and allowing benzene (89 µL, 1.00 mmol) to iodinate for 4 h before addition of 1,4-dimethoxybenzene (225 mg, 1.63 mmol). Orange oil, 55% yield (66 mg, 0.27 mmol). ^{1}H NMR (500 MHz, $CDCl_3$): δ 7.82 (d, *J* = 7.1 Hz, 2H), 7.54 (t, *J* = 7.4 Hz, 1H), 7.42 (t, *J* = 7.7 Hz, 2H), 7.00 (dd, *J* = 9.0, 3.1 Hz, 1H), 6.94-6.90 (m, 2H), 3.77 (s, 3H), 3.65 (s, 3H). ^{13}C NMR (126 MHz, $CDCl_3$): δ 196.2, 153.5, 151.5, 137.7, 133.1, 129.9, 129.5, 128.3, 117.4, 114.5, 113.1, 56.4, 55.9. NMR data corresponds with the previously reported compound.[9]

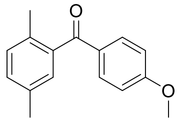

(2,5-dimethylphenyl)(4-methoxyphenyl)methanone 2p.[11] Prepared according to the general procedure allowing 1,4-dimethylbenzene (123 µL, 1.00 mmol) to

iodinate for 4.5 h before addition of anisole (163 μL, 1.50 mmol). Yellow oil, 82% yield (97 mg, 0.40 mmol). ^{1}H NMR (500 MHz, $CDCl_3$): δ 7.79 (d, *J* = 8.9 Hz, 2H), 7.19-7.12 (m, 2H), 7.09 (s, 1H), 6.92 (d, *J* = 8.9 Hz, 2H), 3.86 (s, 3H), 2.33 (s, 3H), 2.24 (s, 3H). ^{13}C NMR (126 MHz, $CDCl_3$): δ 197.7, 163.7, 139.2, 134.8, 132.9, 132.5, 130.8, 130.6, 128.4, 113.8, 55.5, 20.9, 19.4. NMR data corresponds with the previously reported compound.[11]

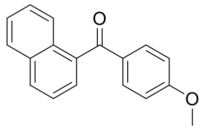

(4-methoxyphenyl)(naphthalen-1-yl)methanone 2q.[14] Prepared according to the general procedure and allowing naphthalene (129 mg, 1.01 mmol) to iodinate for 5.5 h before addition of anisole (163 μL, 1.50 mmol). Orange oil, 72% yield (95 mg, 0.36 mmol). ^{1}H NMR (500 MHz, $CDCl_3$): δ 8.03 (d, *J* = 8.3 Hz, 1H), 7.98 (d, *J* = 8.1 Hz, 1H), 7.91 (d, *J* = 7.8 Hz, 1H), 7.87 (d, *J* = 8.8 Hz, 2H), 7.60-7.45 (m, 4H), 6.92 (d, *J* = 8.9 Hz, 2H), 3.85 (s, 3H). ^{13}C NMR (126 MHz, $CDCl_3$): δ 196.8, 163.9, 137.1, 133.7, 132.8, 131.1, 130.9, 130.7, 128.4, 127.1, 126.9, 126.4, 125.8, 124.5, 113.8, 55.6. NMR data corresponds with the previously reported compound.[14]

VI. Spectroscopic Data for Compounds 3

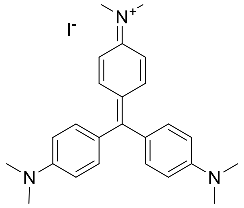

Crystal Violet 3 (R = Me).[15] Prepared according to the general procedure. Green solid in 21% yield (52 mg, 0.10 mmol). ^{1}H NMR (500 MHz, $CDCl_3$): δ 7.29 (d, *J* = 9.1 Hz, 6H), 6.82 (d, *J* = 9.2 Hz, 6H), 3.24 (s, 18H). ^{13}C NMR (126 MHz, $CDCl_3$): δ 178.3, 155.6, 139.8, 126.7, 112.5, 40.8. HRMS: Calculated for $C_{25}H_{30}N_3^+$ (M^+): 372.2434, found: 372.2437. NMR data corresponds with the previously reported compound.[15]

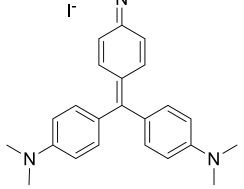

Methyl Violet 6B 3' (R = H). Prepared according to the general procedure. Green solid in 7% yield (16 mg, 0.034 mmol). ^{1}H NMR (500 MHz, $CDCl_3$): δ 8.14 (br s, 1H), 7.28 (d, *J* = 9.1 Hz, 4H), 7.24 (d, *J* = 9.0 Hz, 2H), 7.01 (br s, 2H), 6.79 (d, *J* = 9.1 Hz, 4H), 3.22 (s, 12H), 3.04 (d, *J* = 5.2 Hz, 3H). ^{13}C

NMR (126 MHz, $CDCl_3$): δ 177.8, 157.9, 155.1, 140.7, 139.3, 127.0, 126.8, 112.1, 111.8, 40.6, 29.8. HRMS: Calculated for $C_{24}H_{28}N_3^+$ (M^+): 358.2278, found: 358.2290.

VII. References

1. X. Wang, F.-D. Liu, H.-Y. Tu, A.-D. Zhang, *J. Org. Chem.* **2014**, *79*, 6554-6562.
2. J. Liu, X. Zhou, H. Rao, F. Xiao, C.-J. Li, G.-J. Deng, *Chem. Eur. J.* **2011**, *17*, 7996-7999.
3. L. Yang, T. Zeng, Q. Shuai, X. Guo, C.-J. Li, *Chem. Commun.* **2011**, *47*, 2161-2163.
4. S. Kaoru, K. Hideo, M. Teruaki, *Bull. Chem. Soc. Jpn.* **1993**, *66*, 3729-3734.
5. Y. Jiang, T. Kusakabe, K. Takahashi, K. Kato, *Org. Biomol. Chem.* **2014**, *12*, 3380-3385.
6. J. U. Sim, H. S. Son, J. H. Lee, H. C. Park, C. J. Lee, J. Y. Shin, Y. M. Baek, Dithienopyridine derivatives as electroluminescent hosts for an organic electroluminescent device. Repub. Korean Kongkae Taeho Kongbo KR 2015004493, 2015.
7. K. Kobayashi, Y. Nishimura, F. Gao, K. Gotoh, Y. Nishihara, K. Takagi, *J. Org. Chem.*, **2011**, *76*, 1949-1952.
8. K. Ito, H. Tamashima, N. Iwasawa, H. Kusama, *J. Am. Chem. Soc.* **2011**, *133*, 3716-3719.
9. G. Pandey, S. K. Tiwari, B. Singh, K. Vanka, S. Jain, *Chem. Commun.* **2017**, *53*, 12337-12340.
10. A. Gonzalez-de-Castro, J. Xiao, *J. Am. Chem. Soc.* **2015**, *137*, 8206-8218.
11. R. G. Kinney, J. Tjutrins, G. M. Torres, N. J. Liu, O. Kulkarni, B. A. Arndtsen, *Nat. Chem.* **2018**, *10*, 193-199.
12. R. G. Kinney, B. A. Arndtsen, *Angew. Chem. Int. Ed.* **2019**, *58*, 5085-5089; *Angew. Chem.* **2019**, *131*, 5139-5143.
13. S. Shi, M. Szostak, *Chem. Eur. J.* **2016**, *22*, 10420-10424.
14. Y. Kuang, Y. Wang, *Eur. J. Org. Chem.* **2014**, *2014*, 1163-1166.
15. P. Kaur, D. Sareen, K. Singh, *Dalton Trans.* **2012**, *41*, 9607-9610.

VIII. NMR Spectra

^{1}H NMR and ^{13}C NMR of 1a

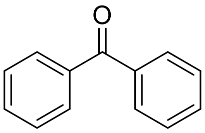

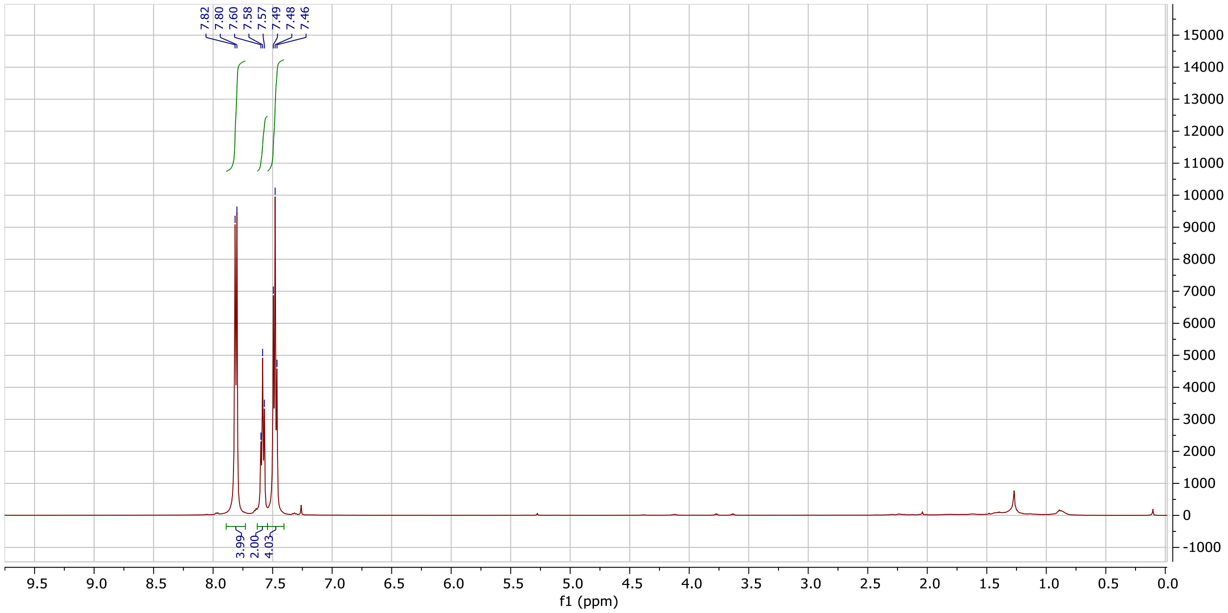

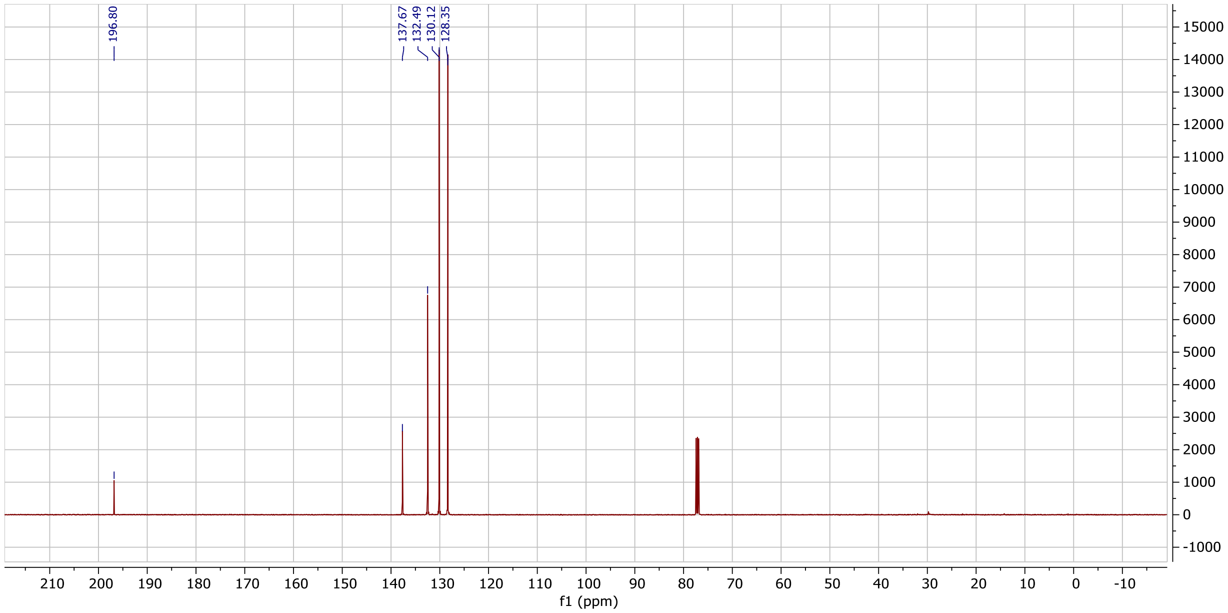

^{1}H NMR and ^{13}C NMR of 1b

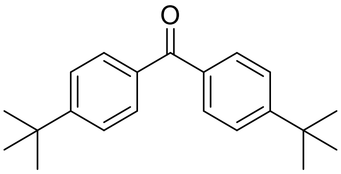

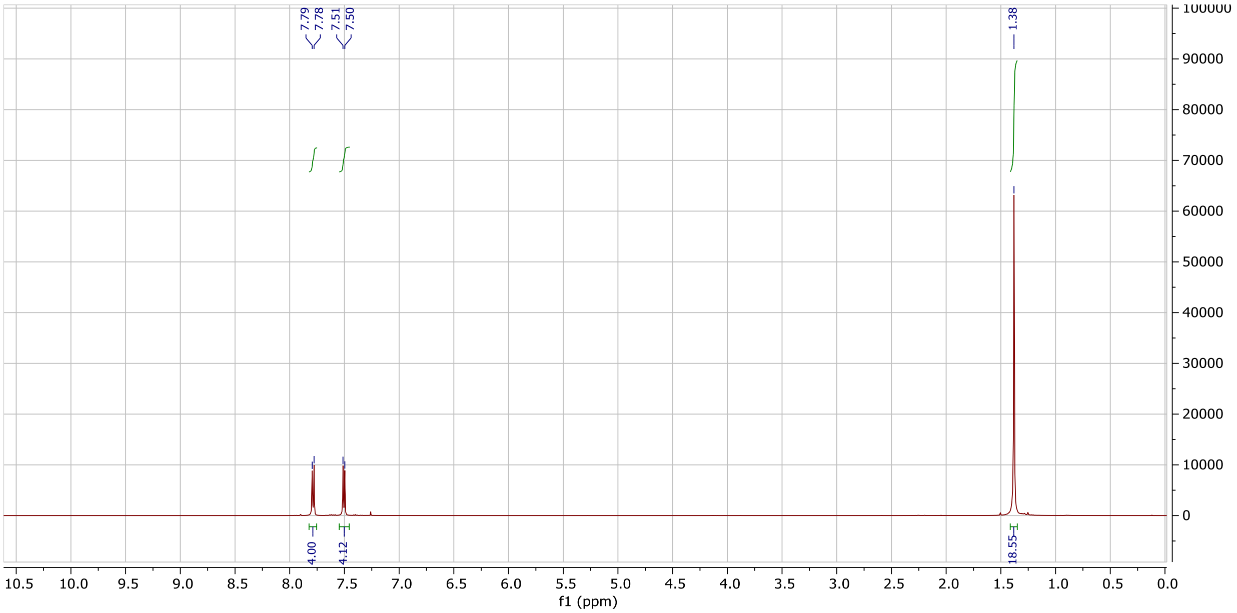

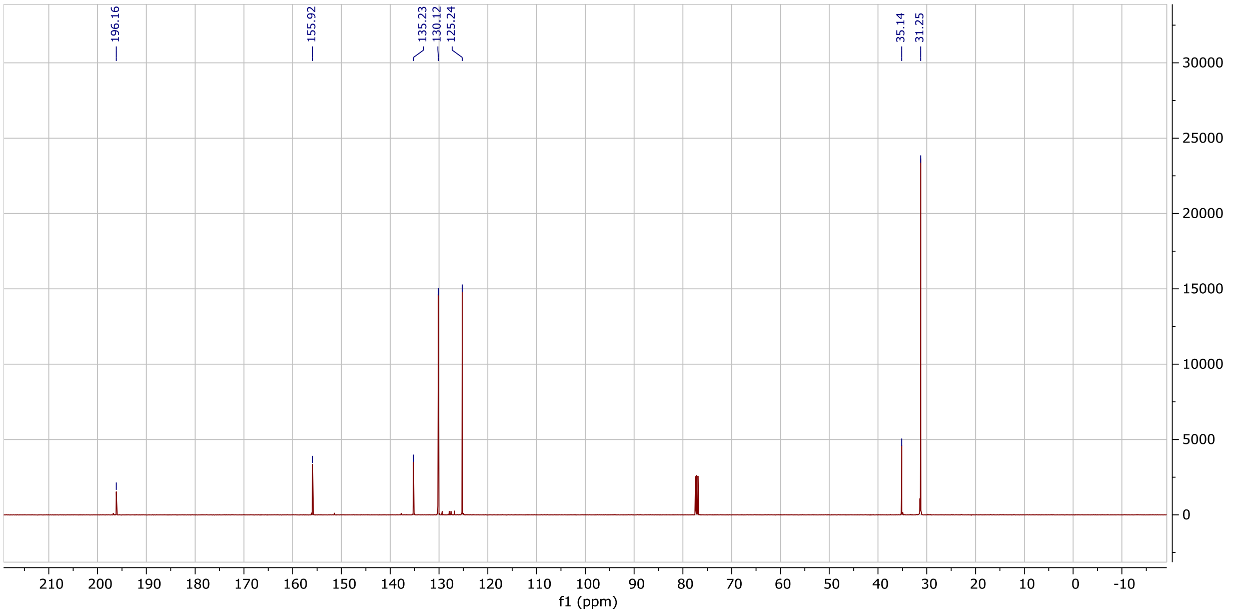

^{1}H NMR and ^{13}C NMR of 1c and 1c’

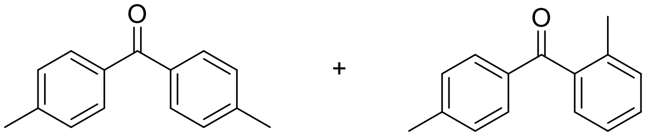

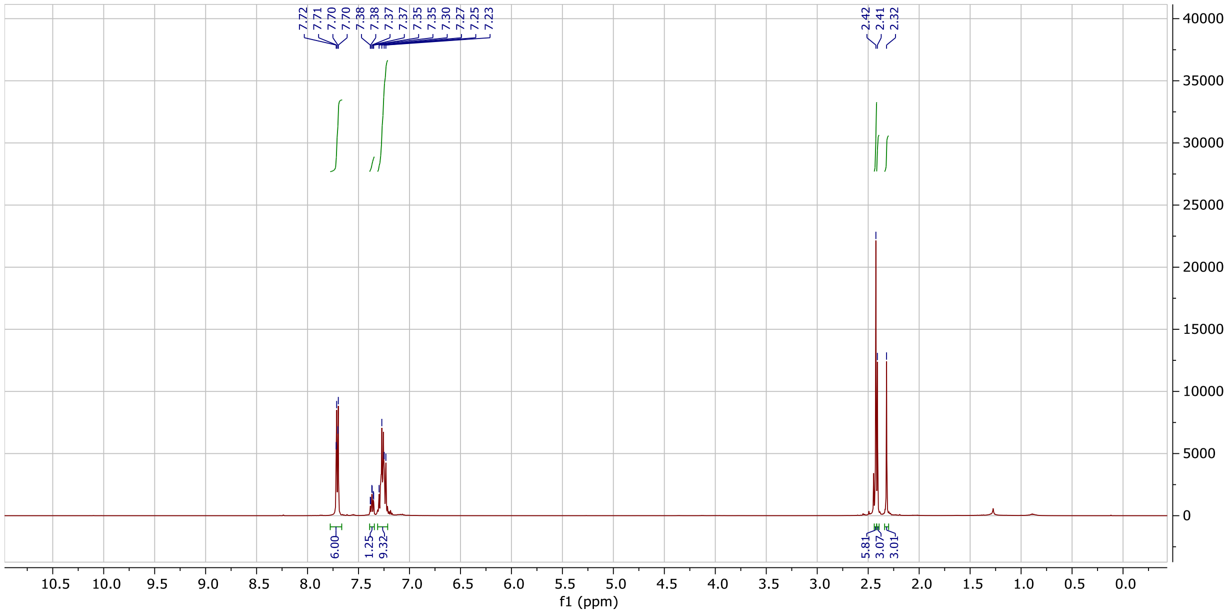

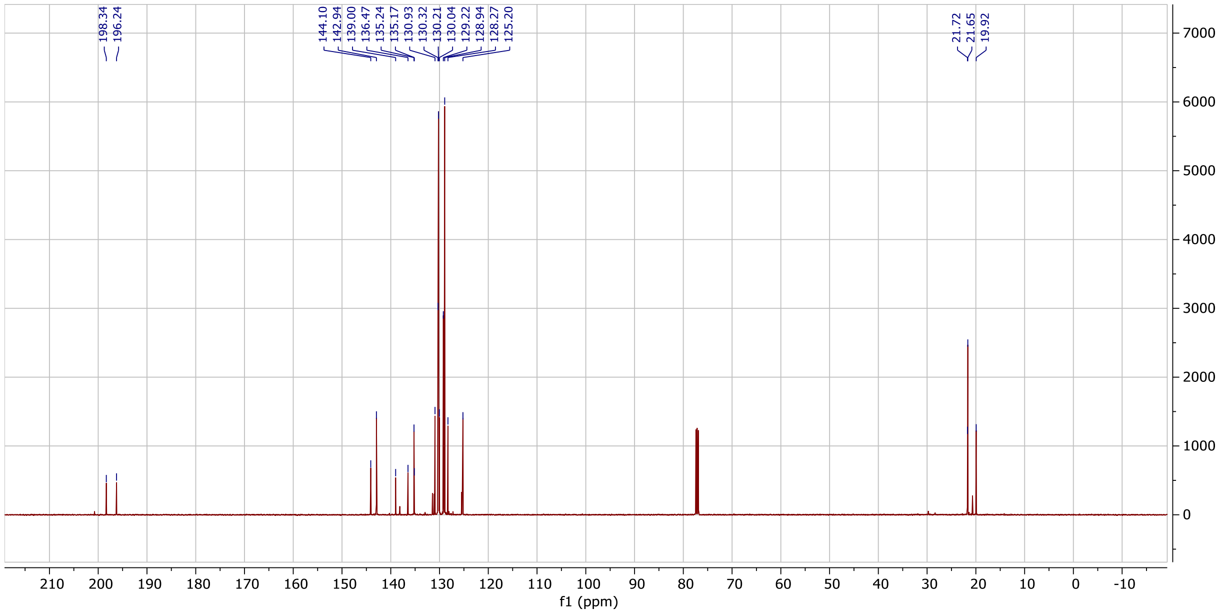

^{1}H NMR and ^{13}C NMR of 1d

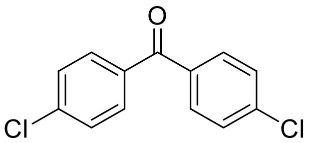

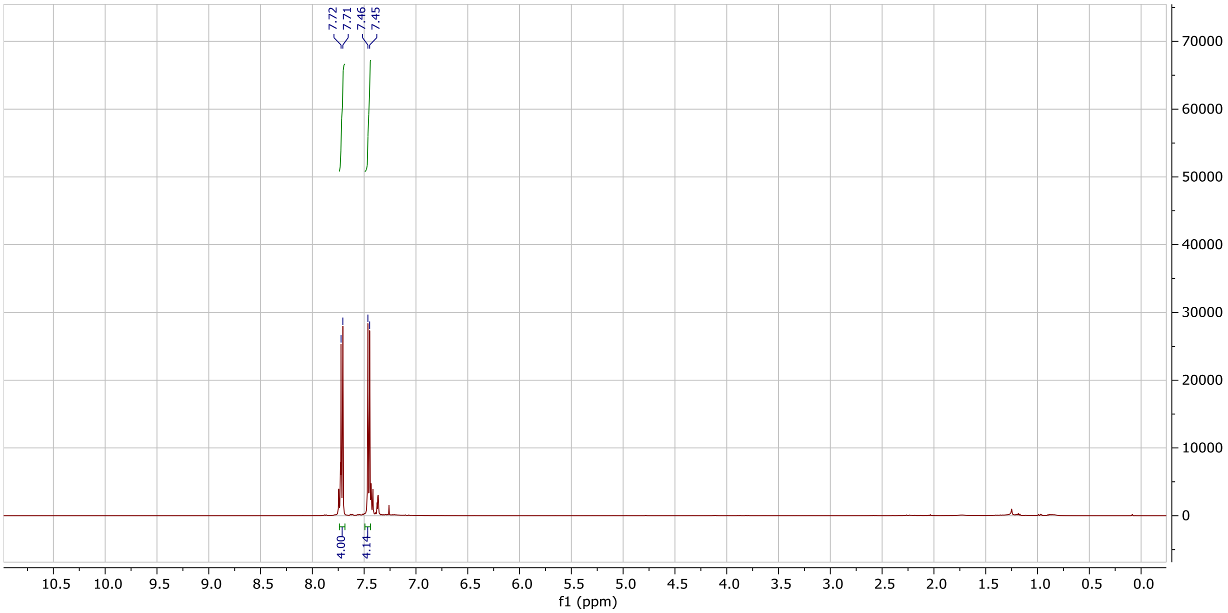

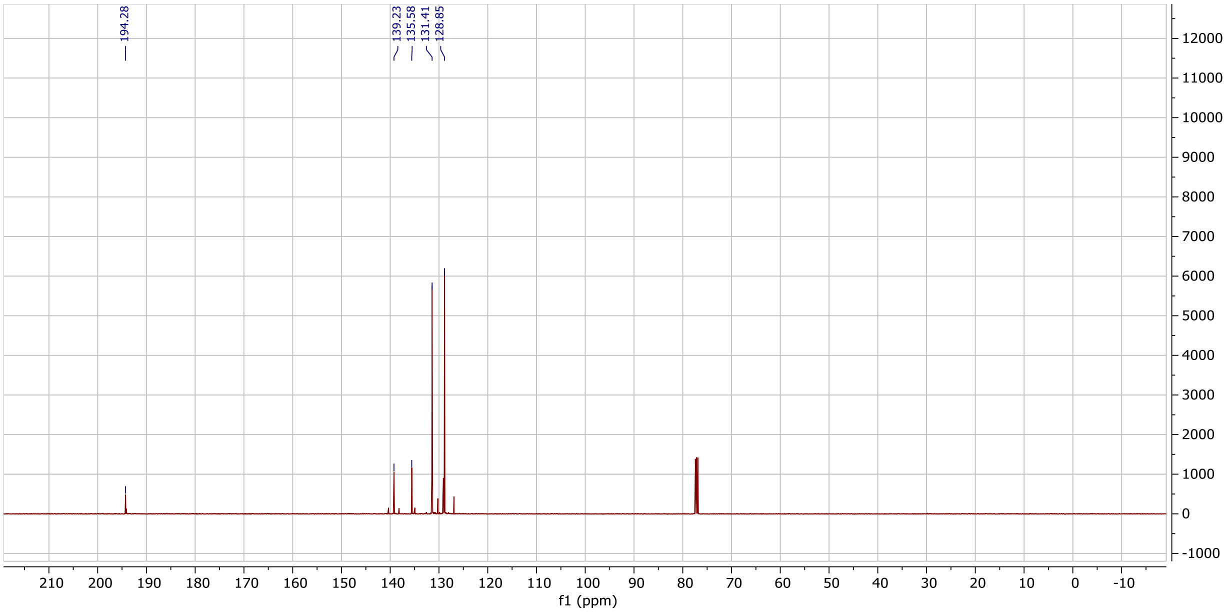

^{1}H NMR and ^{13}C NMR of 1e

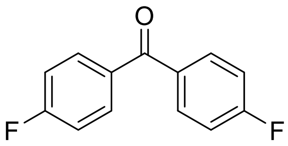

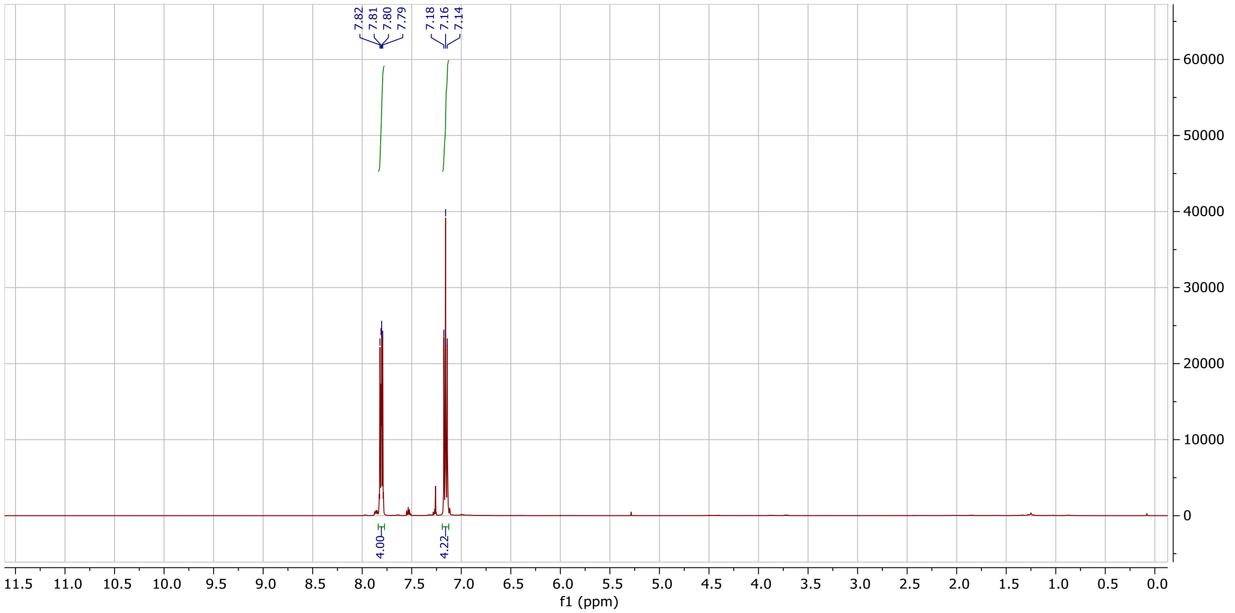

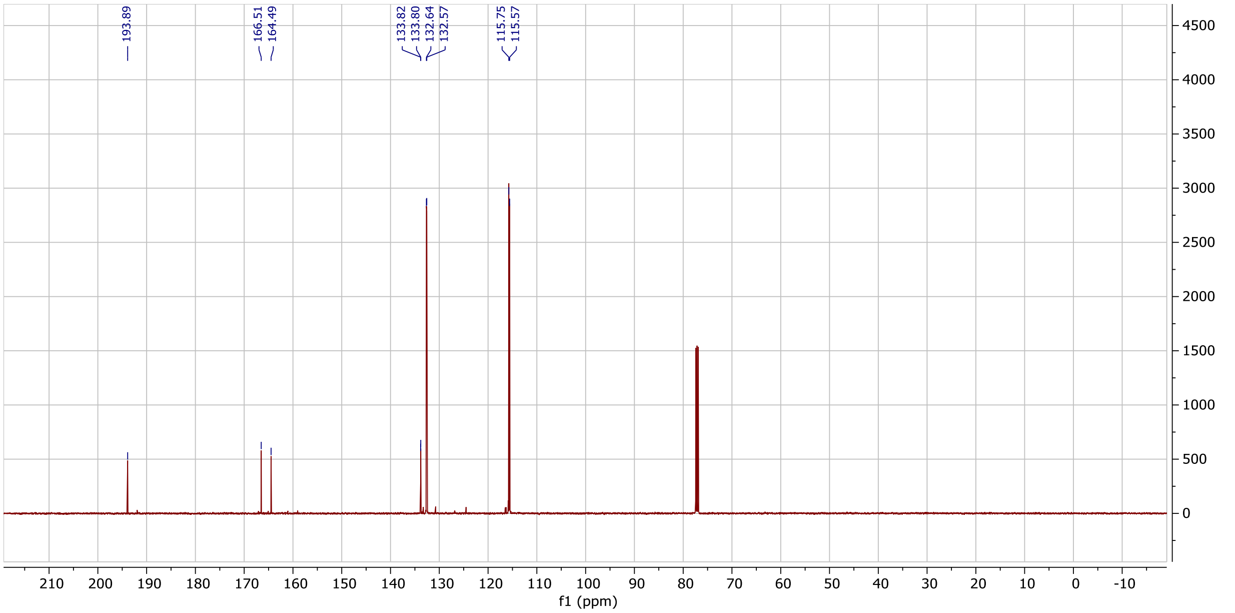

^{1}H NMR and ^{13}C NMR of 1f

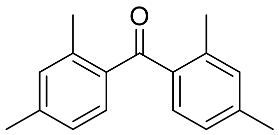

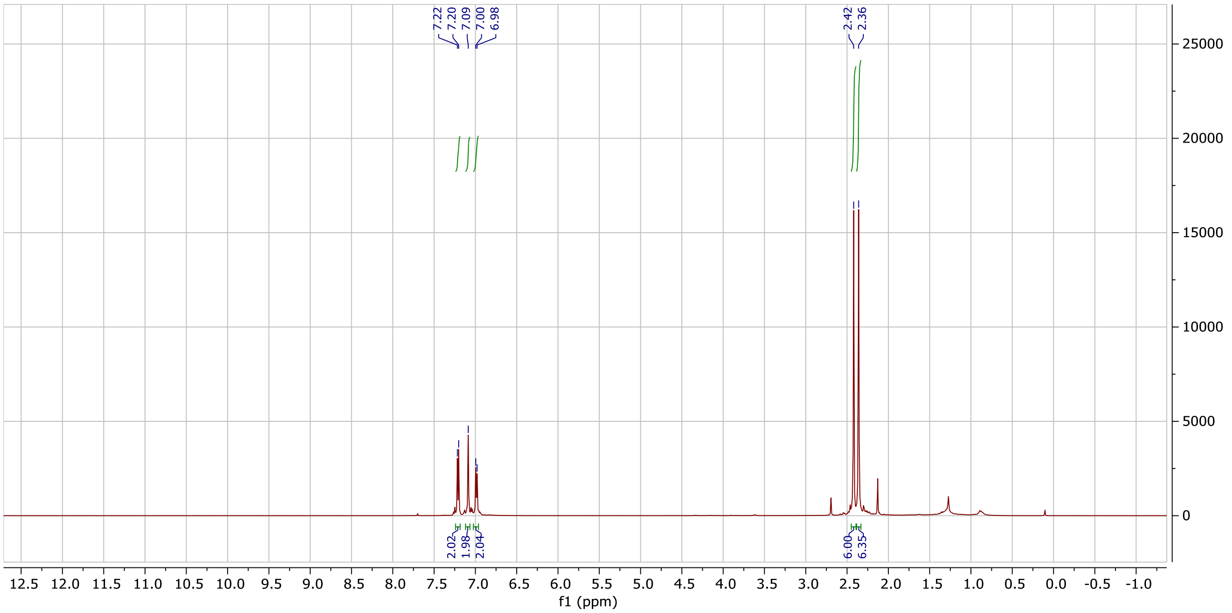

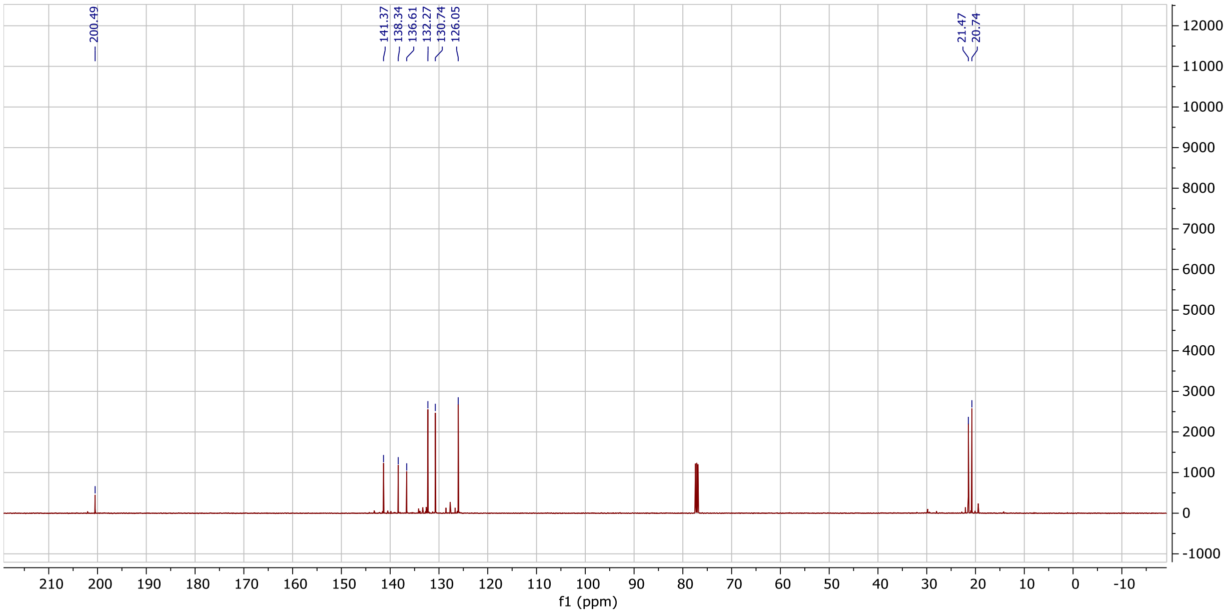

^{1}H NMR and ^{13}C NMR of 1g

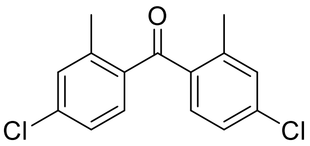

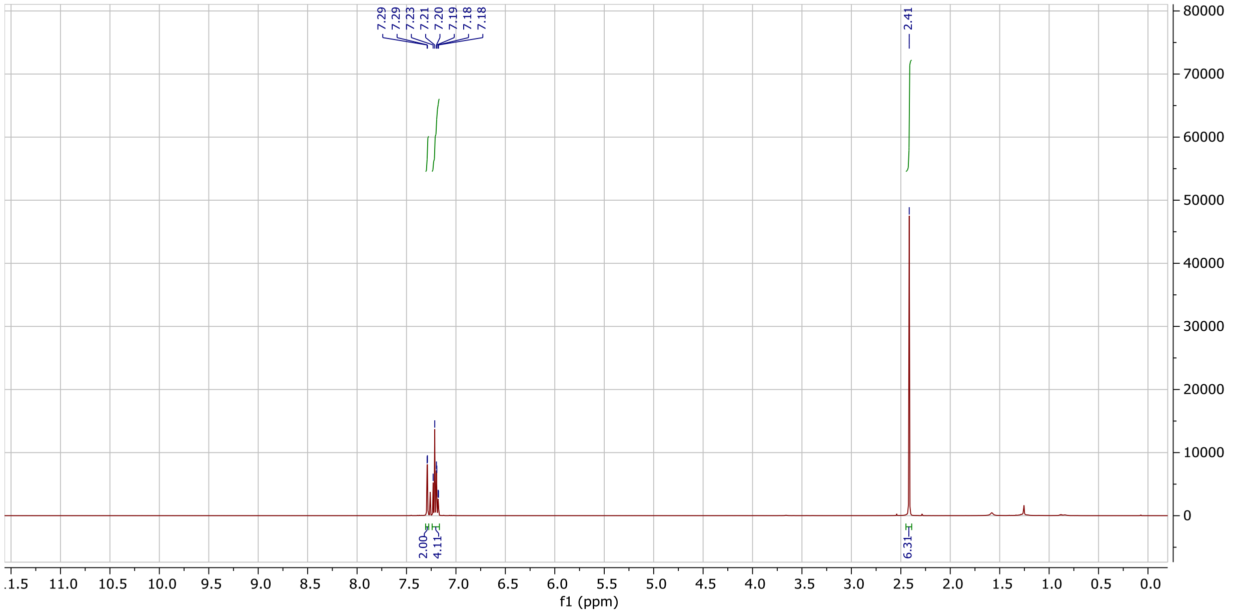

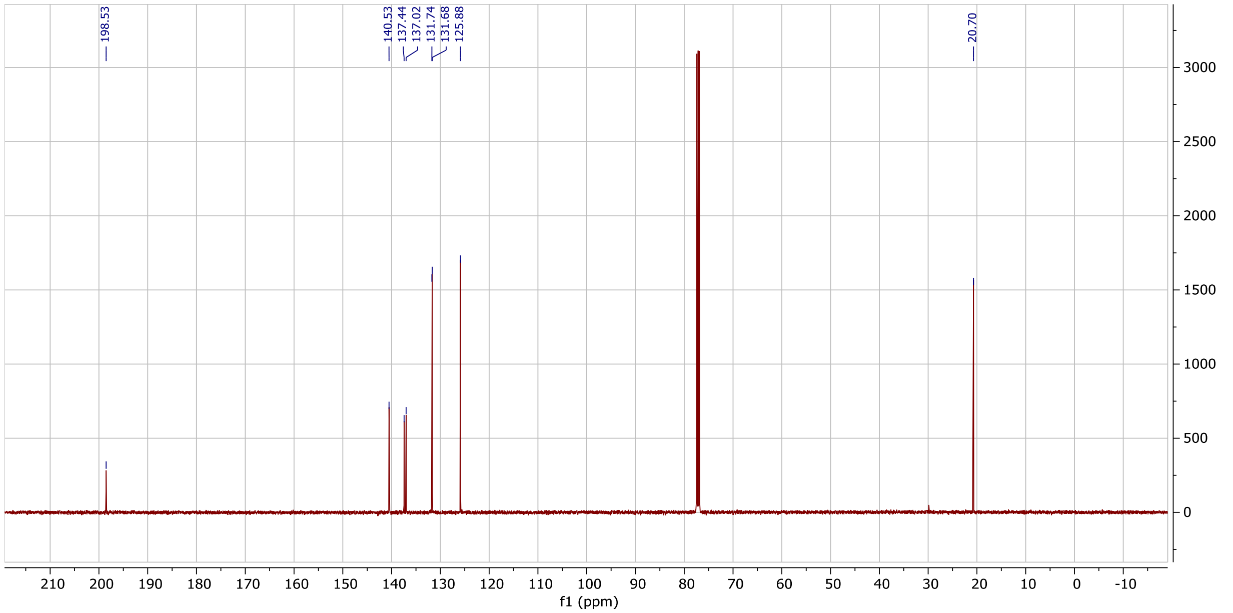

SelNOE NMR spectrum for 1g

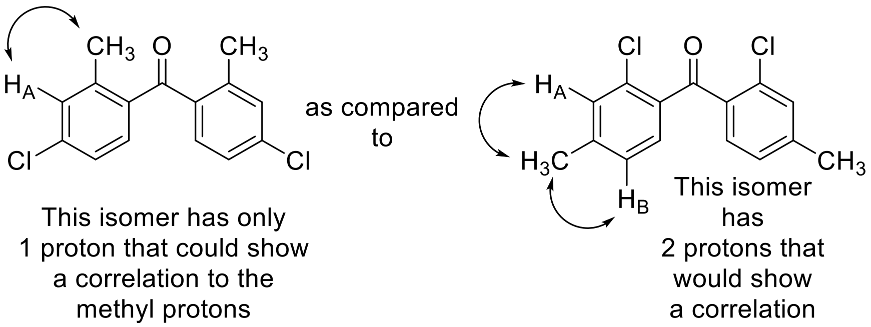

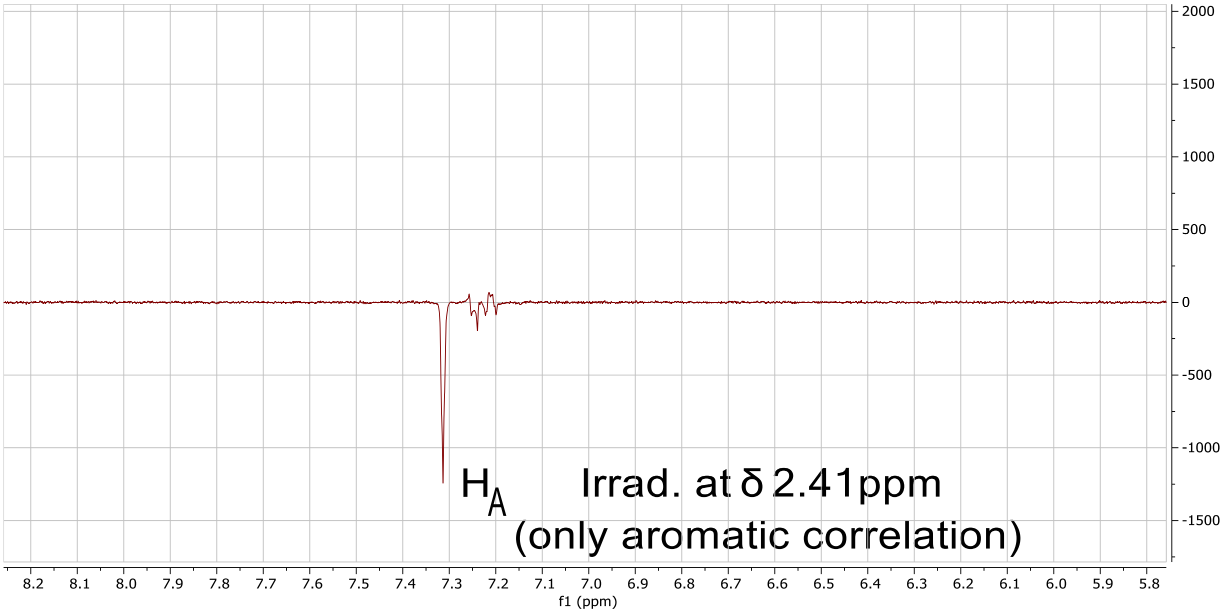

^{1}H NMR and ^{13}C NMR of 1g'

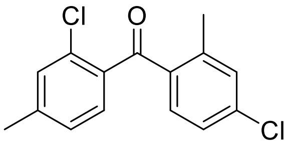

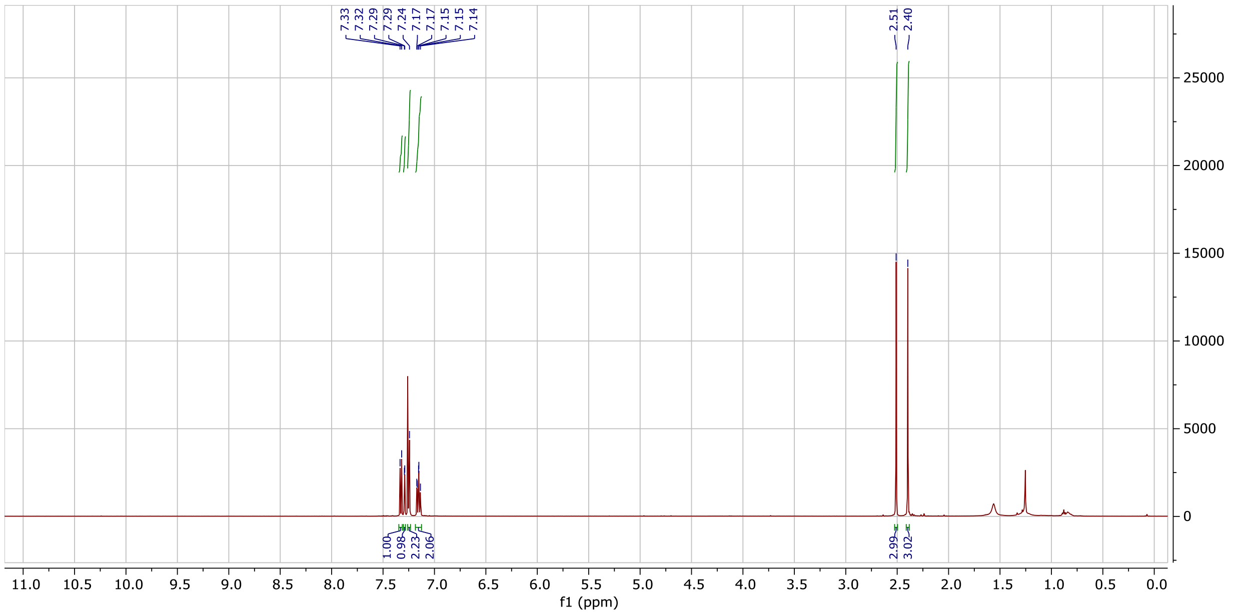

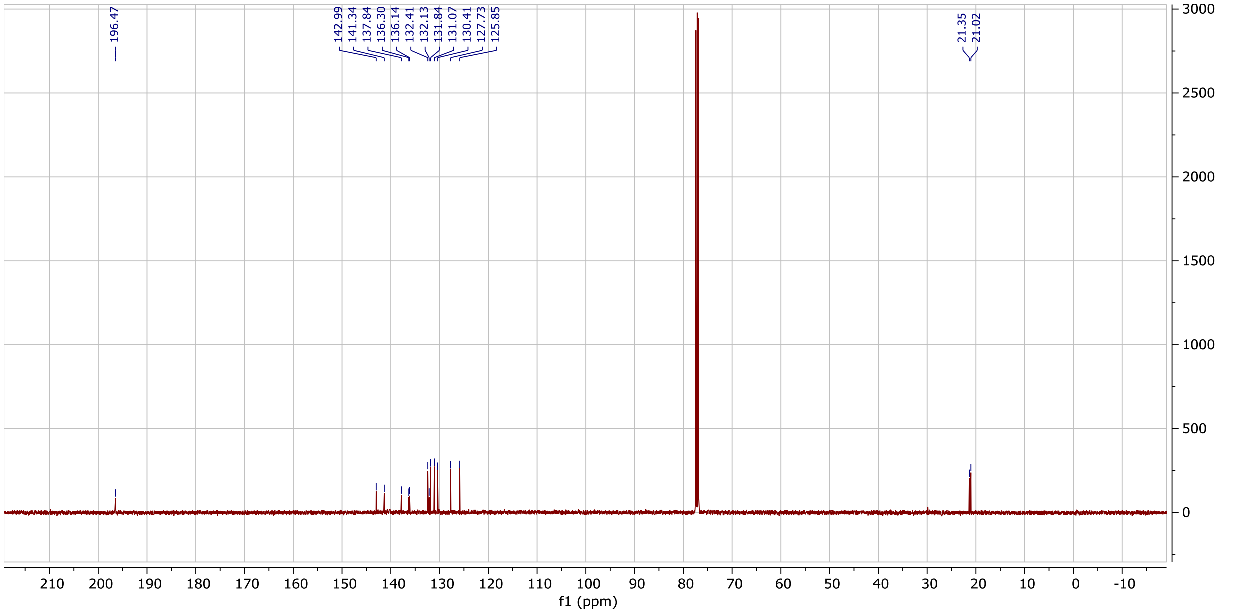

^{1}H NMR and ^{13}C NMR of 1h

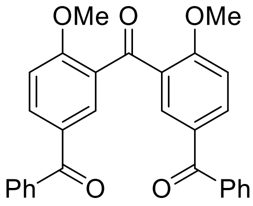

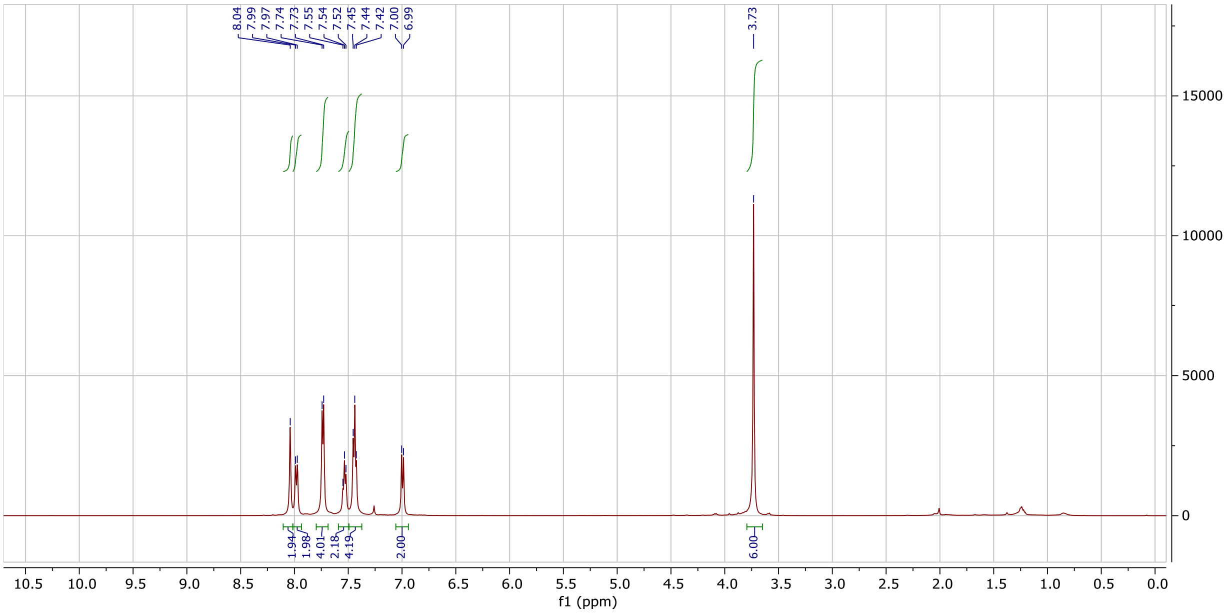

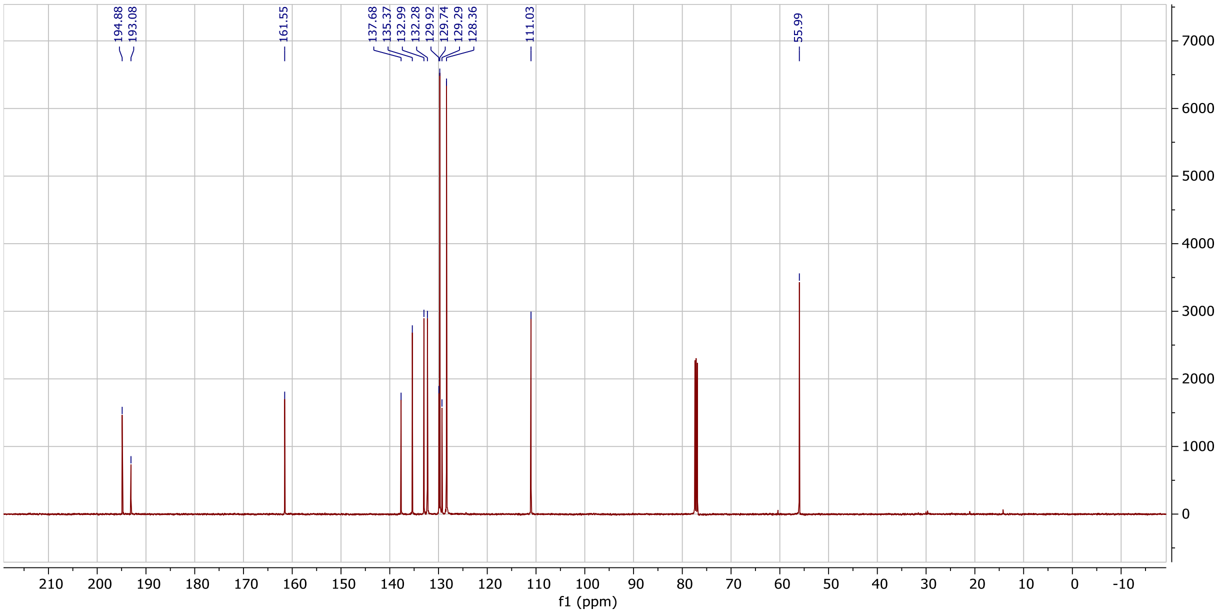

^1H NMR and ^{13}C NMR of 1i and 1i’

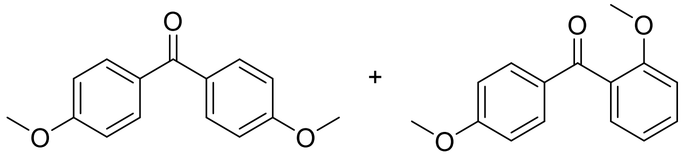

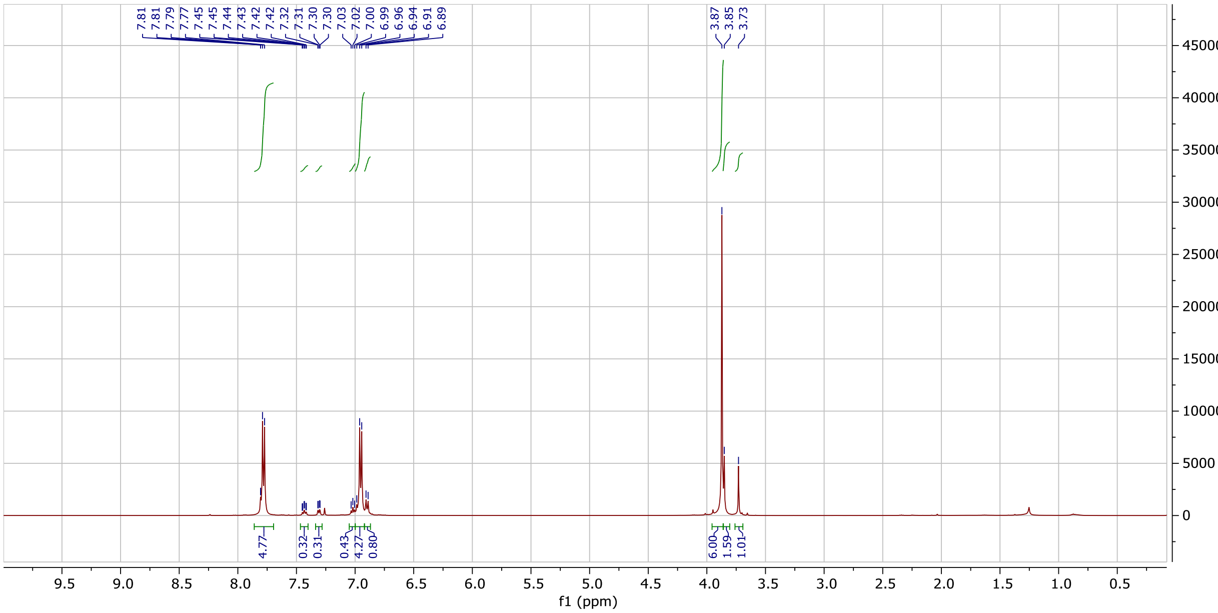

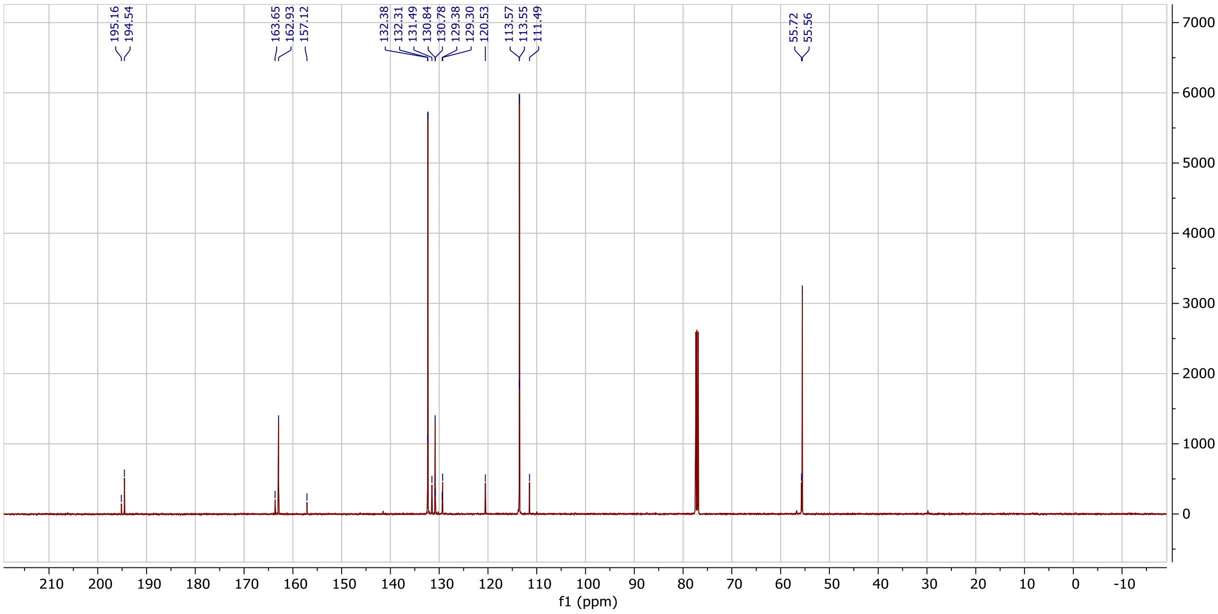

^{1}H NMR and ^{13}C NMR of 1j

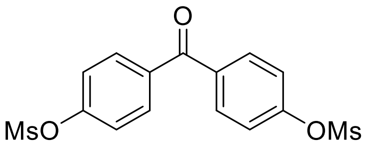

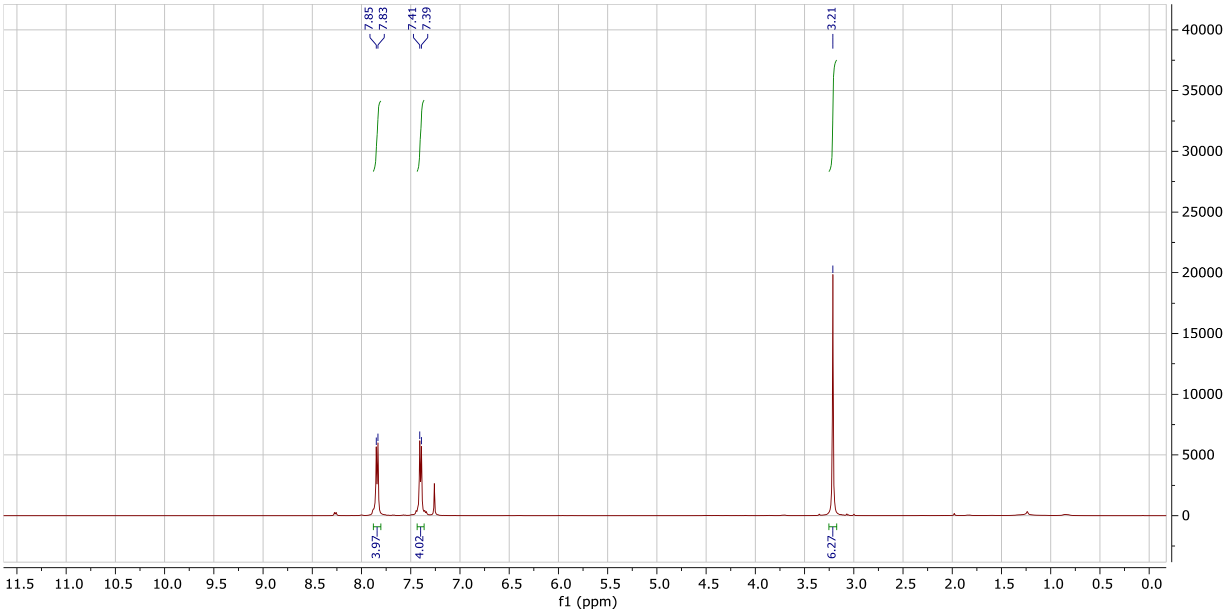

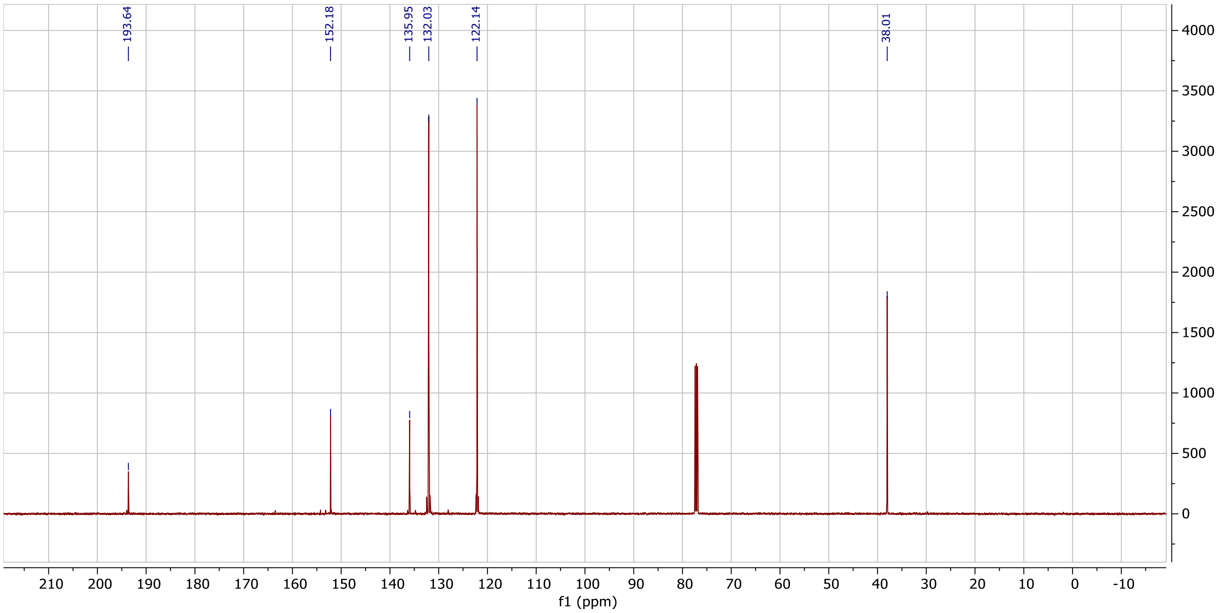

^{1}H NMR and ^{13}C NMR of 1k

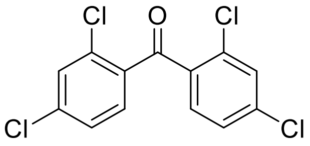

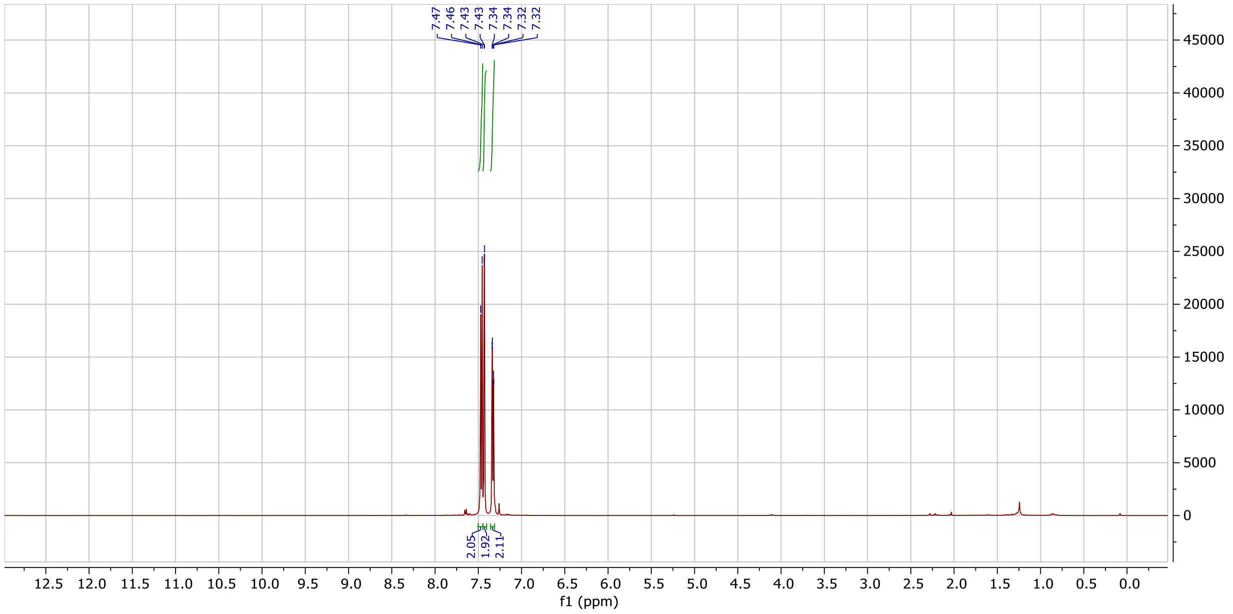

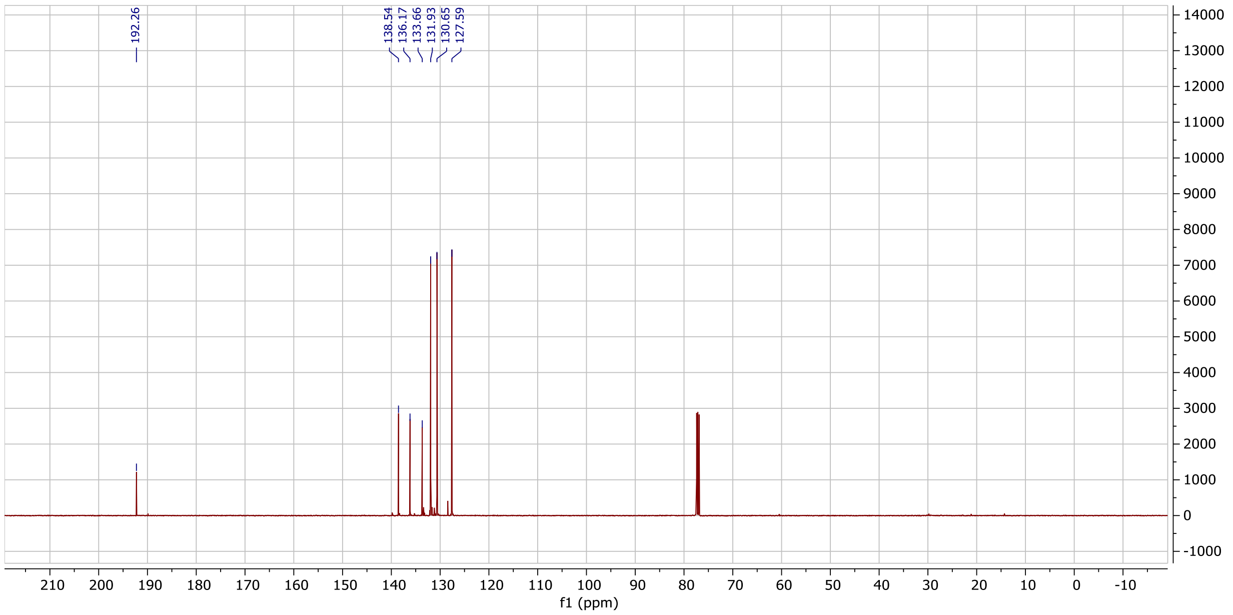

^{1}H NMR and ^{13}C NMR of 1l

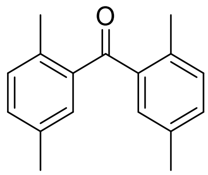

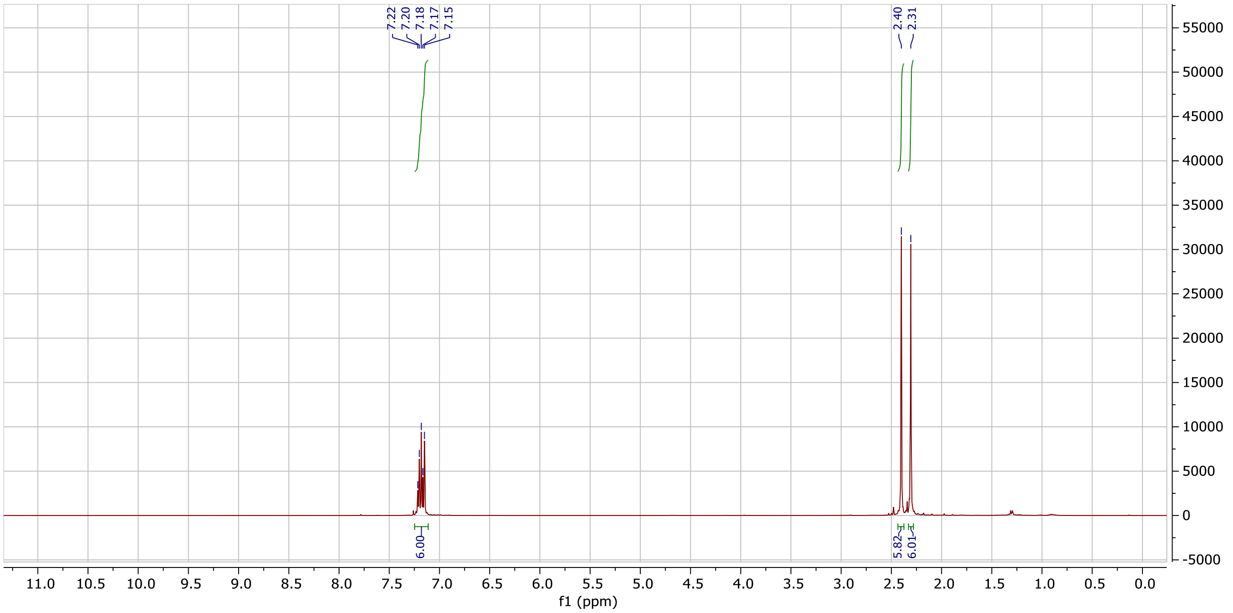

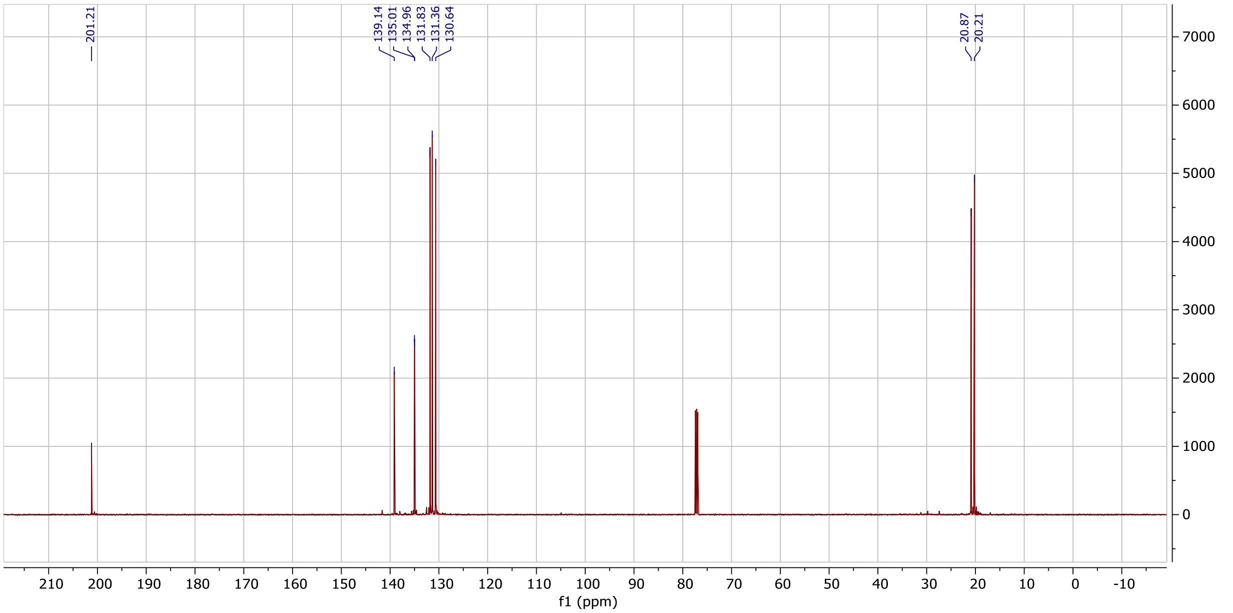

^{1}H NMR and ^{13}C NMR of 1m and 1m’

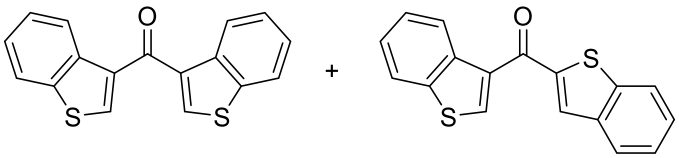

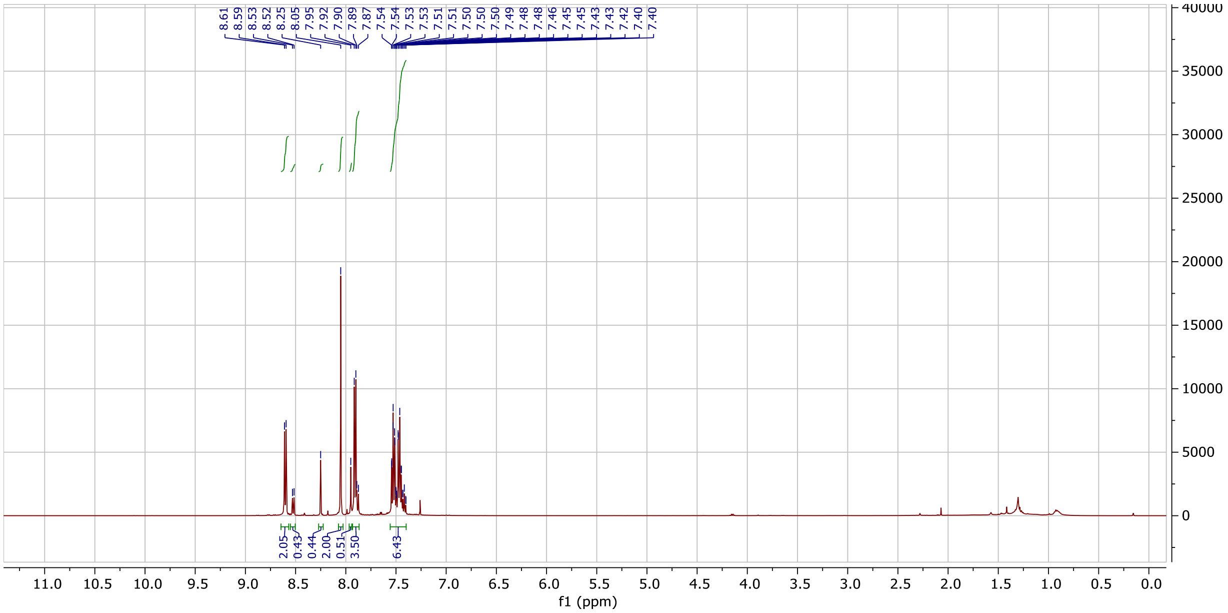

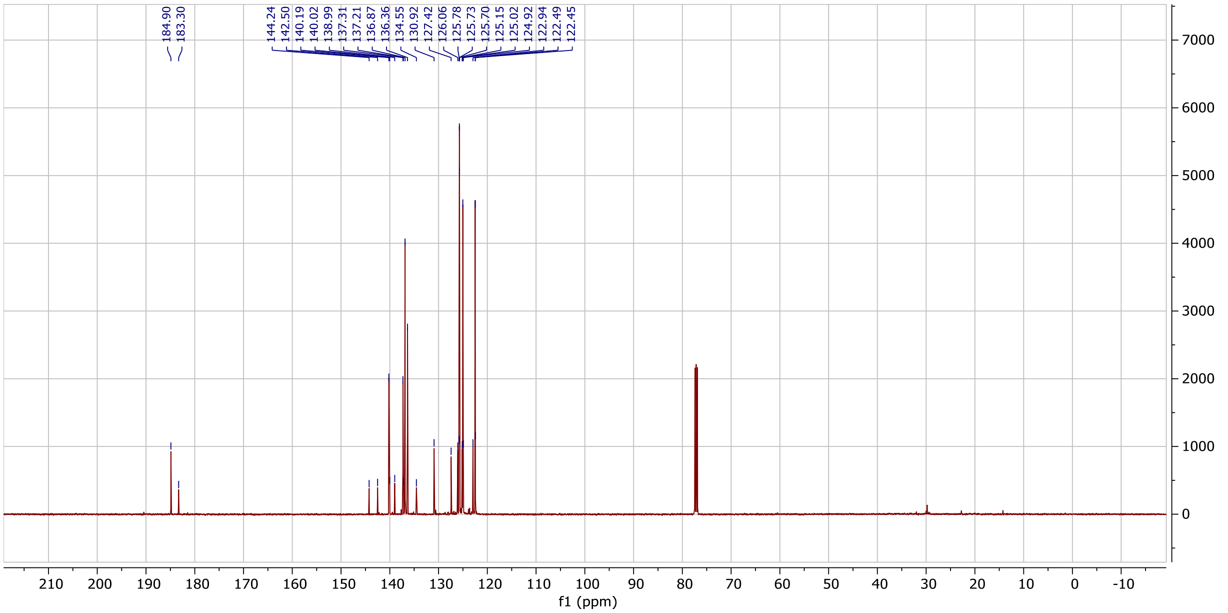

^{1}H NMR and ^{13}C NMR of 1n

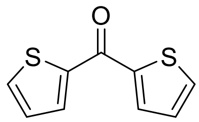

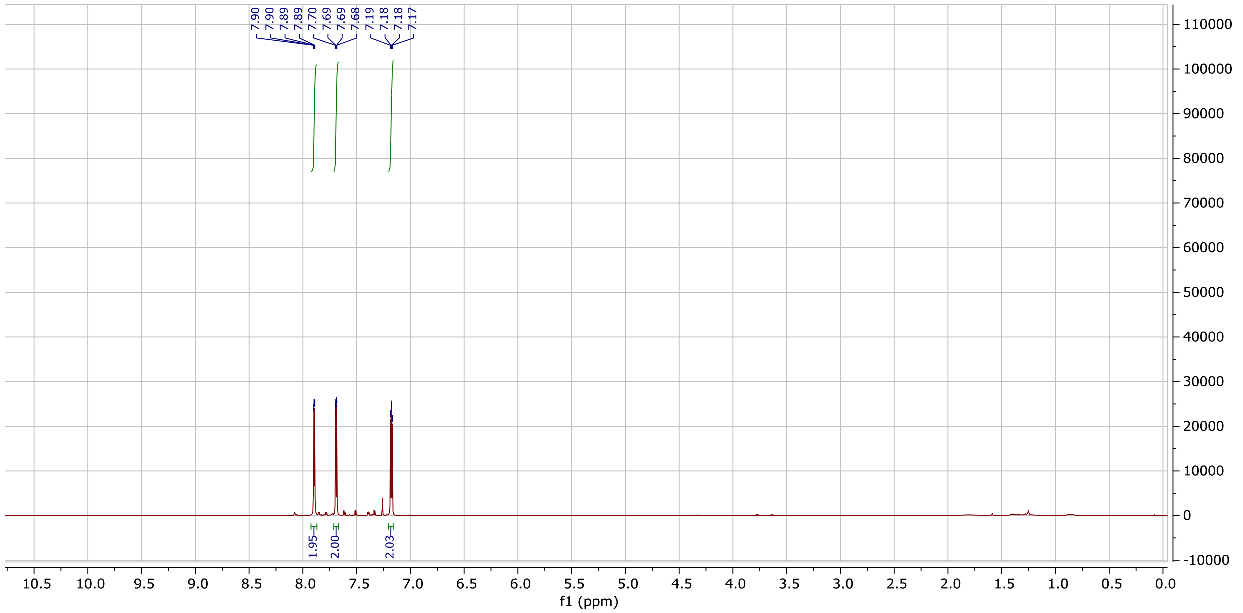

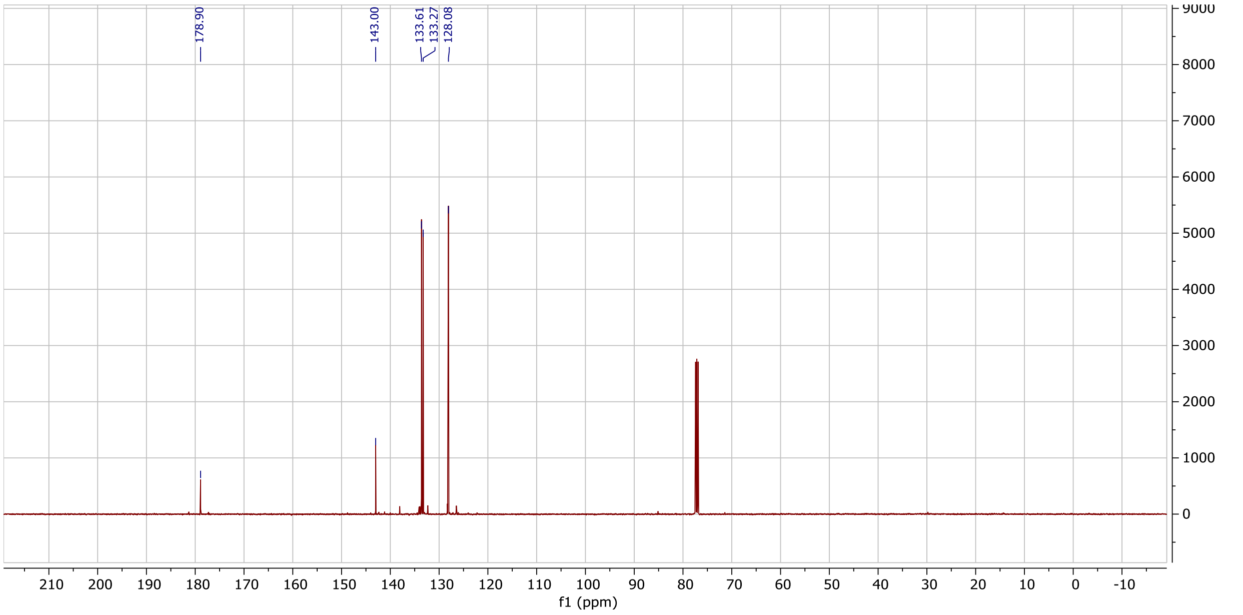

^{1}H NMR and ^{13}C NMR of 1o

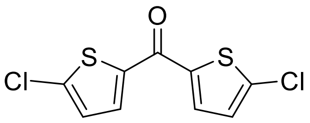

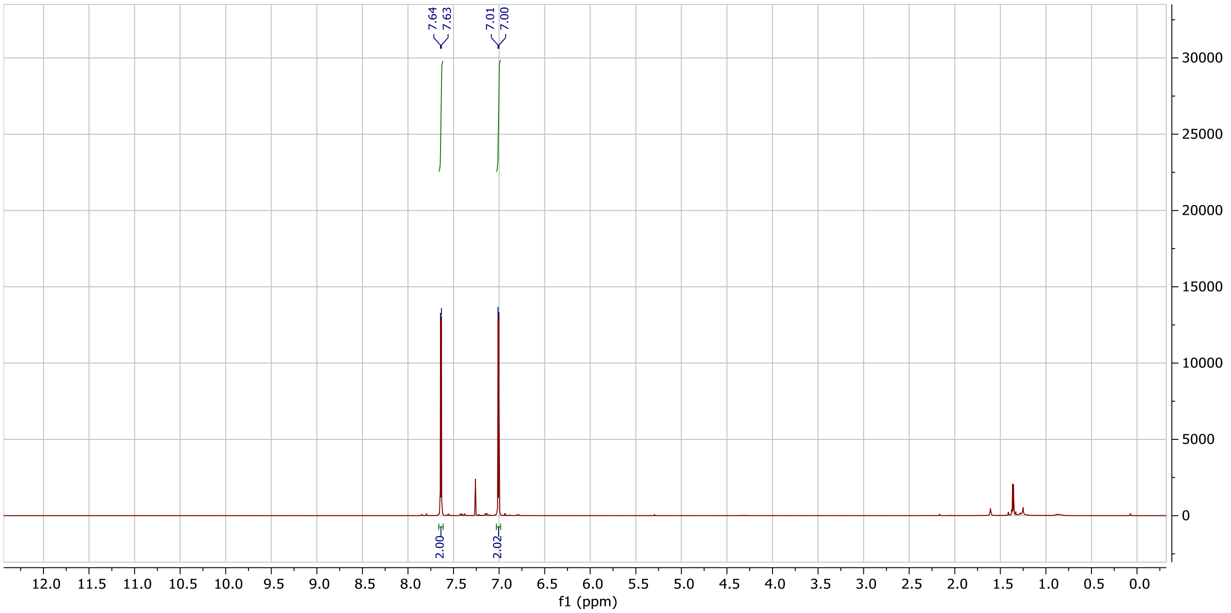

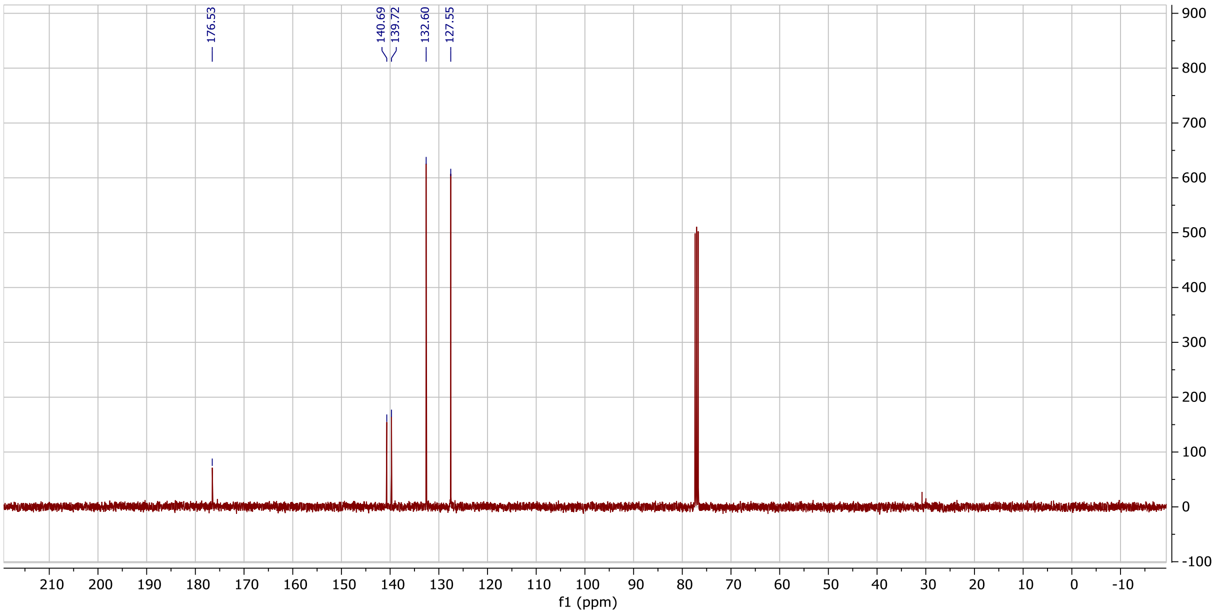

^{1}H NMR and ^{13}C NMR of 1p

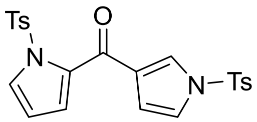

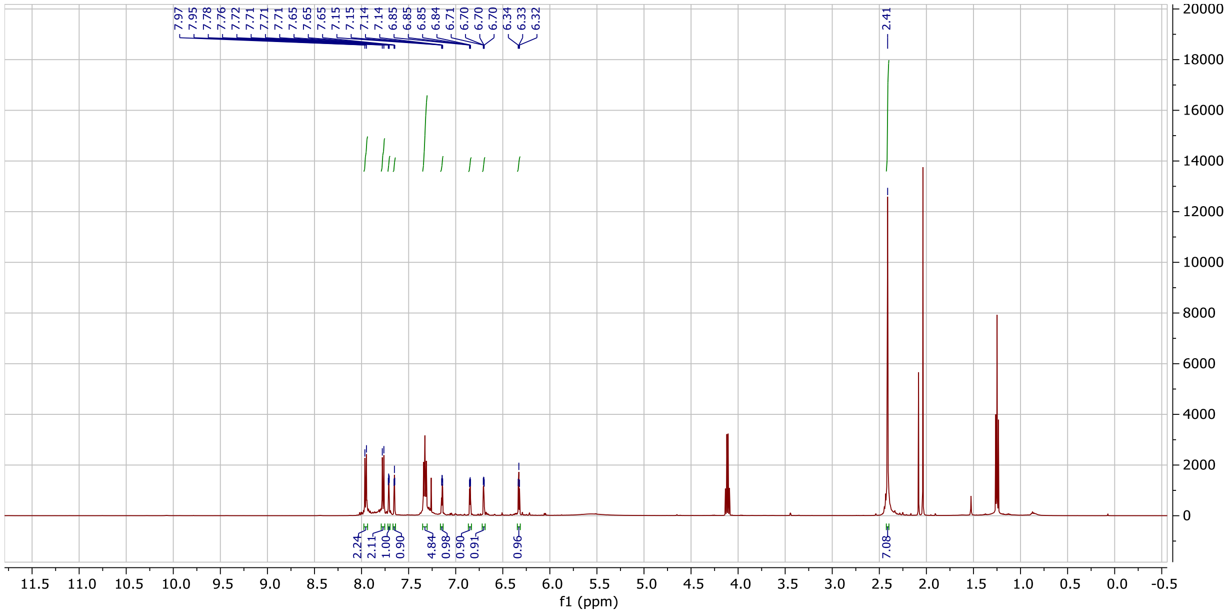

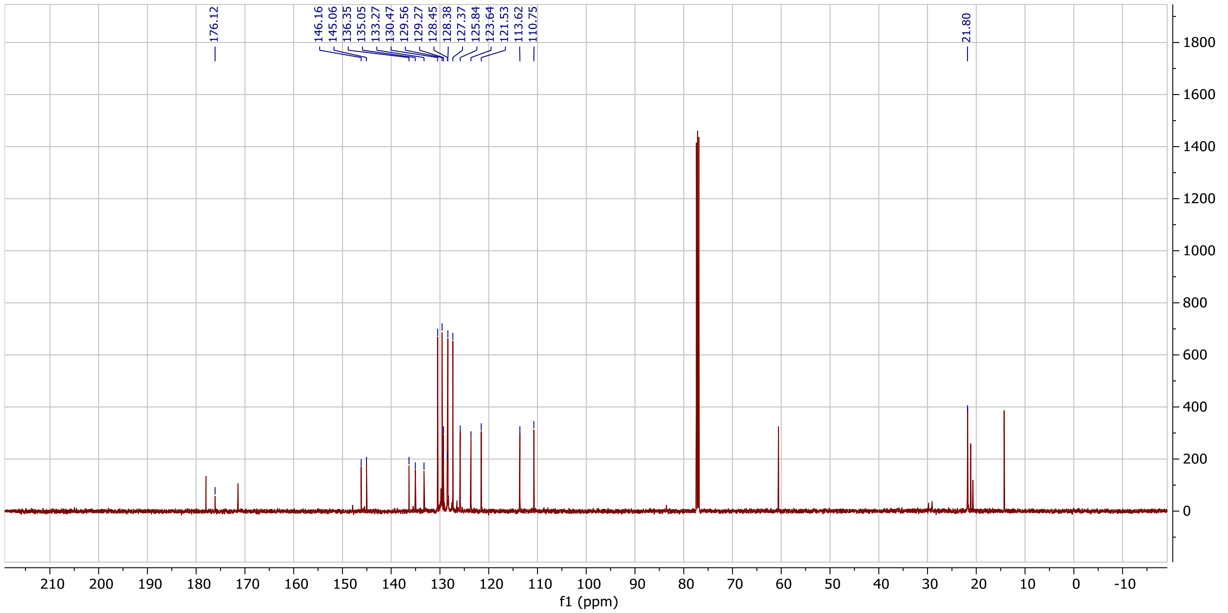

^{1}H NMR and ^{13}C NMR of 1p’

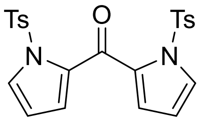

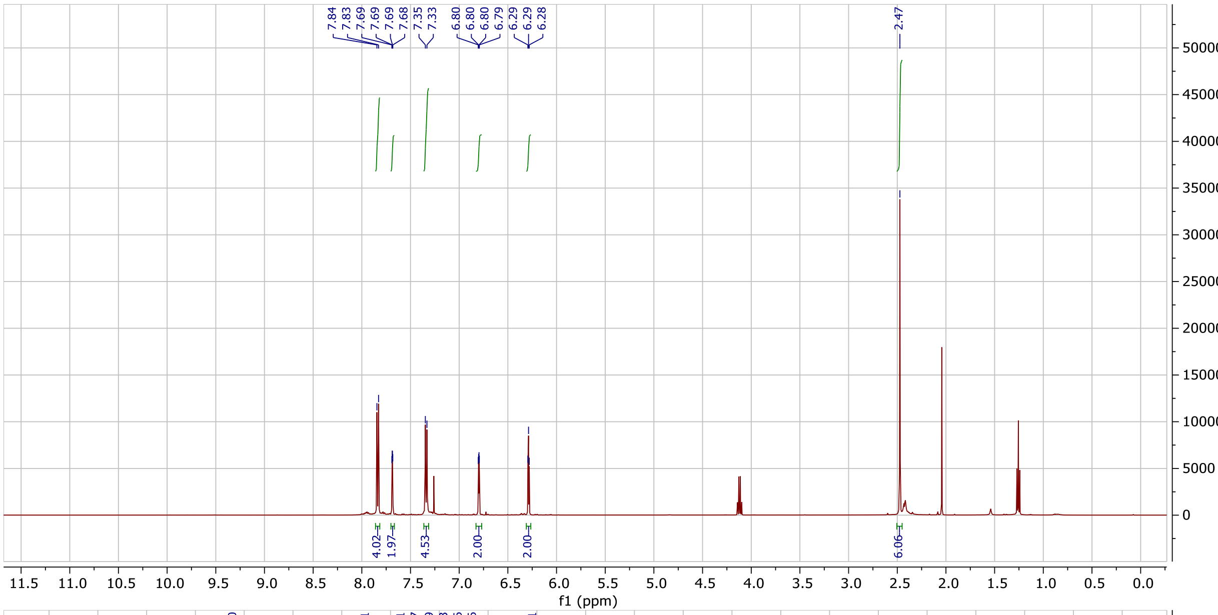

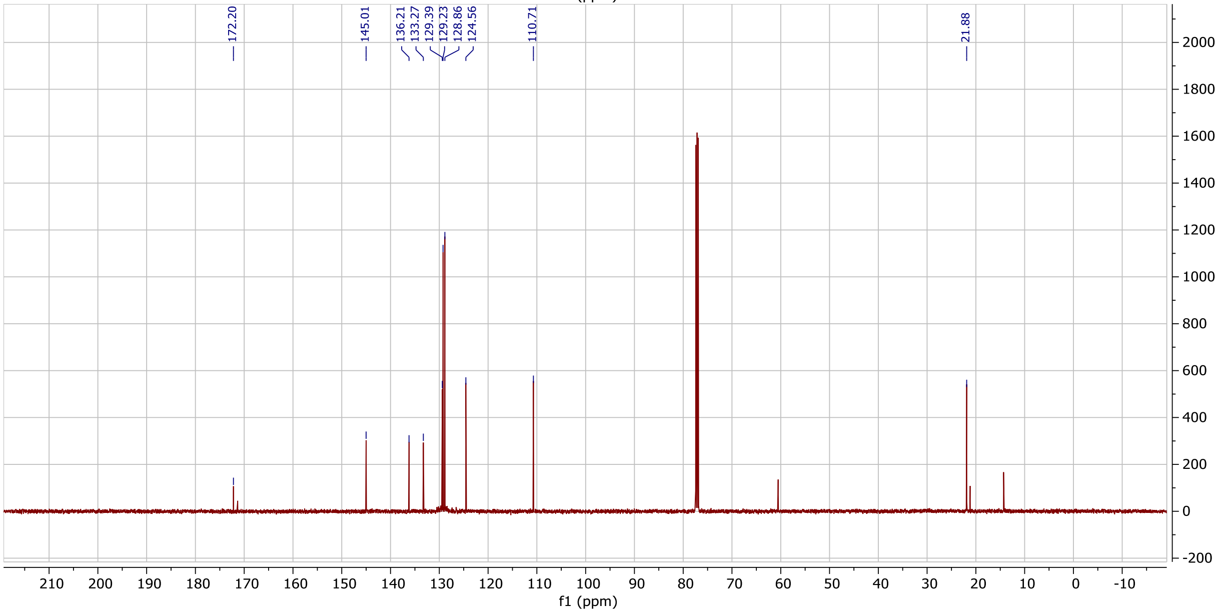

^{1}H NMR and ^{13}C NMR of 2a

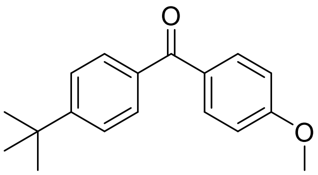

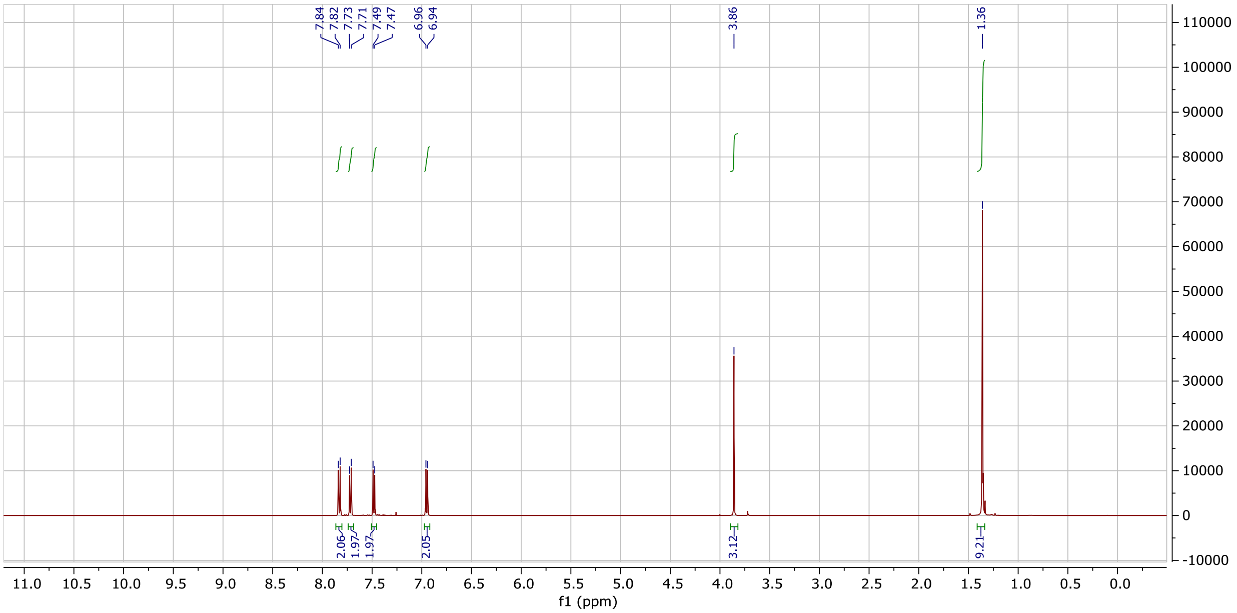

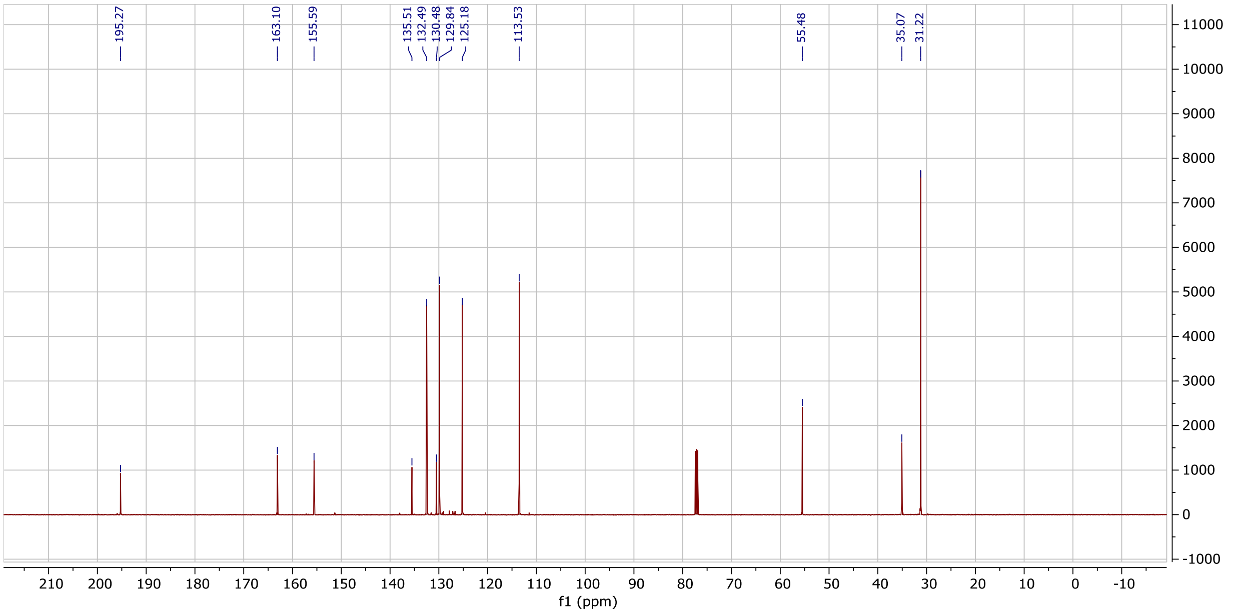

^{1}H NMR and ^{13}C NMR of 2b

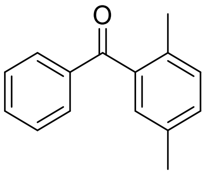

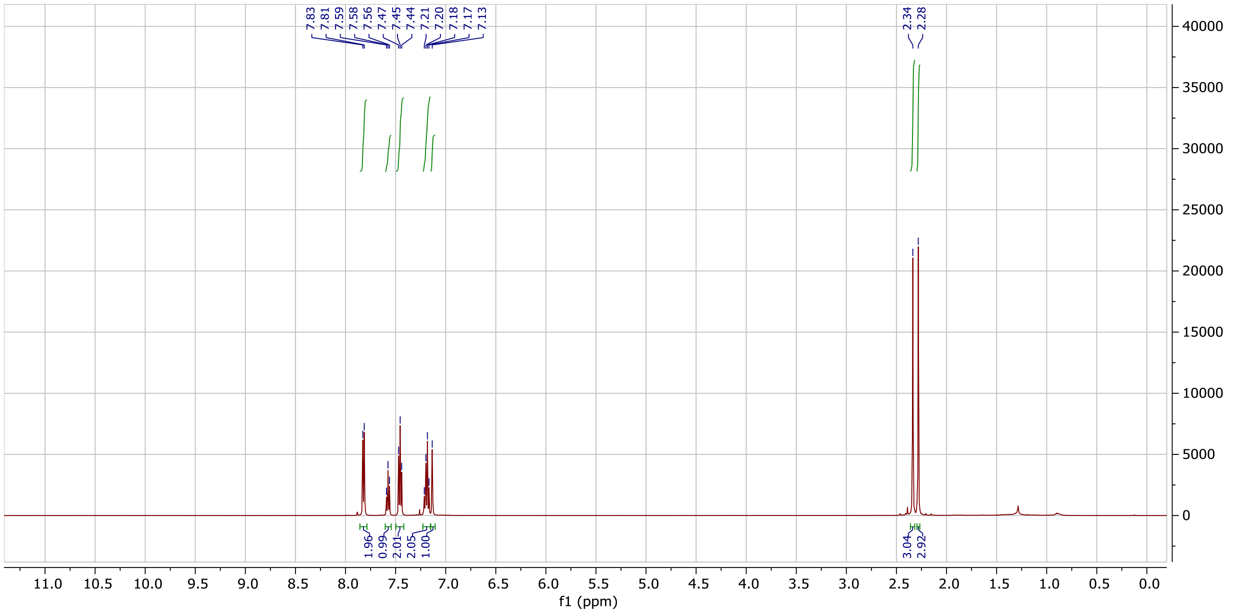

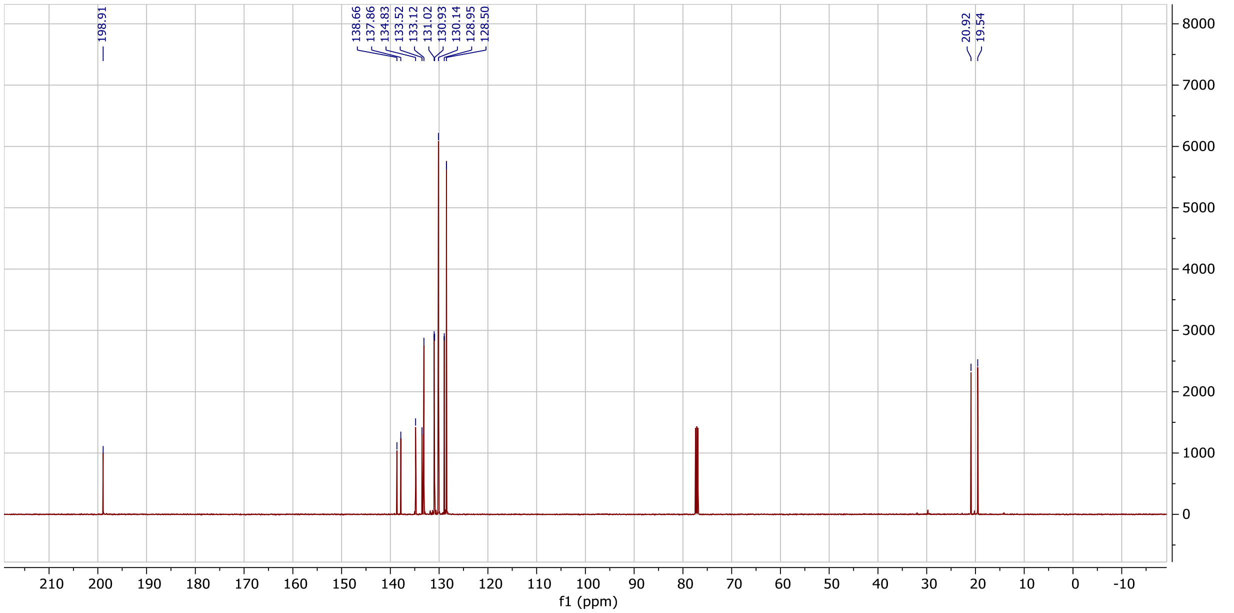

^{1}H NMR and ^{13}C NMR of 2c

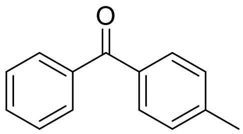

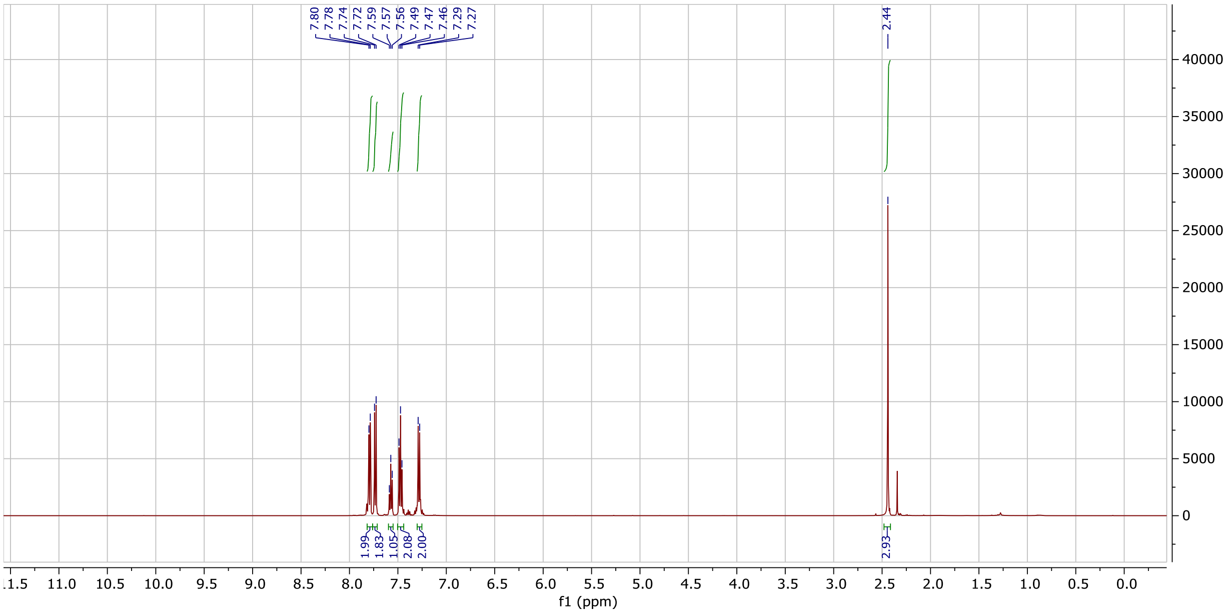

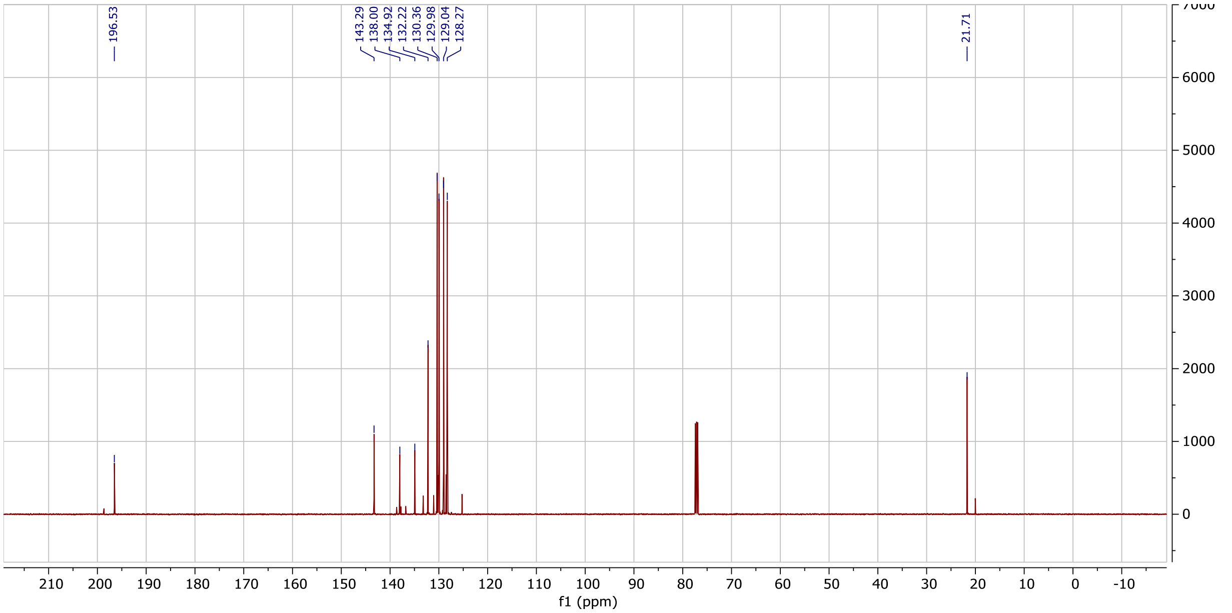

^{1}H NMR and ^{13}C NMR of 2d

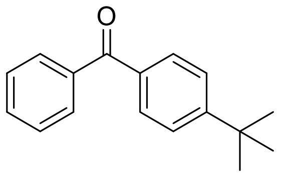

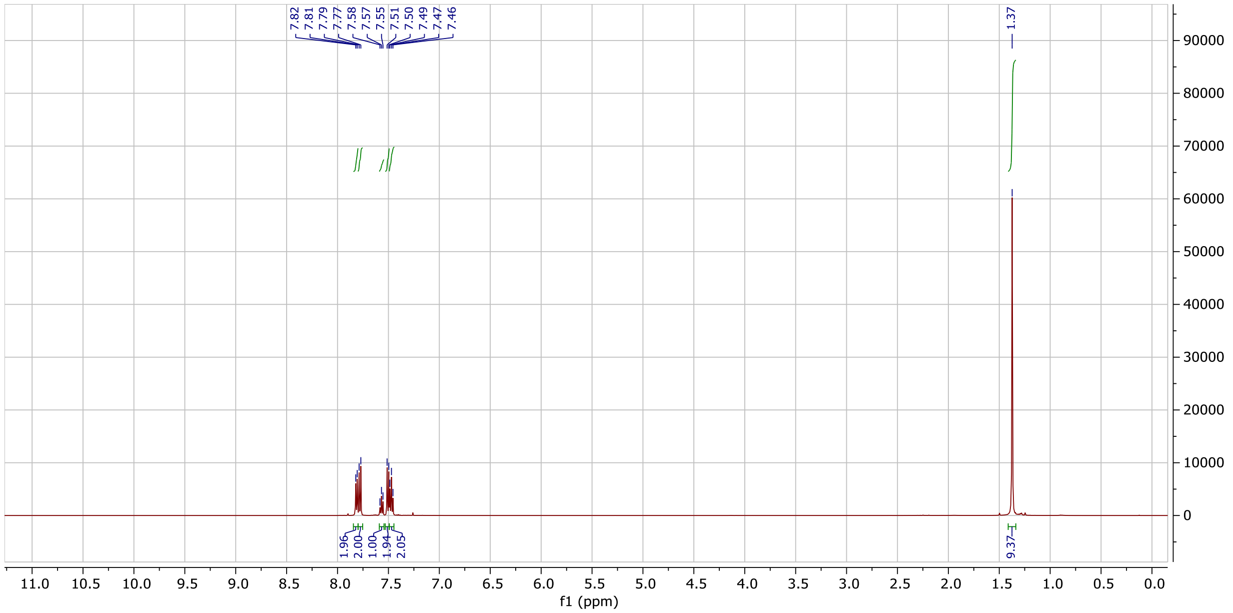

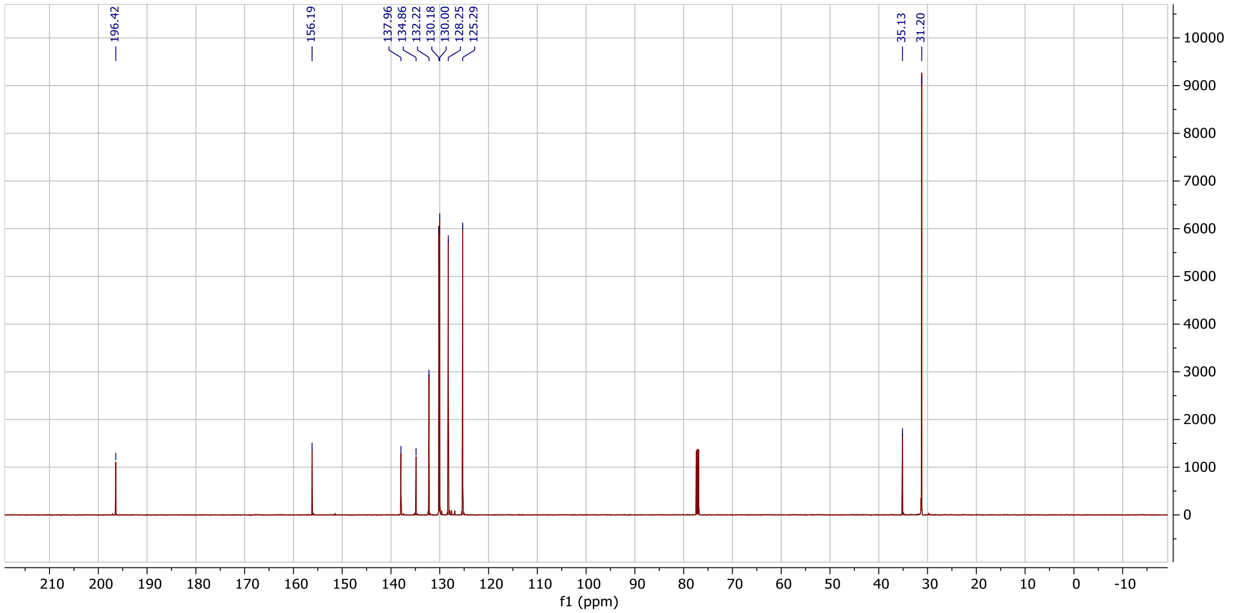

^{1}H NMR and ^{13}C NMR of 2e

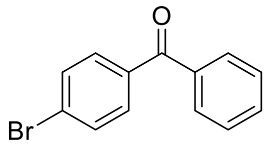

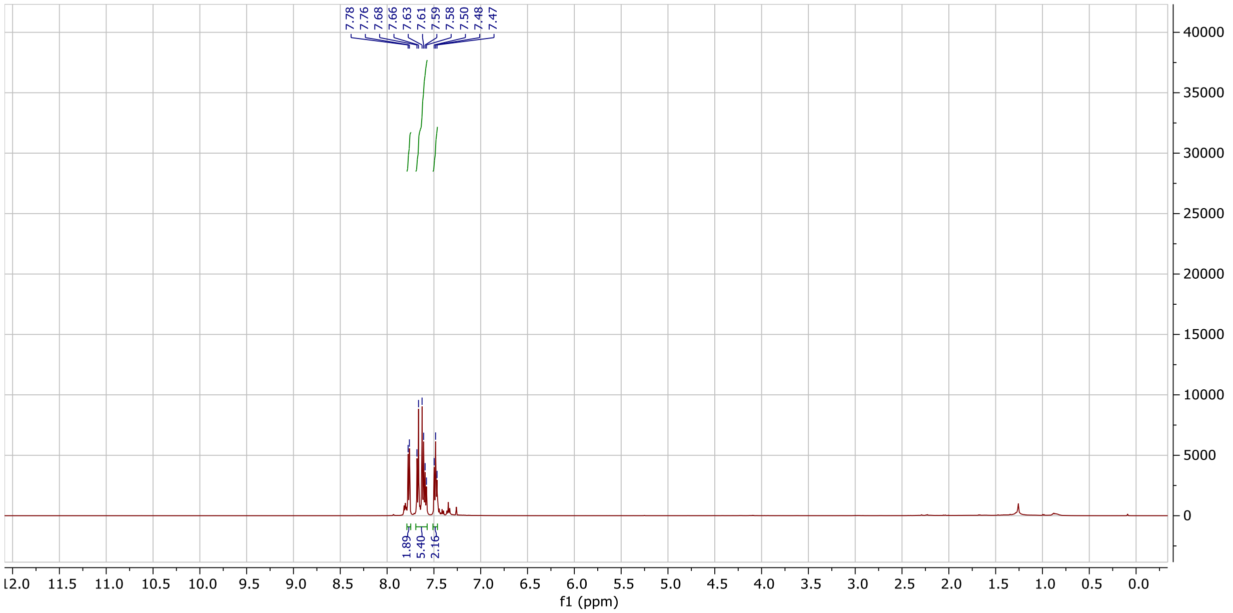

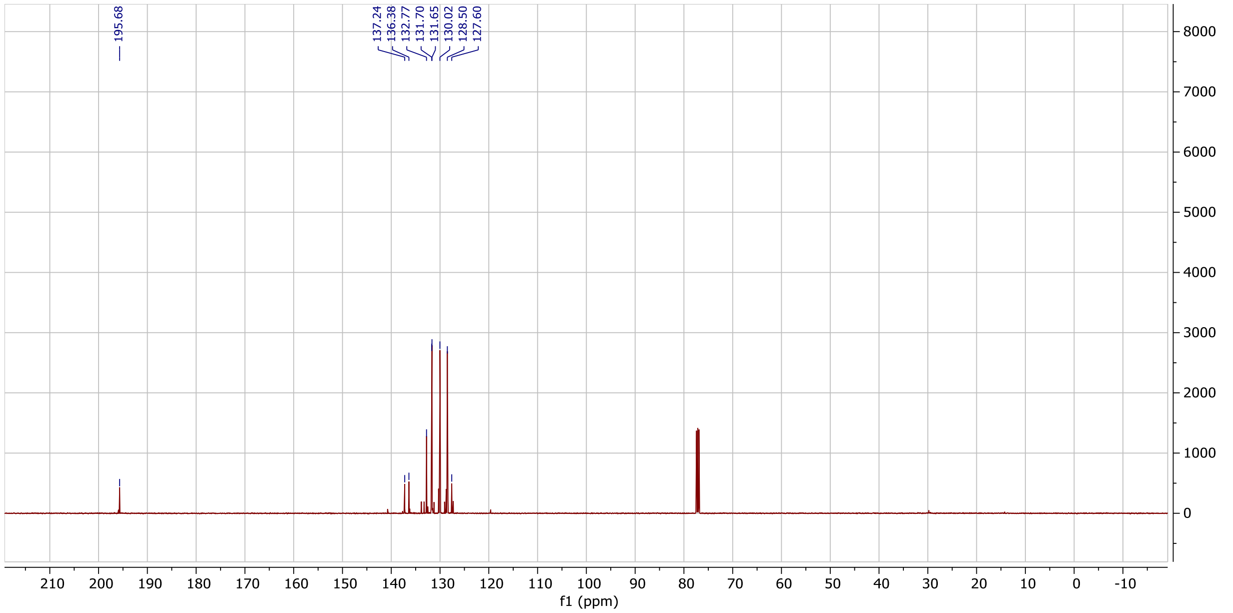

^{1}H NMR and ^{13}C NMR of 2f

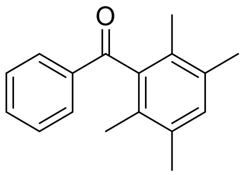

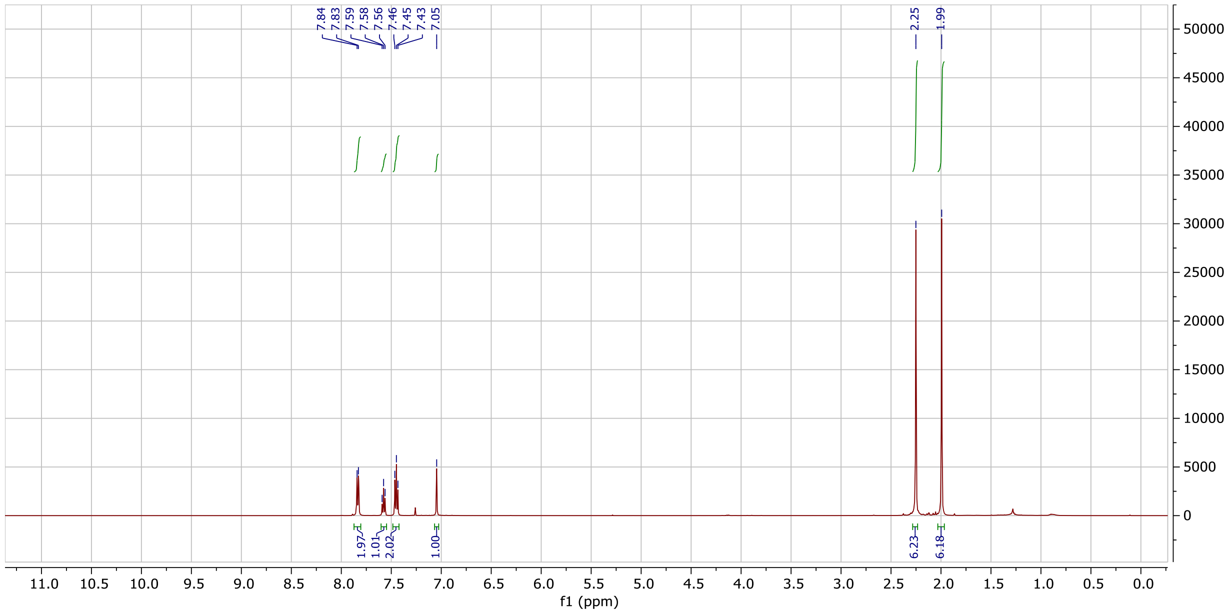

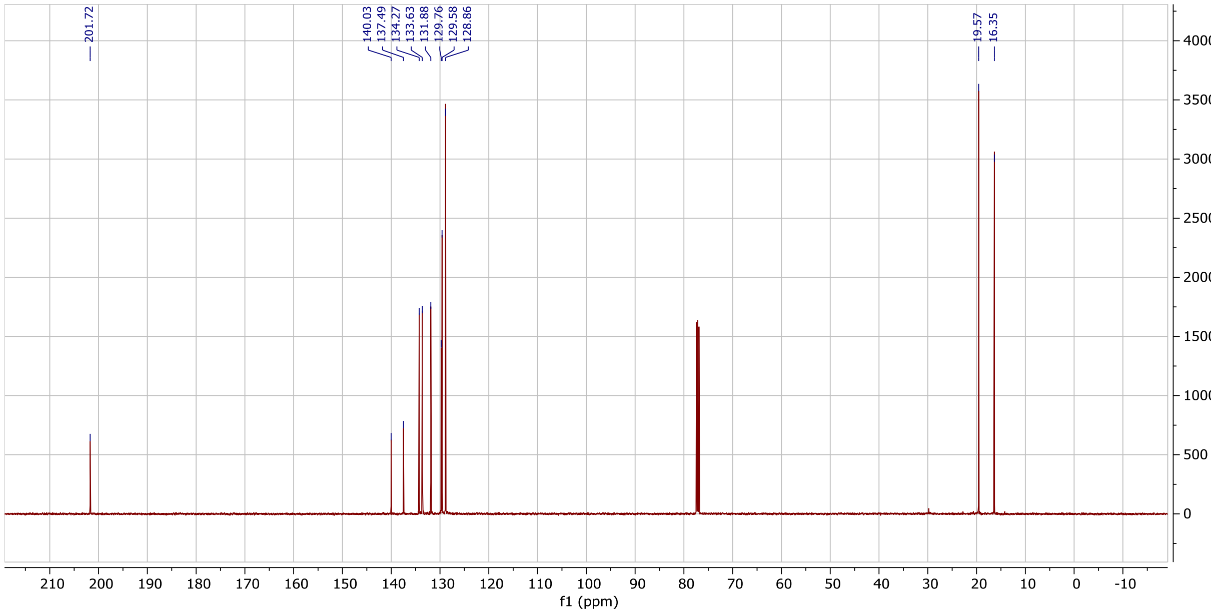

^{1}H NMR and ^{13}C NMR of 2g

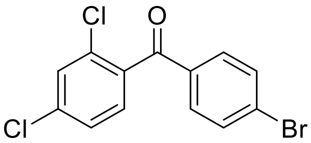

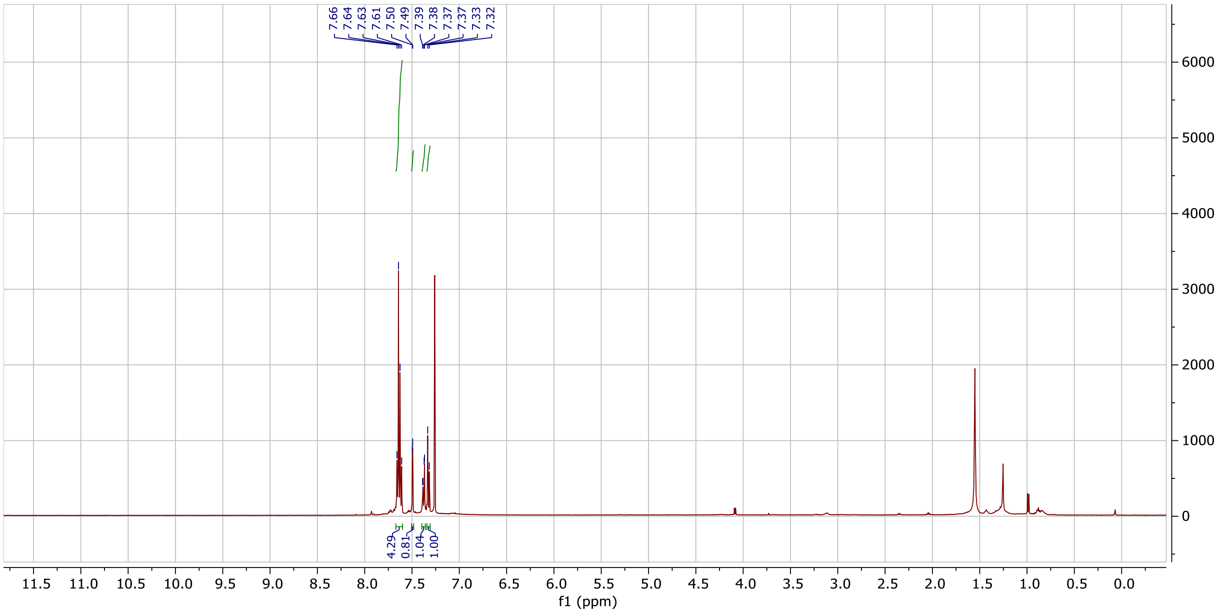

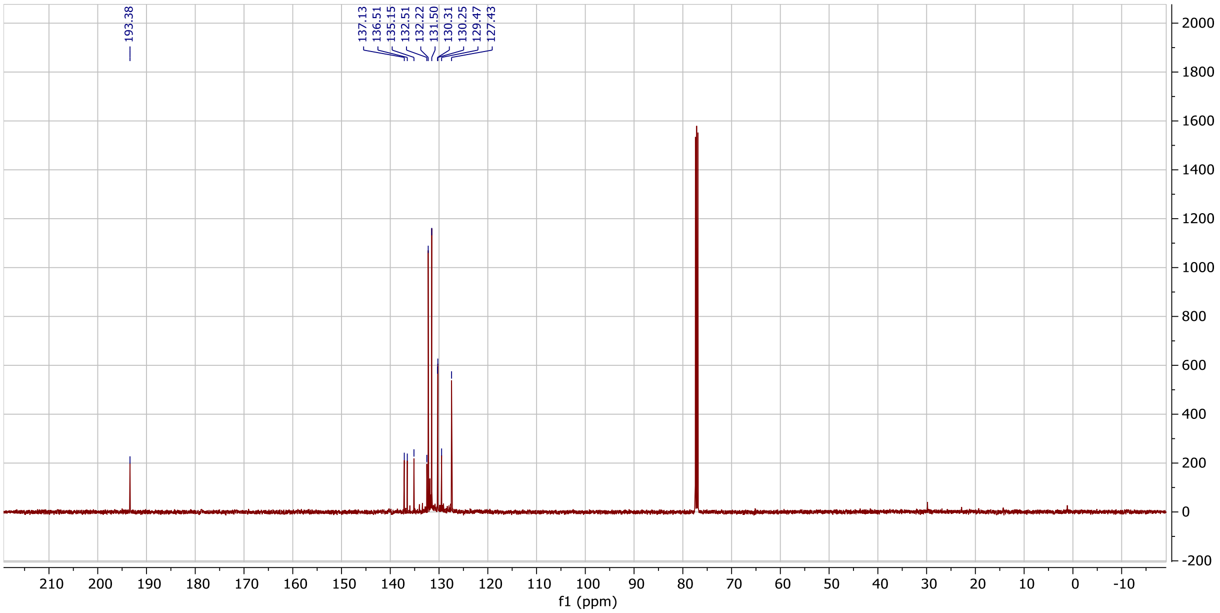

^{1}H NMR and ^{13}C NMR of 2h

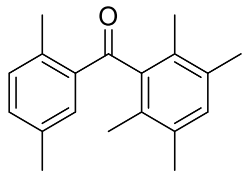

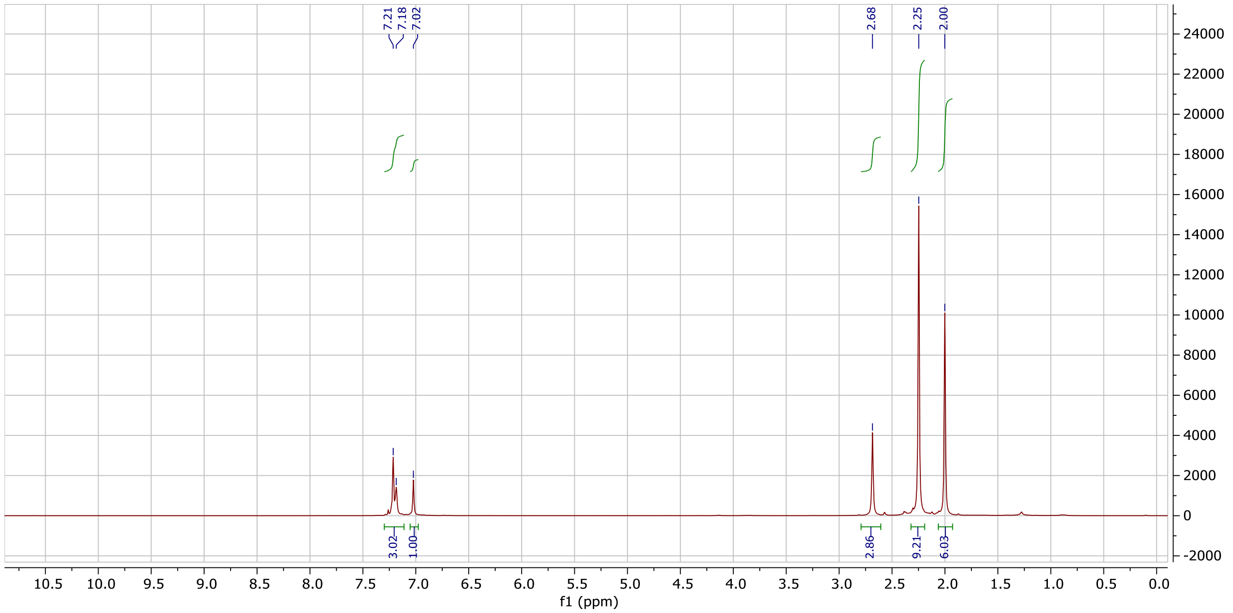

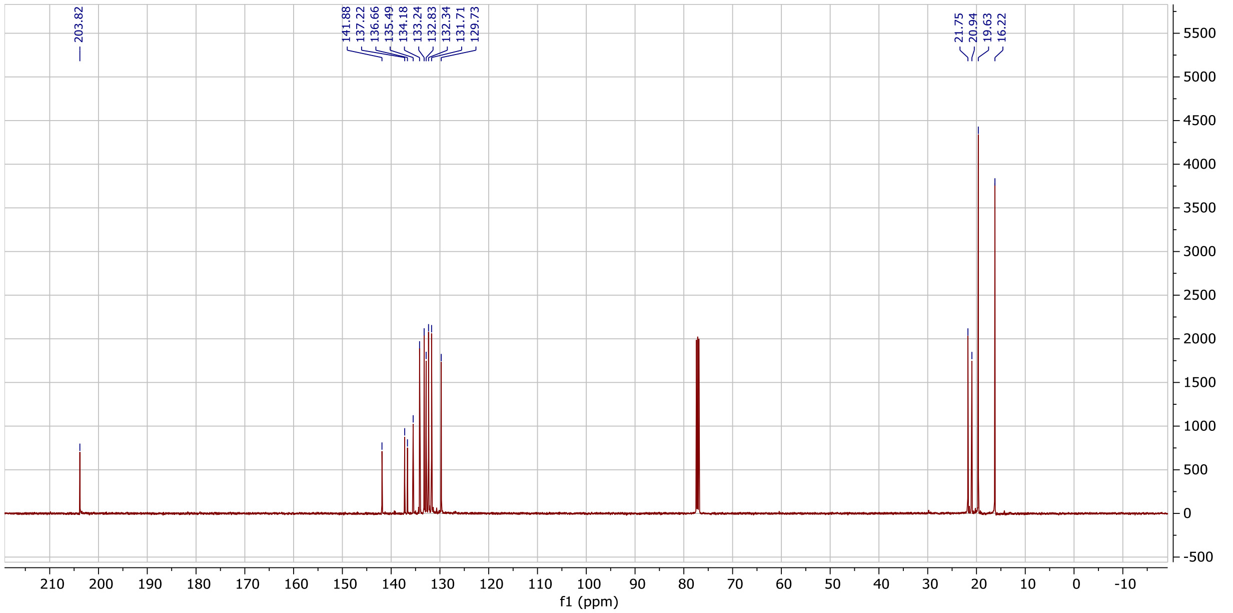

^{1}H NMR and ^{13}C NMR of 2i

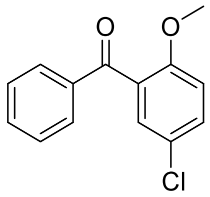

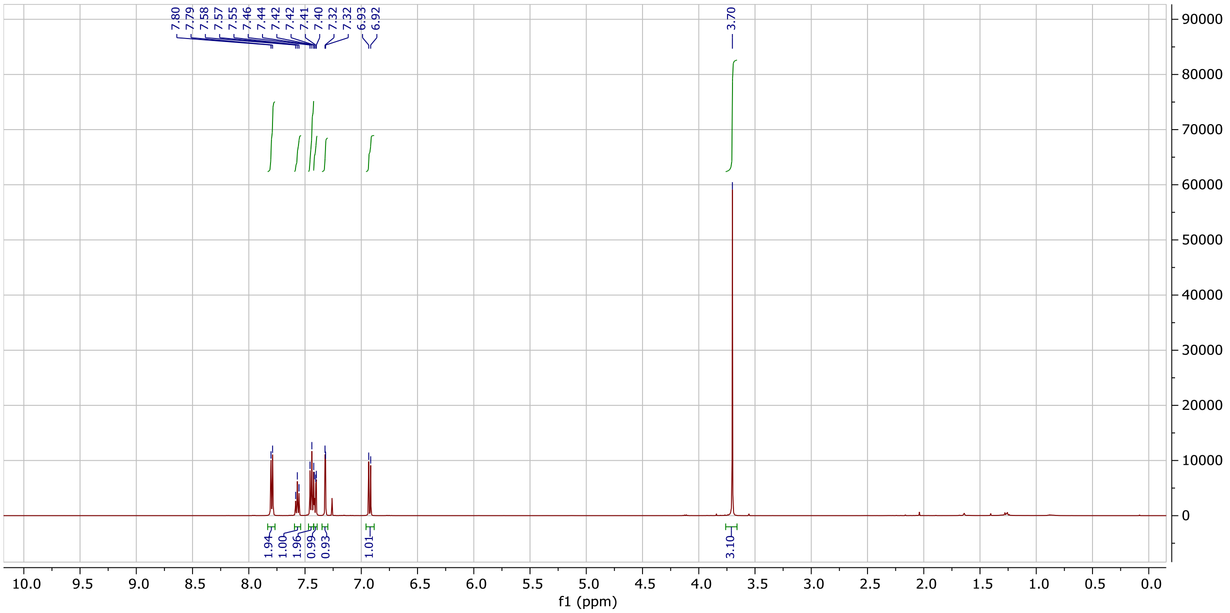

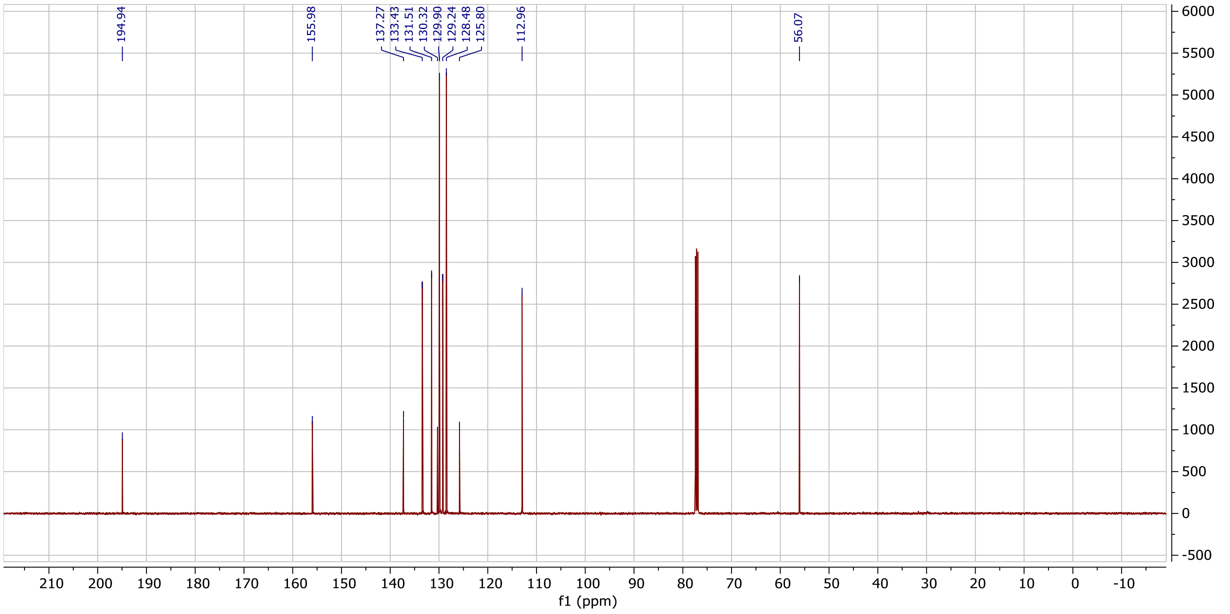

^{1}H NMR and ^{13}C NMR of 2j

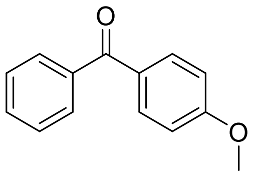

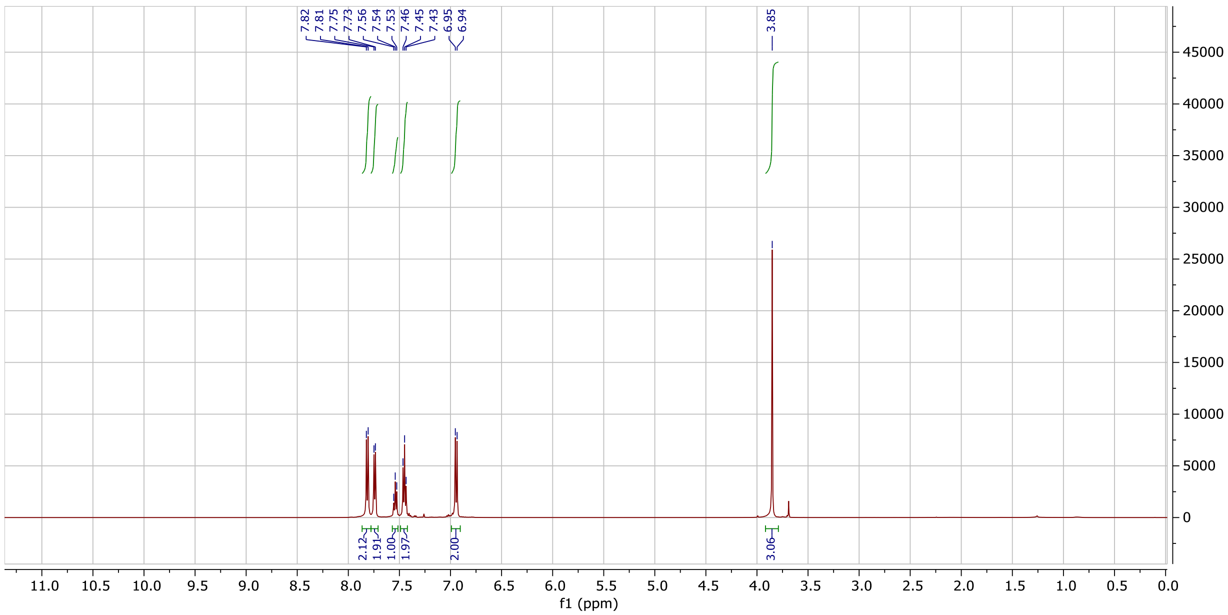

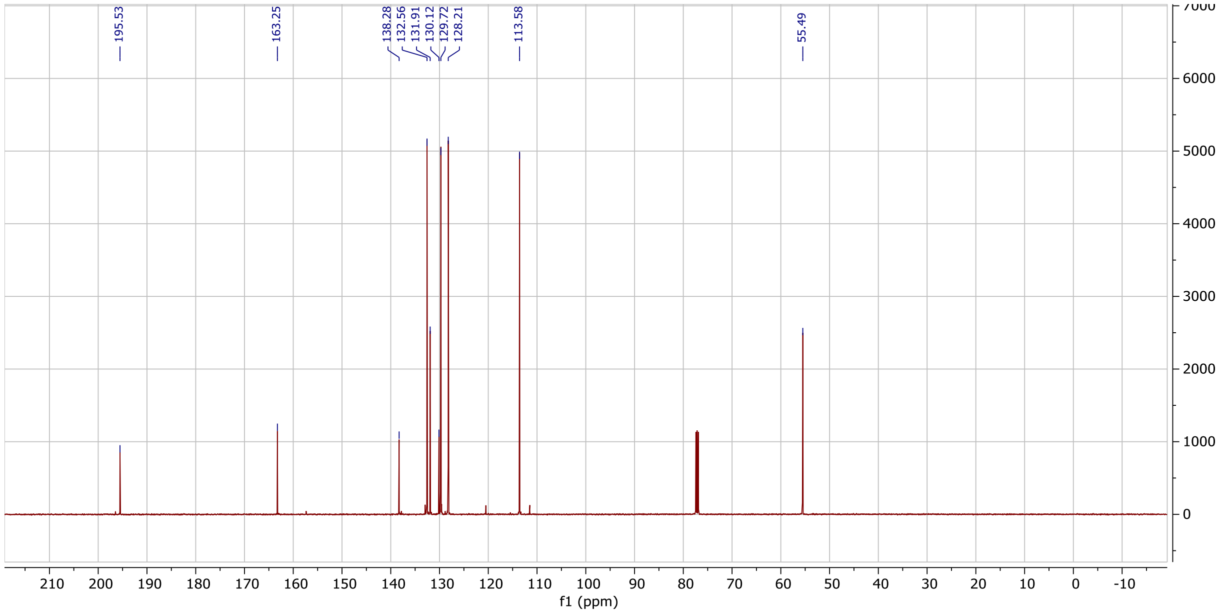

^{1}H NMR and ^{13}C NMR of 2k and 2k'

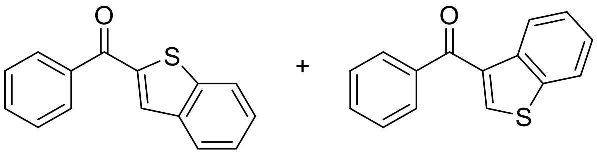

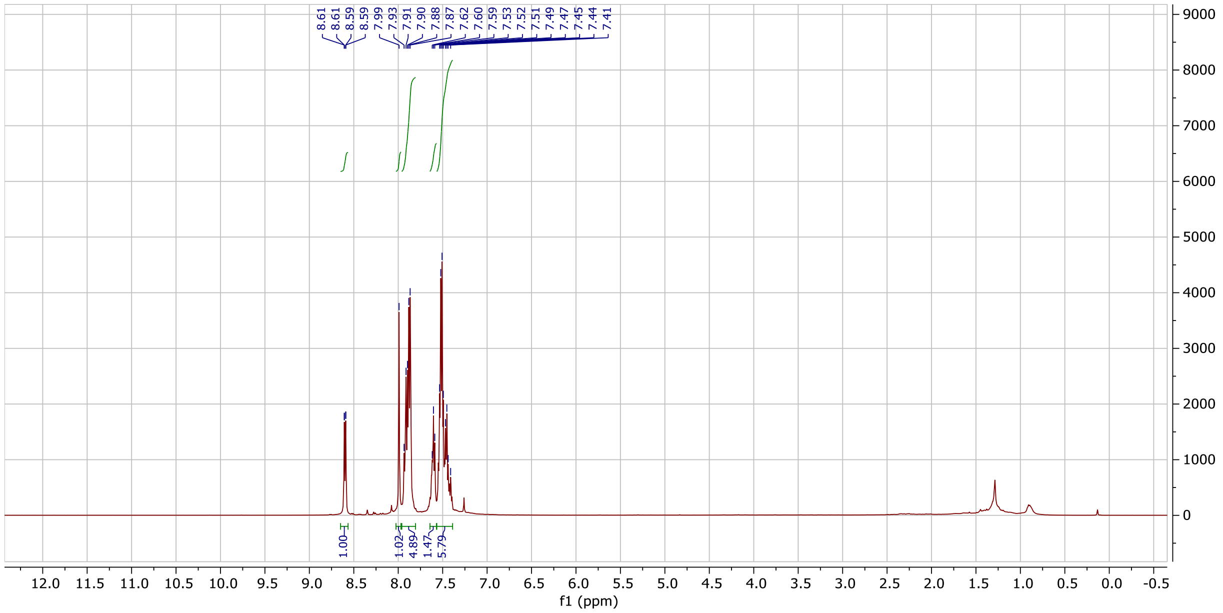

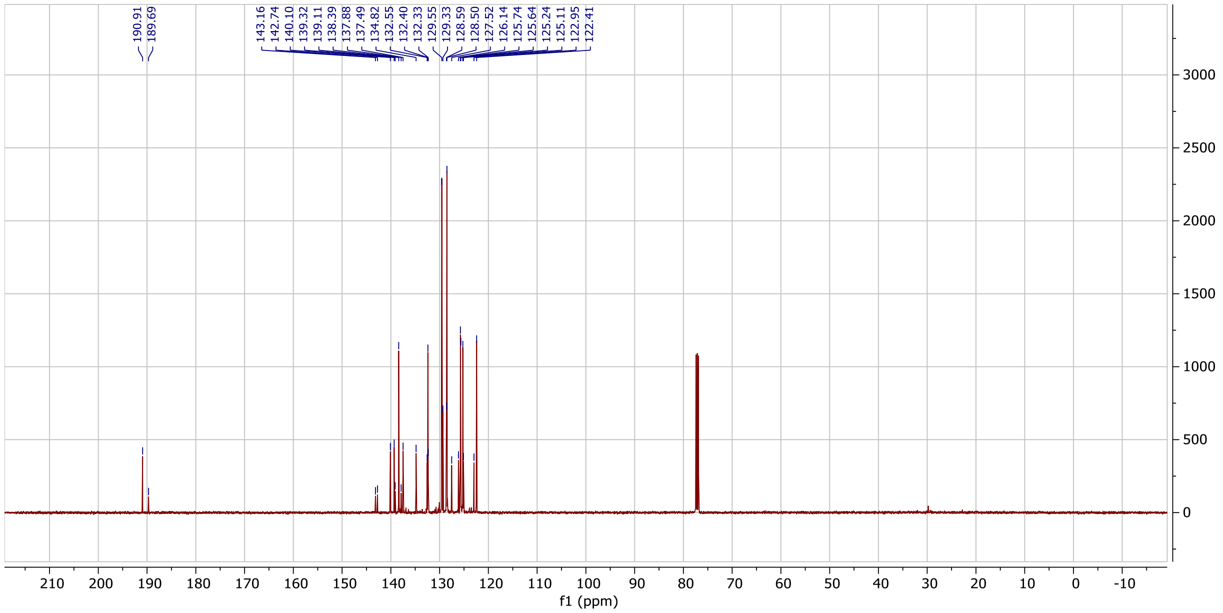

^{1}H NMR and ^{13}C NMR of 2l

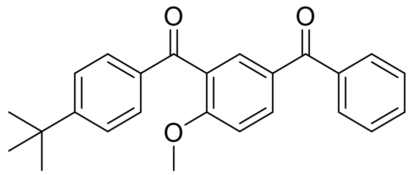

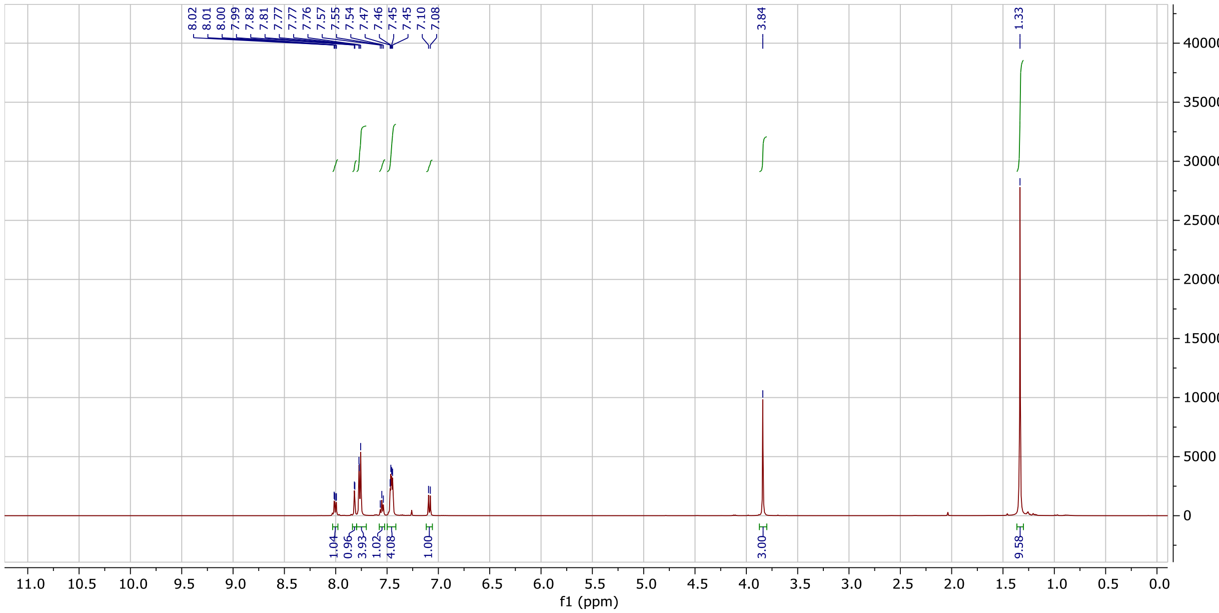

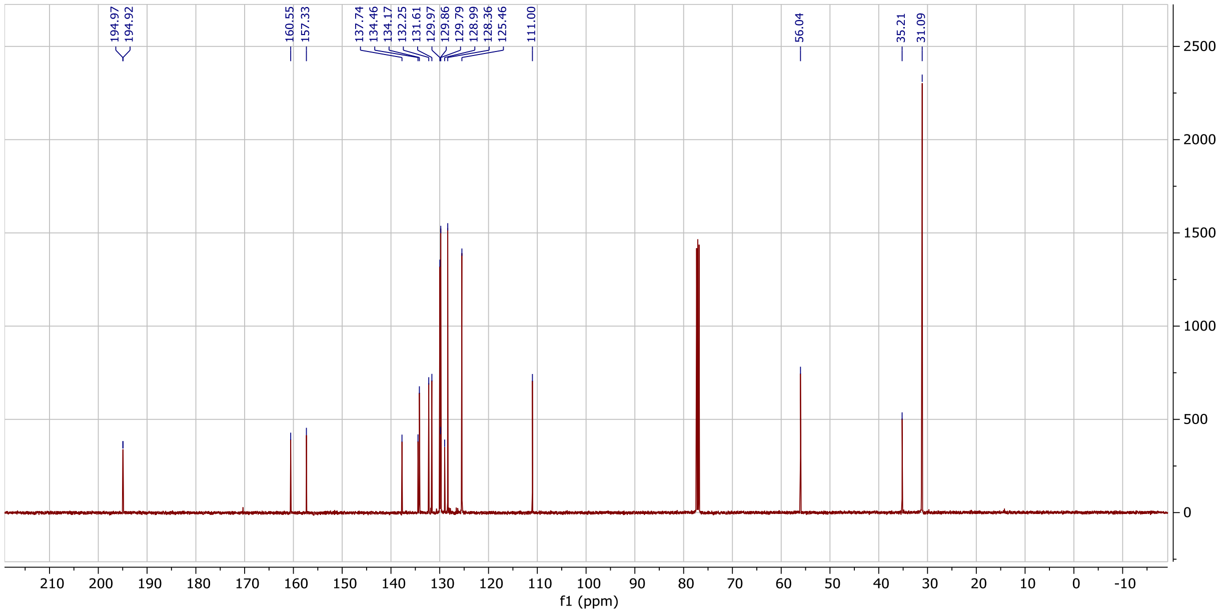

^{1}H NMR and ^{13}C NMR of 2m

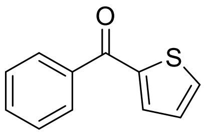

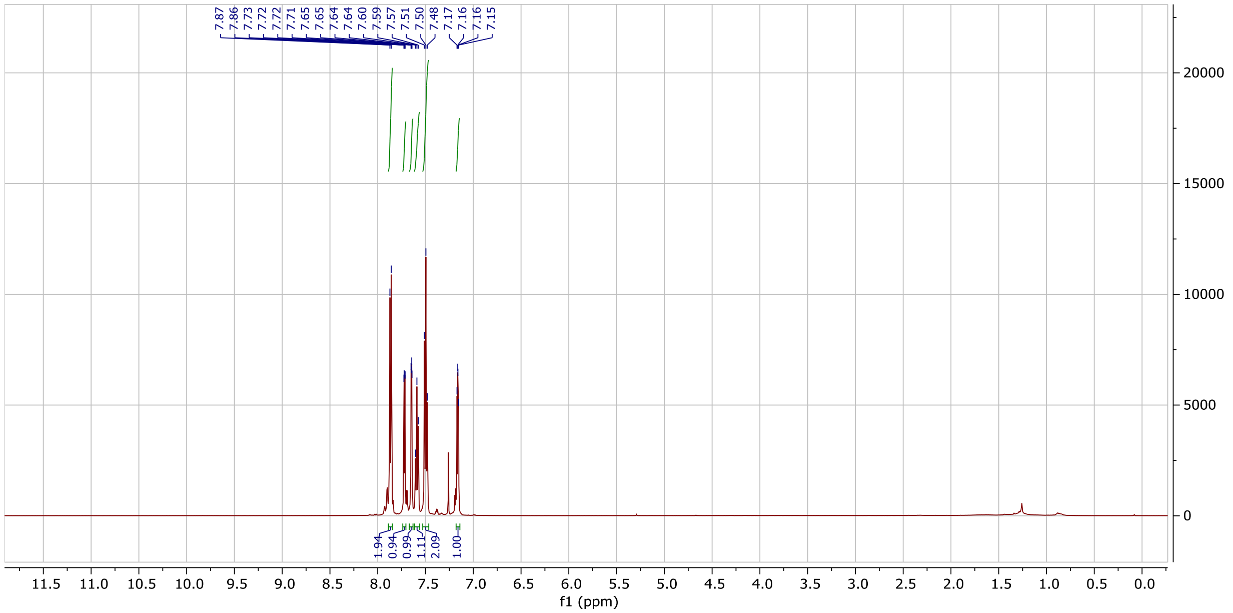

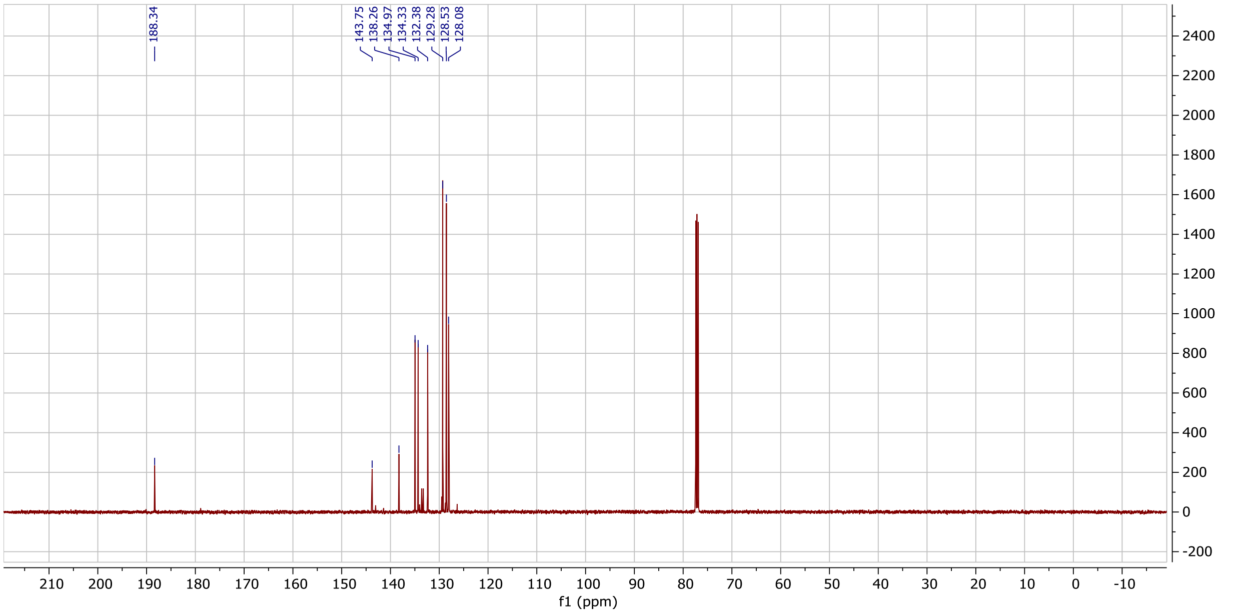

^{1}H NMR and ^{13}C NMR of 2n

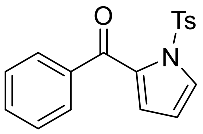

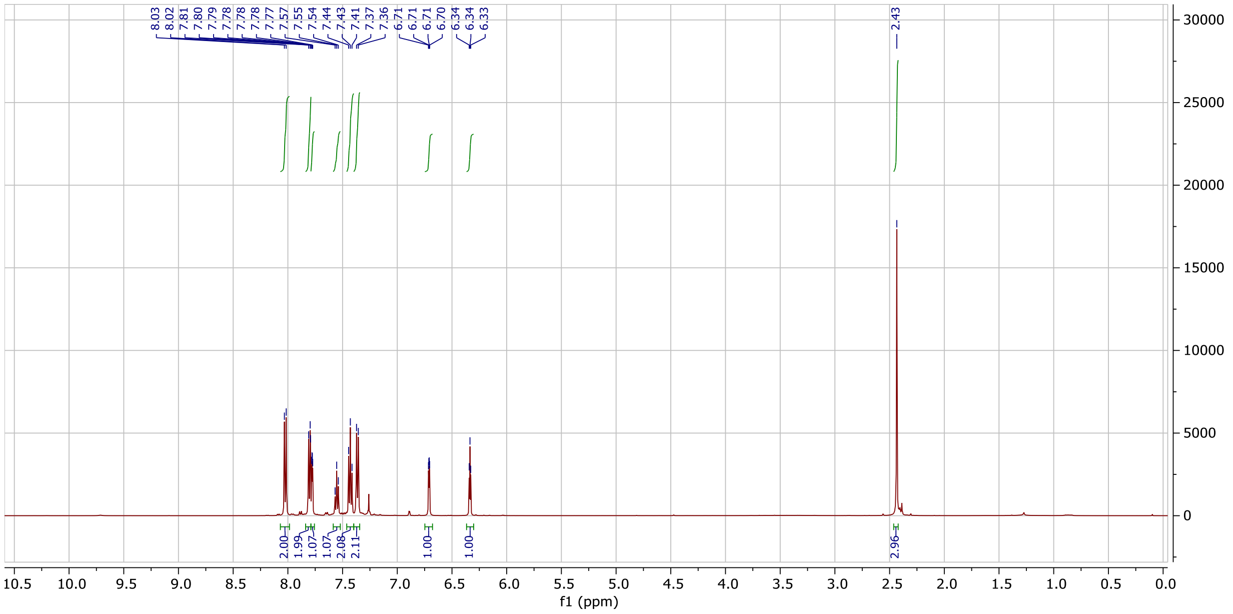

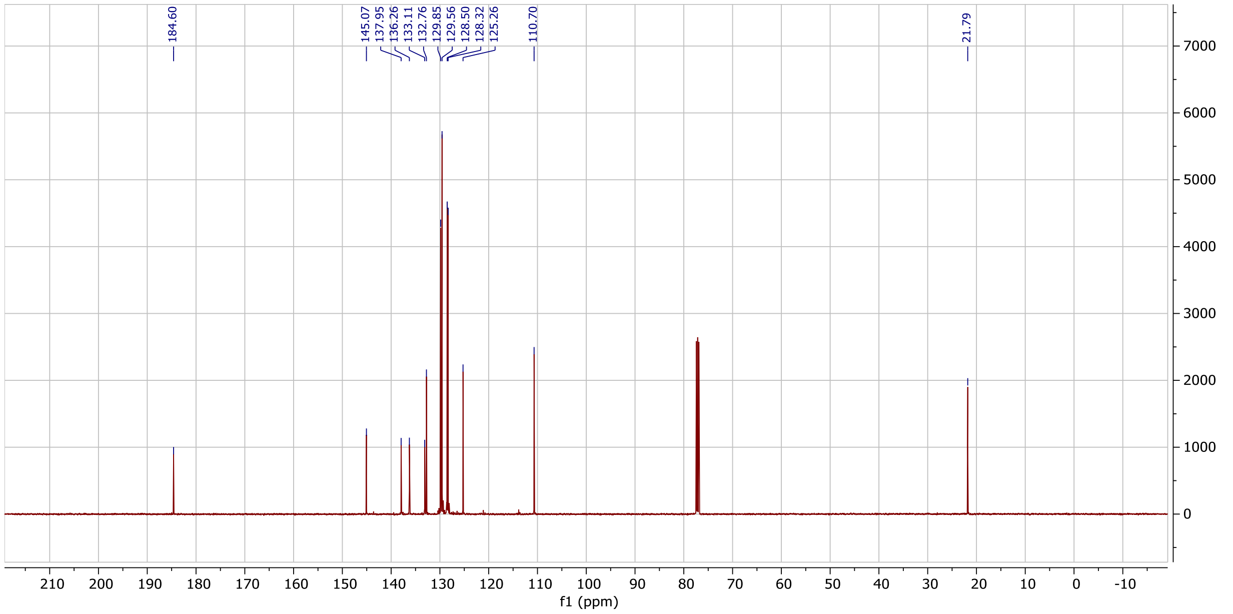

^{1}H NMR and ^{13}C NMR of 2o

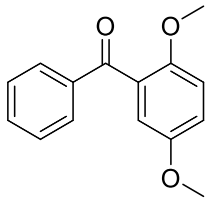

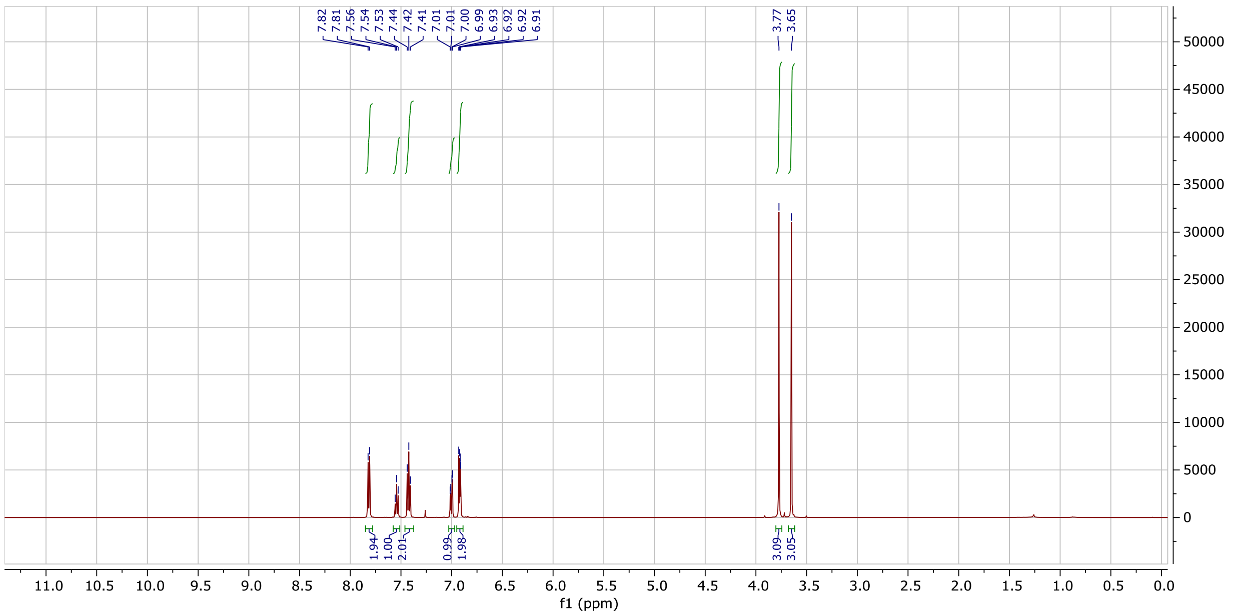

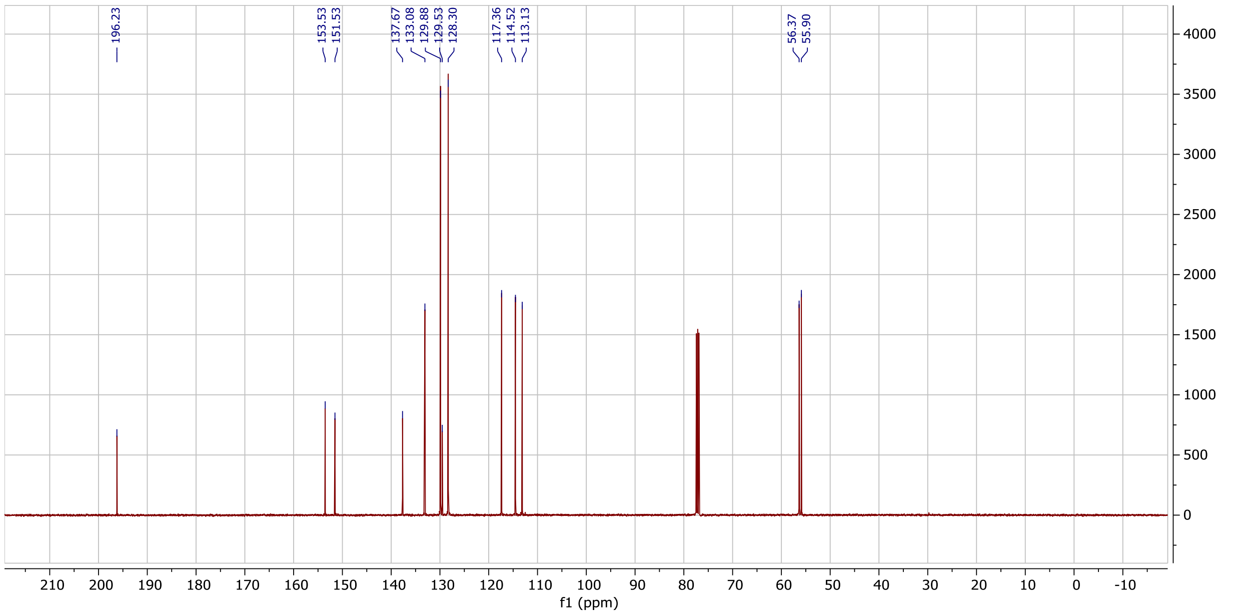

^{1}H NMR and ^{13}C NMR of 2p

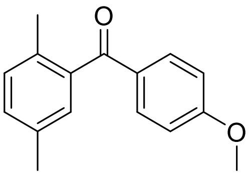

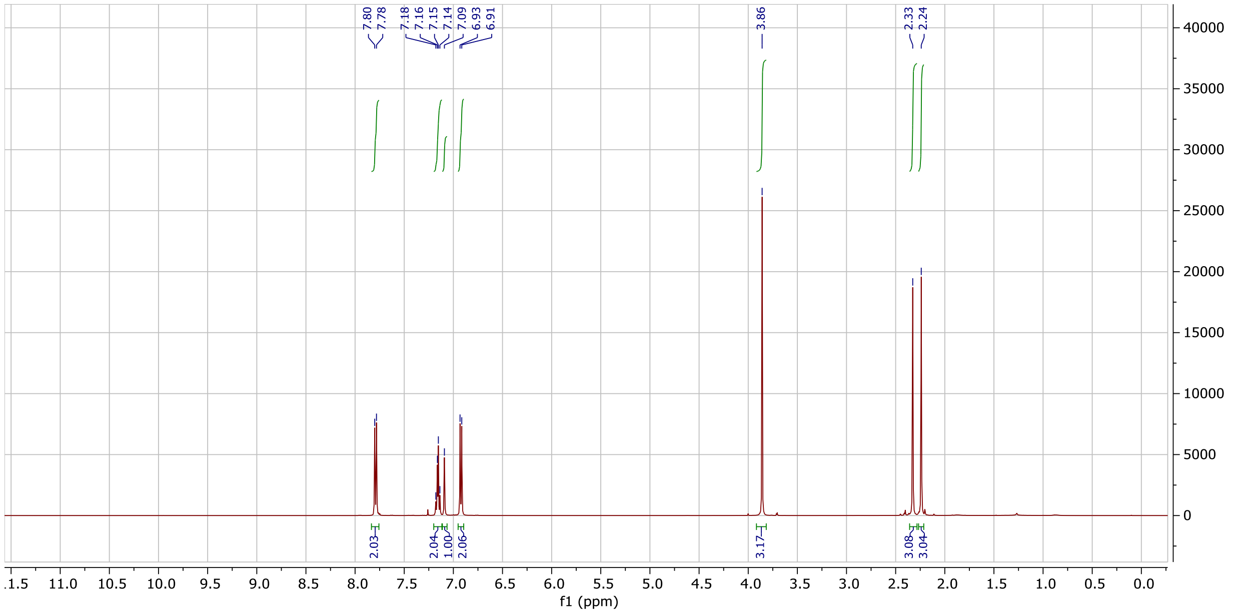

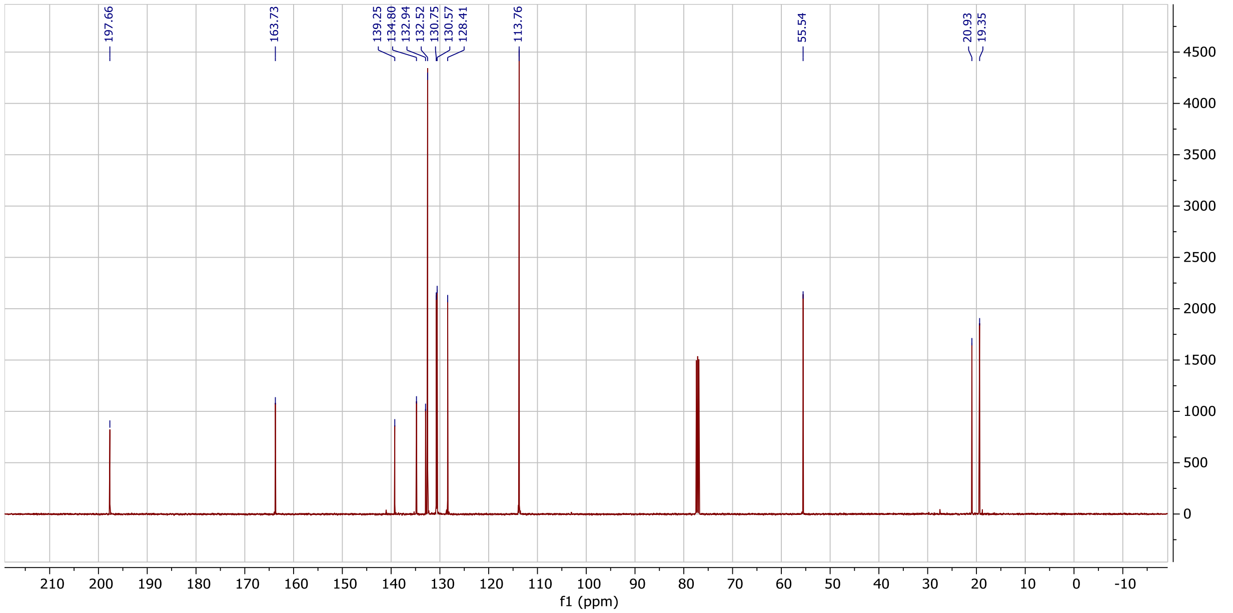

^{1}H NMR and ^{13}C NMR of 2q

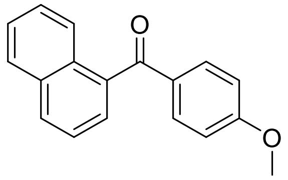

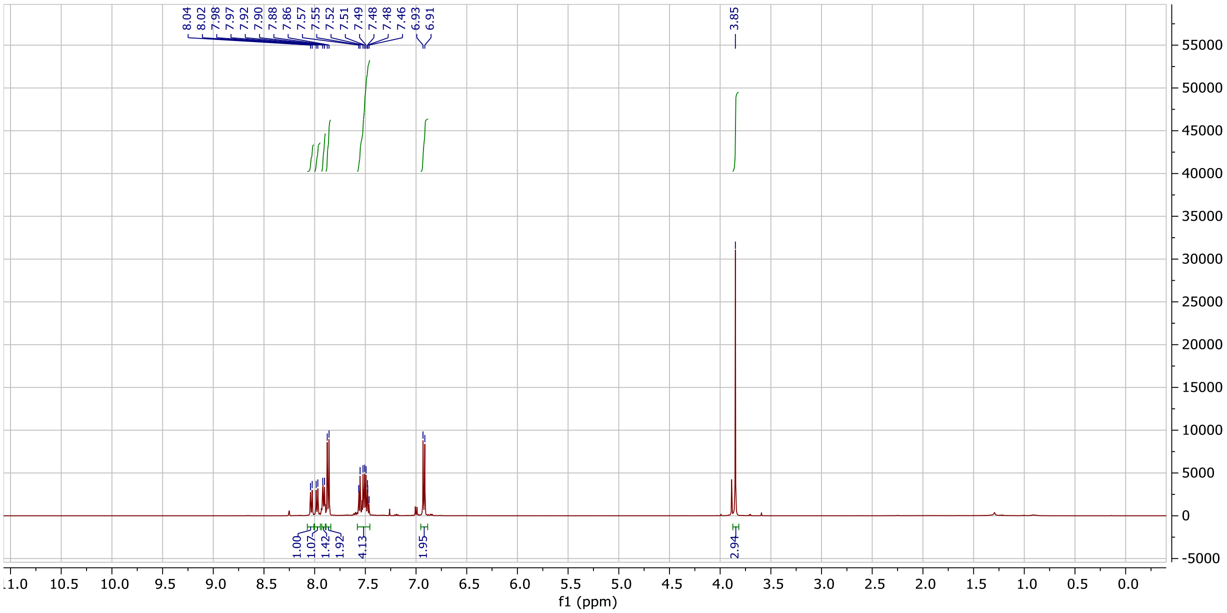

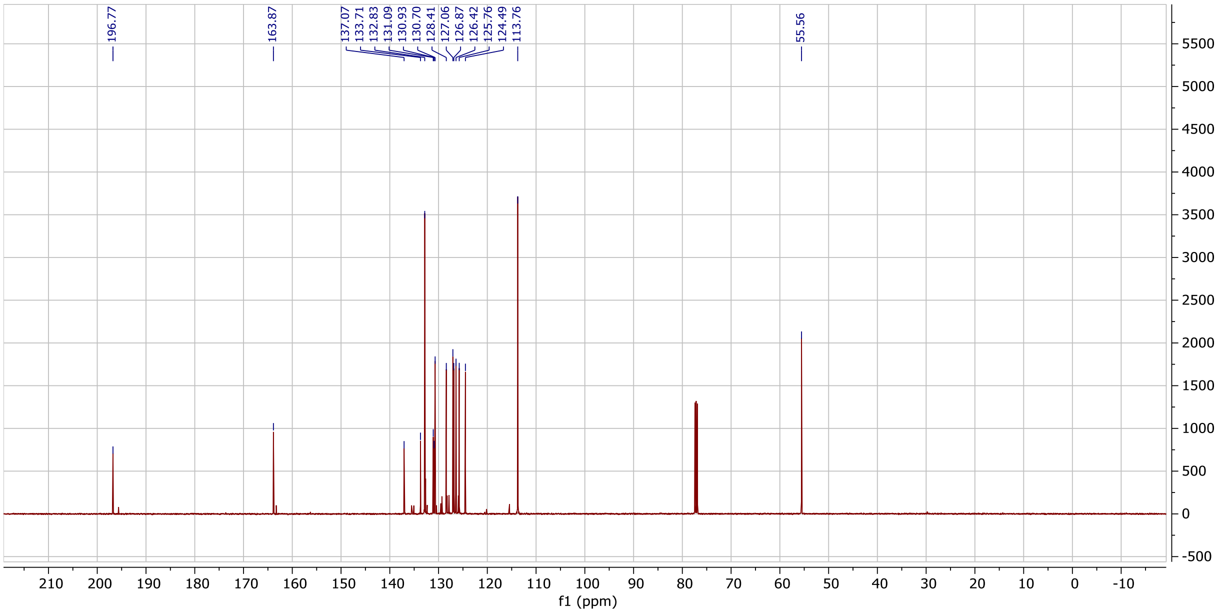

^{1}H NMR and ^{13}C NMR of 3 (R = Me)

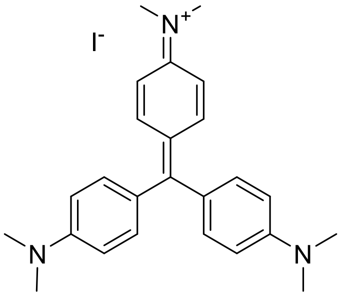

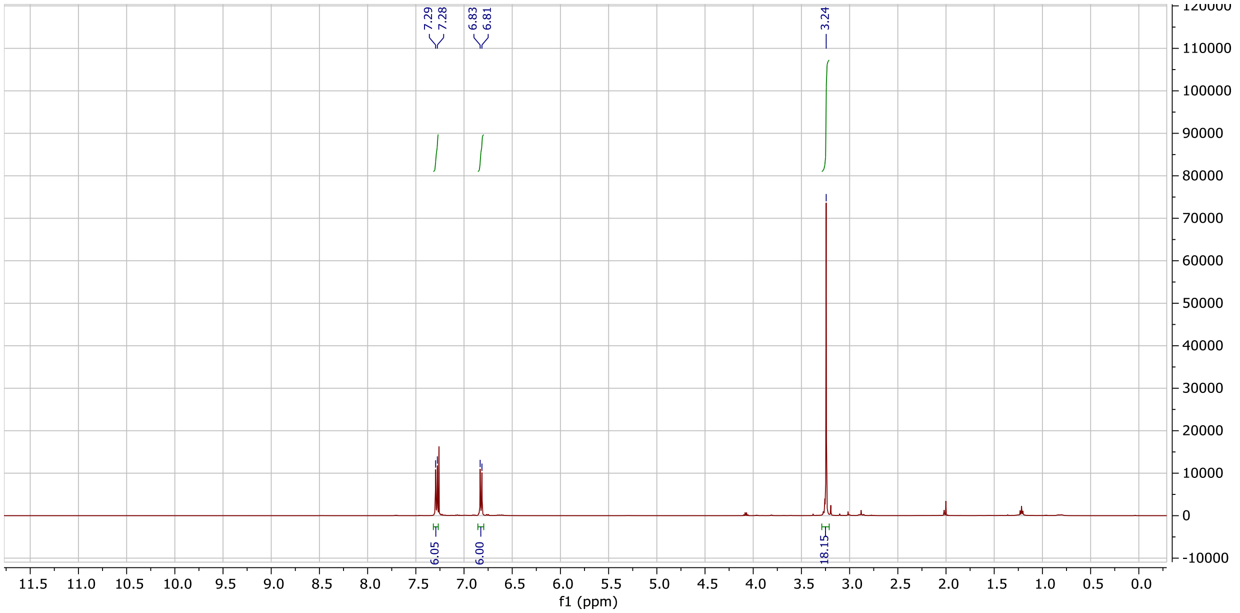

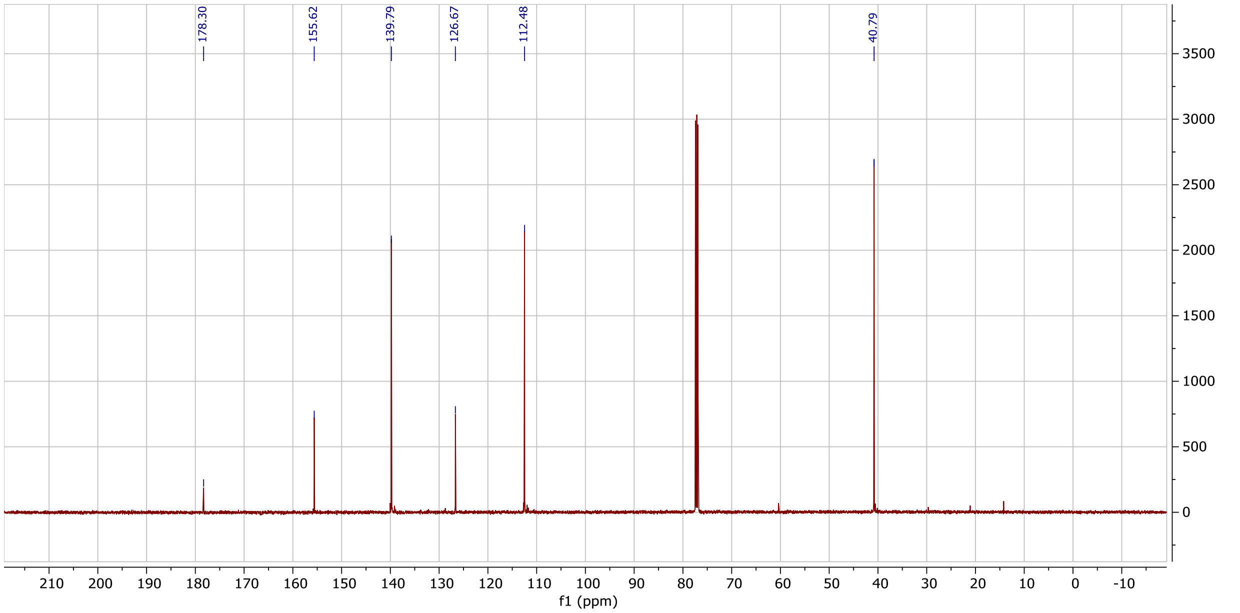

^{1}H NMR and ^{13}C NMR of 3’ (R = H)

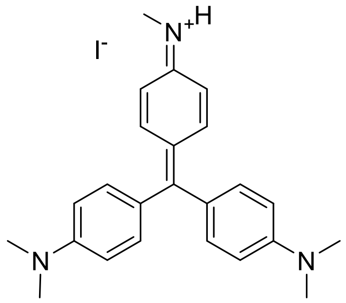

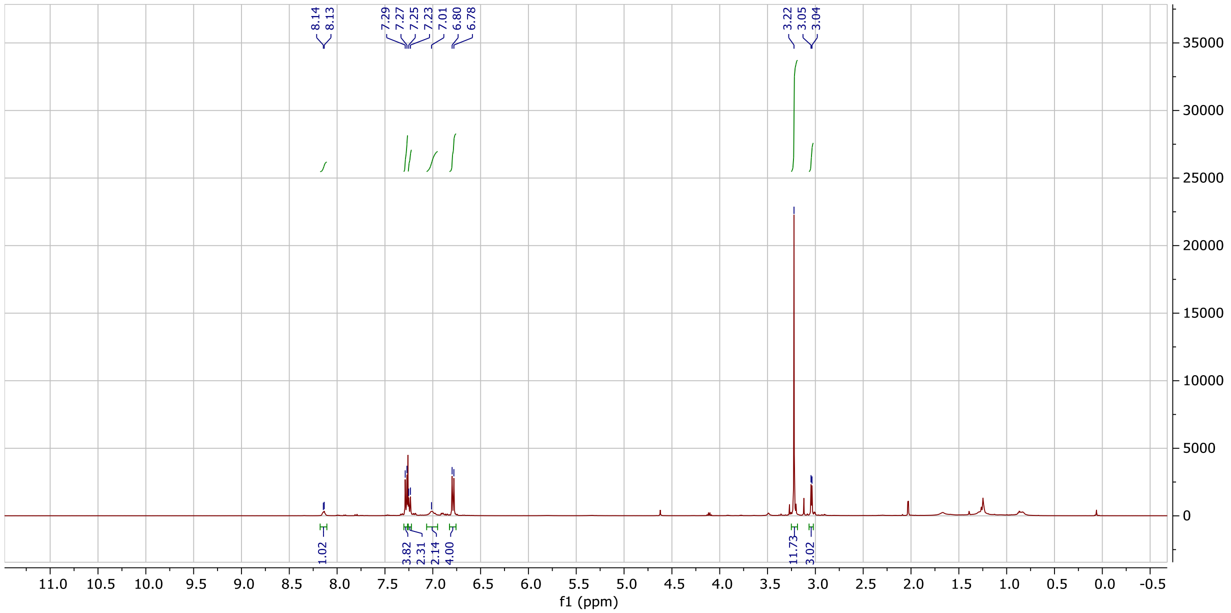

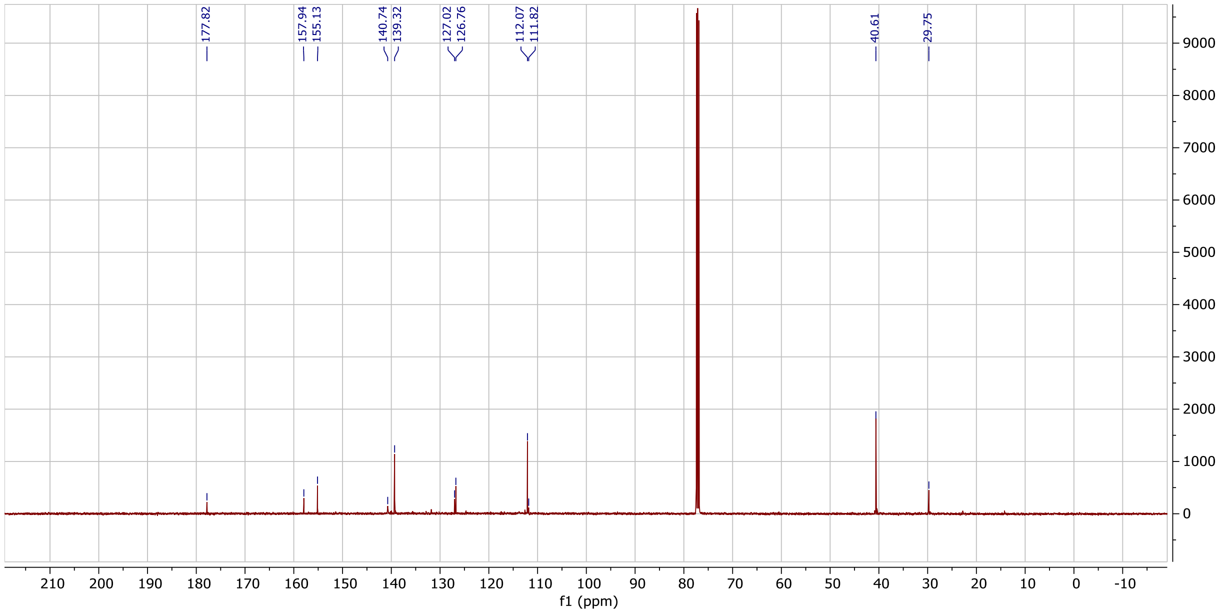

Supplement: SC-011-D0SC00085J-s001 [file SC-011-D0SC00085J-s001.pdf]
